# The Efficacy of Individual Cognitive Behavioral Therapy for Eating Disorders: A Meta‐Analysis of Randomized Controlled Trials

**DOI:** 10.1002/eat.24519

**Published:** 2025-08-14

**Authors:** Jana Bruns, Marieke Meier, Katrin Jansen

**Affiliations:** ^1^ University of Münster Münster Germany

**Keywords:** anorexia nervosa, binge‐eating disorder, bulimia nervosa, cognitive behavioral therapy, eating disorders, efficacy, meta‐analysis

## Abstract

**Background:**

This meta‐analysis aims to provide an update on the efficacy of individual cognitive behavioral therapy (CBT) for eating disorders (EDs) in the light of recent advances in the field, particularly the emergence of new approaches like self‐help.

**Method:**

We conducted multivariate multilevel meta‐analyses using data from 42 randomized controlled trials to evaluate the efficacy of individual CBT for anorexia nervosa, bulimia nervosa, binge‐eating disorder, and mixed eating disorder samples. Outcomes included ED pathology, the frequency of binge eating and compensatory behaviors, and body mass index. The type of comparison group, treatment format, treatment duration, baseline ED severity, and year of publication were analyzed as potential moderators.

**Results:**

CBT was more efficacious with regard to all relevant outcomes than waitlist conditions. For anorexia nervosa, there were no studies using a waitlist condition. CBT was not superior to active treatments for any disorder or any outcome. Therapist‐led treatment for bulimia nervosa showed a significant effect for all outcomes, while self‐help formats only did for some outcomes. Treatment duration and publication year were significant moderators for mixed EDs, while greater baseline severity was associated with larger effects of CBT for binge‐eating disorder.

**Discussion:**

The variety of active controls impeded comparative analyses of CBT with specific active controls. Effects were heterogeneous; the majority of studies had a poor risk of bias assessment. There is a paucity of research, particularly of treatment studies for anorexia nervosa.

**Summary**

- Cognitive behavioral therapy (CBT) is efficacious for eating disorders.
- For bulimia nervosa, CBT was more consistently (i.e., across multiple outcomes) efficacious when delivered by therapists. For mixed ED samples, guided self‐help was consistently efficacious.
- There are not enough trials investigating CBT for anorexia nervosa to make robust claims about its efficacy.

## 1 Background

Eating disorders (EDs) such as anorexia nervosa (AN), bulimia nervosa (BN), and binge‐eating disorder (BED) are heterogeneous mental illnesses (Diagnostic and Statistical Manual [DSM‐5]; American Psychological Association 2013) that have detrimental physical, psychological, and social consequences (Engel et al. 2009) even for those not meeting full diagnostic criteria (i.e., other specified EDs or unspecified EDs). EDs are associated with a 3.4 mortality ratio in comparison to the general population (Krug et al. 2025), show high comorbidity rates of about 70% (Demmler et al. 2020; Keski‐Rahkonen and Mustelin 2016), and go along with 48% higher healthcare costs compared with those of the average population (van Hoeken and Hoek 2020) as well as significant consequences for caregivers (Martín et al. 2015). Given these numbers, it is important to know which treatments are efficacious in reducing ED pathology, for example, distorted cognitions and behaviors such as binge eating and compensatory behaviors.

While most evidence‐based national treatment guidelines underline the necessity of a multimodal treatment approach comprising nutritional counseling, occupational therapy, and potentially medication, psychotherapy is the cornerstone treatment for EDs (Hilbert et al. 2017). Most treatment guidelines recommend cognitive behavioral therapy (CBT) in an outpatient setting (Hilbert et al. 2017) as the first line of treatment, where individual therapy is more common than group therapy (Schopf et al. 2023). This mirrors findings from randomized‐controlled trials (RCTs) that attest to the efficacy of CBT when treating BN (e.g., Agras et al. 2000; Hartmann et al. 2024; Poulsen et al. 2014) and BED (e.g., Melisse et al. 2023; Wagner et al. 2016). For AN, guidelines are less univocal (Hilbert et al. 2017) as RCTs including patients with AN are scarce and do not consistently show superiority of CBT compared to other treatments (e.g., Dalle Grave et al. 2013; Zipfel et al. 2014).

Because individual studies sometimes fail to provide conclusive evidence, for example, due to small sample sizes, meta‐analyses have been conducted with the goal to clarify treatment efficacy of CBT in comparison to other treatments. These meta‐analyses have also attempted to identify factors that moderate the effect of treatment, for example, diagnosis, treatment duration, or comorbid conditions. Meta‐analyses synthesizing the evidence of psychosocial treatments for EDs have been mostly conducted separately for AN (e.g., van den Berg et al. 2019; Zeeck et al. 2018), BN (e.g., Slade et al. 2018), and BED (e.g., Hilbert et al. 2020). The most recent meta‐analysis with a broader, more transdiagnostic focus was conducted by Linardon, Wade, et al. (2017) and investigated the efficacy of CBT for AN, BN, and BED across 79 RCTs. The authors found that, overall, CBT was efficacious in comparison to inactive controls. For BN, there was evidence that individual therapist‐led CBT was more efficacious for binge eating/purging than other active treatments and more efficacious regarding cognitive symptoms and binge‐eating/purging frequency. Studies were not sufficient to investigate the efficacy of CBT administered via (guided) self‐help (GSH). For BED, therapist‐led CBT was more efficacious than inactive controls regarding binge eating while it was not superior to other active treatments. When administered in the form of self‐help (SH), CBT did not show greater efficacy than active treatments. For AN, there was no difference at post treatment between CBT and active treatments regarding cognitive symptoms.

A recent umbrella review regarding the efficacy of treatments for EDs that included a total of 884 studies concluded that individual CBT was more efficacious than other psychotherapies when treating BN (Monteleone et al. 2022). For BED, the authors found that CBT was more efficacious than other psychotherapies for ED behaviors but not for cognitions or remission status. For AN, the authors were able to identify family‐based therapy as evidence‐based treatments for adolescents and young adults while they found no superiority of other treatments for adults. Regarding follow‐up, the umbrella review reported sustained treatment effects for BN and BED after undergoing psychotherapy but not medication.

Despite their contributions, prior meta‐analyses have limitations. Many have focused narrowly on specific ED diagnoses or have assessed broad, conflated outcomes (e.g., combined binge‐eating/purging behavior, or generalized “ED behaviors” across settings). Moreover, moderators of treatment efficacy were not consistently investigated.

Recent developments in the field further underscore the need for an updated synthesis. The inclusion of BED in the DSM‐5 (APA 2013) and the 11th version of the International Classification of Disease (ICD‐11; World Health Organization 2022) stimulated the intensified investigation of valid treatment options for BED. Moreover, new psychosocial treatment options such as third wave psychotherapies (e.g., Ben‐Porath et al. 2020; Brown et al. 2020; Haynos et al. 2016) and treatment formats such as self‐help have emerged (e.g., Wilson and Zandberg 2012; Yim and Schmidt 2019). Since the publication of Linardon, Wade, et al.'s (2017) meta‐analysis, nearly a dozen new RCTs on CBT for EDs have been published (e.g., Hartmann et al. 2024; Melisse et al. 2023), further expanding the evidence base.

Taken together, these limitations and recent advancements highlight the necessity for an updated, comprehensive meta‐analysis that incorporates the latest evidence, distinguishes between diagnoses and treatment formats, investigates moderators, and assesses key disorder‐specific outcomes, including weight gain in AN.

### 1.1 The Present Meta‐Analysis

This meta‐analysis aims to update the meta‐analysis by Linardon, Wade, et al. (2017) by focusing on RCTs that investigated the efficacy of individual CBT for EDs. Meta‐analytic findings (Monteleone et al. 2022) underline the necessity to differentiate between diagnoses and to include different outcomes (behavioral and cognitive/affective) when evaluating treatment efficacy. Hence, we planned to conduct separate analyses for AN, BN, and BED and to further include a separate analysis for transdiagnostic samples and samples comprising EDs not otherwise specified (EDNOS) and other specified feeding and eating disorders (OSFED). Moreover, for each diagnosis, we considered outcomes reflecting its particular psychopathology (i.e., ED pathology and body mass index (BMI) for AN; ED pathology and frequency of binge eating and compensatory behaviors for BN; ED pathology and frequency of binge eating for BED). We added BMI as an outcome for AN as weight restoration is a key target for treatment (Hilbert et al. 2017).

In this study, the term *ED pathology* refers to the overall severity of ED‐associated cognitions, feelings, and behaviors, with most measures usually putting an emphasis on cognitive and affective symptoms (e.g., the Eating Disorder Examination [Questionnaire], EDE [‐Q]; Fairburn and Beglin 1994, 2008). Hence, ED pathology is a broader and less closely defined outcome than the frequency of specific behaviors or BMI. In our meta‐analysis, we categorized both purging behaviors (such as self‐induced vomiting or laxative abuse) and other compensatory, non‐purging behaviors (like using diet pills, restrictive eating, or driven exercise, see Abebe et al. 2012) as compensatory behaviors.

We decided to solely include studies focusing on individual therapy as it is most disseminated and as group therapy and individual therapy should best be compared head‐to‐head; that is, within one trial (Grenon et al. 2017). Moreover, group therapy is often used in inpatient settings to complement individual therapy or as an initial step for patients with less severe ED pathology, such that its inclusion would introduce significant variability, further complicating result interpretation that is already difficult given heterogeneity (Linardon, Wade, et al. 2017).

In addition to updating the meta‐analysis by Linardon, Wade, et al. (2017), we planned to expand on their search strategy by contacting authors whenever additional data was needed for including a particular study. Moreover, we attempted to address potential biases that might have been overlooked in previous meta‐analyses. Previous research showed that meta‐analyses may overestimate effects of psychotherapy because studies with low quality can produce overly optimistic results (Cuijpers et al. 2010). Hence, we considered it important to establish a lower bound for the efficacy of CBT, which we did by conducting a sensitivity analysis based on studies with high quality only. A further factor which may introduce bias in meta‐analytic effects is differential attrition (Molenberghs and Kenward 2007). The impact of this can be assessed by comparing the results from studies reporting intent‐to‐treat (ITT) data to those from completers (cf., e.g., Rutherford et al. 2012). Based on similar ideas, we preferred ITT over completer data if both were available, and conducted sensitivity analyses including only studies that reported ITT data to assess the potential impact of differential attrition. Lastly, we searched for gray literature (e.g., unpublished work).

Moderators for treatment response identify for which patient groups or under which conditions treatment is efficacious (Kraemer et al. 2002), which guides clinical decision making and can inspire theories about mechanisms of treatments. Previous meta‐analyses on treatment efficacy in EDs often did not have sufficient data to conduct moderator analyses (e.g., Brownley et al. 2007; Linardon, de la Piedad Garcia, and Brennan 2017) or did not look at participant baseline characteristics (e.g., Ghaderi et al. 2018; Slade et al. 2018; Solmi et al. 2021; Zeeck et al. 2018), which represent areas we aimed to address. In our meta‐analysis, we considered the following moderators: (1) We assessed the type of comparison as a moderator, as the efficacy of CBT for EDs tends to differ depending on which kind of treatment CBT is compared to. While most meta‐analyses found a higher efficacy of CBT compared to inactive comparisons such as waitlist (e.g., for BED Hilbert et al. 2019; for BN Slade et al. 2018), CBT was usually not more efficacious in comparison to active comparisons, for example, other psychotherapy interventions, especially for AN (Monteleone et al. 2022; see Freedland et al. 2011 for a discussion of the use of control groups in RCTs). Hence, we expected greater effects for CBT in comparison to waitlist and treatment as usual (TAU) than in comparison to active treatments. (2) Furthermore, with the growing development of online and self‐help (SH) formats, we sought to examine treatment format as a potential moderator, especially in light of meta‐analytic evidence suggesting varying effects across different treatment formats (e.g., Slade et al. 2018). Specifically, while SH formats have mostly been used for the treatment of BN or BED and have been found efficacious in comparison to waitlist, Monteleone et al. (2022) indicated that therapist‐led treatment formats (especially therapist‐led CBT for BN) are more efficacious. Therefore, we hypothesized that therapist‐led treatment formats would yield greater efficacy than SH formats. (3) We examined treatment duration, measured in weeks, as a potential moderator. Across RCTs, the length of treatment often varies greatly (e.g., in a meta‐analysis on AN by van den Berg et al. 2019, it ranged from 0 to 58 outpatient sessions). Hence, meta‐analysis is well‐suited for examining its impact on treatment efficacy. So far, meta‐analytic accounts of treatment duration as a moderator for treatment efficacy in EDs are scarce. For BED, Hilbert et al. (2019) found that short‐term (i.e., < 10 sessions) treatments were more efficacious in reducing binge eating than long‐term treatments. Given the scarcity of meta‐analyses investigating treatment duration as a moderator and the null findings for other mental illnesses (e.g., for depression, Hofmann et al. 2017 and for obsessive‐compulsive disorder, Olatunji et al. 2013), we investigated treatment duration exploratorily. (4) Baseline severity represents a relevant factor that often guides clinical decision making, for example, around treatment recommendations. For several mental disorders, baseline severity has an impact on treatment response (e.g., Khan et al. 2002; Scholten et al. 2023). For EDs, studies paint a heterogeneous picture (Le Grange et al. 2008, 2014) and meta‐analyses point to insufficient data to make robust claims (Linardon, de la Piedad Garcia, and Brennan 2017; Vall and Wade 2015). Given the heterogeneous nature of these findings, we decided to investigate baseline severity exploratorily per diagnosis. (5) Lastly, we included publication year as a potential moderator to investigate trends in efficacy over the years. To summarize, we planned to conduct moderator analyses for the following variables: (1) the type of comparison (active treatment vs. waitlist vs. TAU), (2) the treatment format (therapist‐led vs. GSH vs. pure SH) (3) treatment duration, (4) baseline symptom severity, and (5) publication year.

Altogether, considering recent advances in the field, we aimed to update the meta‐analytic evidence regarding the efficacy of individual CBT for EDs by differentiating between diagnoses, conducting sensitivity analyses for ITT samples and high‐quality studies, and including a range of moderators for treatment outcomes.

## 2 Method

### 2.1 Identification of Studies

The present meta‐analysis was conducted in compliance with the Preferred Reporting Items for Systematic Reviews and Meta‐Analyses guidelines (Page et al. 2021). The online databases EBSCOHost (including Academic Search Ultimate, CINAHL, PsycArticles and PsycINFO) and PubMed were searched using the following search term: “((eating disord*) OR (anorexi*) OR (bulimi*) OR (binge*) OR (EDNOS) OR (OSFED)) AND ((CBT) OR (CBT‐E) OR (CBT‐BN) OR (cognitive behav*) OR (cognitive‐behav*) OR (self‐help)) AND ((random*) OR (RCT) OR (controlled) OR (allocate*) OR (assign*))”. The first search was completed on May 24th, 2023, and last updated on April 2nd, 2025. To identify relevant gray literature, we searched ProQuest, ClinicalTrials.gov, and the WHO International Clinical Trials Registry Platform Scholar using the search term described above. In addition, we screened the reference lists and database entries of relevant systematic reviews and meta‐analyses (Agras et al. 1989; Aman et al. 1997; Atwood and Friedman 2020; Beintner et al. 2014; Galsworthy‐Francis and Allan 2014; Linardon, Wade, et al. 2017; Pittock et al. 2018; Traviss et al. 2011) to identify additional relevant articles.

### 2.2 Inclusion Criteria

Only articles and theses written in English or German were considered. To be included in the meta‐analysis, they had to meet the following criteria: (1) be an RCT, (2) include adults (≥ 16 years) with an ED diagnosis (AN, BN, BED, other specified or unspecified ED) according to DSM or ICD criteria based on interviews or questionnaires, but not solely on self‐reported diagnoses, (3) compare CBT carried out as individual therapy or self‐help to (4) waitlist, active treatments that targeted ED psychopathology (i.e., psychosocial treatments that were not CBT such as other psychotherapies, nutritional counseling, or behavioral weight loss) or TAU carried out as individual therapy or self‐help, and (5) measure ED pathology using a valid, continuous instrument (see Schaefer et al. 2021), and/or measure the frequency of ED behaviors, that is, episodes of binge eating (for BN, BED, and mixed samples) or compensatory behavior (for BN), and/or measure BMI (only for AN). We excluded treatment studies that studied samples with EDs, but were not primarily focusing on reducing ED psychopathology, for example, studies targeting traits such as perfectionism, and studies including any form of pharmacotherapy.

### 2.3 Selection of Studies

All articles identified in the database search were combined, and duplicates were removed manually. Subsequently, identified abstracts were screened using the open‐source software ASReview (van de Schoot et al. 2021), which uses an active learning algorithm to sort the abstracts to be screened according to their expected relevance based on all previous screening decisions. Machine learning‐based screening often yields high recall rates while drastically reducing screening workload (Burgard and Bittermann 2023). After providing five pre‐selected relevant articles (Agras et al. 2000; Carrard et al. 2011; de Jong et al. 2020; Wagner et al. 2016; Zipfel et al. 2014) and five pre‐selected irrelevant articles (Aman et al. 1997; Liu et al. 2014; May et al. 2013; Schäfer et al. 2017; Sileo et al. 2020) abstracts were screened using ASReview until a stopping criterion was reached. We used a combination of a time‐based and a data‐driven stopping criterion because research has shown that such “mixed” stopping criteria perform well in terms of sensitivity and specificity (Campos et al. 2024). Specifically, after reviewing 124 articles, the screening was discontinued as soon as 1% of abstracts, that is, 25 abstracts, were marked as irrelevant in succession. This number was based on the number of included studies in a meta‐analysis with a strong commonality in inclusion criteria (Linardon, Wade, et al. 2017; Linardon, de la Piedad Garcia, and Brennan 2017). Following the abstract screening, the first author (JB) read the full texts of all articles previously marked as relevant to ultimately decide about their inclusion. When updating the literature search and during the ascendancy and descendancy approach, all abstracts and full texts were screened without using ASReview.

### 2.4 Data Extraction, Coding, and Quality Assessment

From those studies that were included, all relevant information was extracted twice: by the first author (JB) and independently by one of the other authors (MM, KJ). Disagreements were discussed and resolved. If a study included more than one comparison (e.g., CBT compared to TAU and CBT compared to psychodynamic treatment), all comparisons involving CBT were coded separately. Concerning ED pathology measures, we preferred global scores over subscales and integrated subscales when only those were available (see below). For binge‐eating frequency and frequency of compensatory behaviors, we preferred the number of episodes over the number of days the behavior occurred, and we preferred frequency per month over any other unit of time to comply with the most frequently used measure, the EDE(‐Q). In the case of missing or unclear information, we contacted the authors.

The following characteristics were coded: sample size, gender (proportions), age (mean, standard deviation, and range), race, and socioeconomic status (education, occupation) of participants; type of CBT (e.g., CBT‐BN, CBT‐E or CBT with additional modules); treatment format (therapist‐led, GSH, and pure SH); treatment duration (in weeks); type of comparison group (waitlist, active treatment, TAU); outcome type (ED pathology, binge‐eating frequency, frequency of compensatory behaviors, and BMI); outcome measure; and type of analysis (ITT and completer). We extracted means and standard deviations of all outcomes before and after treatment separately for each group. When allocating the studies to an ED diagnosis, atypical and subthreshold ED diagnoses were included in the overarching diagnosis (e.g., atypical AN was included as an AN sample). Only studies including patients with different diagnoses for which data were not available per diagnosis were classified as mixed.

Furthermore, to assess risk of bias, we used eight quality criteria suggested by Cuijpers et al. (2010) for the assessment of study quality in RCTs examining psychotherapy. Two authors (JB, KJ) conducted the risk of bias assessments independently. Afterwards, disagreements were discussed and resolved.

### 2.5 Statistical Analysis

All analyses were conducted in R (R Core Team 2025) using the *metafor* package (Viechtbauer 2010). We estimated multivariate multilevel meta‐analyses in which outcomes were nested in comparisons nested in studies using restricted maximum likelihood estimation. Multivariate meta‐analysis was chosen because it can be more efficient than univariate meta‐analysis, especially when correlations between outcomes are large and when some studies do not report all outcomes (Riley et al. 2017). Because all outcomes that were considered are among the main targets of CBT, we expected them to be highly correlated. In addition, most studies reported measures of ED pathology, but behavioral outcomes and BMI were less frequently reported, and, hence, multivariate meta‐analysis appeared suited for the data situation at hand. Sensitivity analyses were conducted to assess whether the multivariate approach yielded different results than conventional univariate meta‐analyses (see below). We computed between‐group standardized mean differences based on the post‐treatment outcomes with the pooled standard deviation as the denominator, and applied the small‐sample correction according to Hedges (1981). We set the correlations between effect sizes obtained for different outcomes within studies to *r* = 0.5, which mirrors our assumption that the different outcomes are targeted by CBT to a similar degree. The between‐study correlations were estimated. If any of these correlations were estimated at −1 or 1, which can indicate nonconvergence (Riley et al. 2007), we reestimated the model with a simpler structure for the variance–covariance matrix: specifically, we restricted the correlations to be equal (for models with three outcomes). If the correlation was still estimated at −1 or 1 (as well as for models with two outcomes), we set it to *ρ* = 0.5, and ran sensitivity analyses with different values of *ρ* to assess robustness. All analyses were performed separately for each ED and included the following outcomes: (1) ED pathology and BMI for AN, (2) ED pathology, binge‐eating frequency, and frequency of compensatory behaviors for BN, (3) ED pathology and binge‐eating frequency for BED, and (4) ED pathology and binge‐eating frequency for samples with mixed ED diagnoses. To ensure that all outcomes were coded in the same direction, that is, a negative effect indicating a better treatment outcome for CBT, effect sizes for BMI were multiplied by −1 before the analysis. We did not investigate compensatory behaviors for mixed diagnoses, as the mixed samples contained mostly BED cases.

In cases where only scores of subscales were reported for ED pathology, or when multiple equivalent measures of compensatory behaviors were available (e.g., Barakat et al. 2023), we aggregated them by averaging, as proposed by Borenstein (2009, chapter 24). For the subscales of the Eating Disorder Inventory (EDI; Garner et al. 1983) and the EDE‐Q, the correlations between the subscales were obtained from Garner et al. (1983) and a dataset available to the second author (MM), respectively. For the Barakat et al. (2023) study, we assumed a correlation of *r* = 0.5 between the measures.

To assess heterogeneity, we utilized the between‐study standard deviation (*τ*) as well as 95% prediction intervals (PI) as proposed by Riley et al. (2011) which correspond to the interval where 95% of the true effect sizes fall in the (hypothetical) population of studies. Both the between‐ and within‐study τ were used to calculate the PIs. To visualize the results, we created forest plots separately for each outcome and each ED diagnosis.

#### 2.5.1 Moderator Analyses

As potential moderators, we considered (1) the type of comparison group (waitlist vs. active treatment vs. TAU), (2) the format of CBT (therapist‐led vs. GSH vs. pure SH), (3) the duration of the CBT treatment (in weeks), (4) the ED baseline severity before treatment, measured as the weighted mean of pre‐treatment ED pathology in both conditions divided by the upper limit of the scale used to measure ED pathology, and (5) the year of publication. We chose to use weeks of treatment as the criterion (rather than, e.g., number of modules), as it was most consistently reported across studies with different treatment formats. To this end, we conducted meta‐regressions separately for each moderator and ED diagnosis, using the same multivariate multilevel model as for the respective main analysis. Moderator analyses were only conducted if information on the respective variable was available for a sufficient number of studies. For categorical moderators (type of comparison group and treatment format), at least three observations had to be available for two different levels. For continuous moderators (treatment duration, baseline severity, and publication year), we used scatterplots to assess whether there was sufficient variance in the moderator and whether a linear model seemed appropriate (see Supporting Information).

#### 2.5.2 Sensitivity Analyses

Finally, we performed several sensitivity analyses. Specifically, we repeated all analyses (1) using a univariate meta‐analytic model (which implies a between‐outcome‐correlation of *r* = 0 both within and between studies), (2) including only studies reporting ITT means and standard deviations, and (3) including only studies with a quality rating of 7 and above (range: 0–8). Furthermore, as noted above, we ran sensitivity analyses with different values of *ρ* if the between‐study correlation had to be fixed to achieve convergence.

#### 2.5.3 Publication Bias

To assess potential publication bias, we conducted multivariate Egger tests by including the standard error as a predictor in the multivariate meta‐analytic model, allowing for different weights per outcome to examine whether certain outcomes were more affected by publication bias than others. A negative weight indicates that larger standard errors (i.e., smaller sample sizes) are associated with larger negative effect sizes. As negative effect sizes suggest desirable lower levels of ED pathology, less binge eating, less compensatory behavior, and a higher BMI (for AN), this negative weight can be interpreted as an indicator for potential publication bias. In addition, we created funnel plots for visual assessment of potential publication bias.

## 3 Results

Code, data, and Supporting Information are available at https://osf.io/5t78f/.

### 3.1 Study Identification Process

**FIGURE 1:**
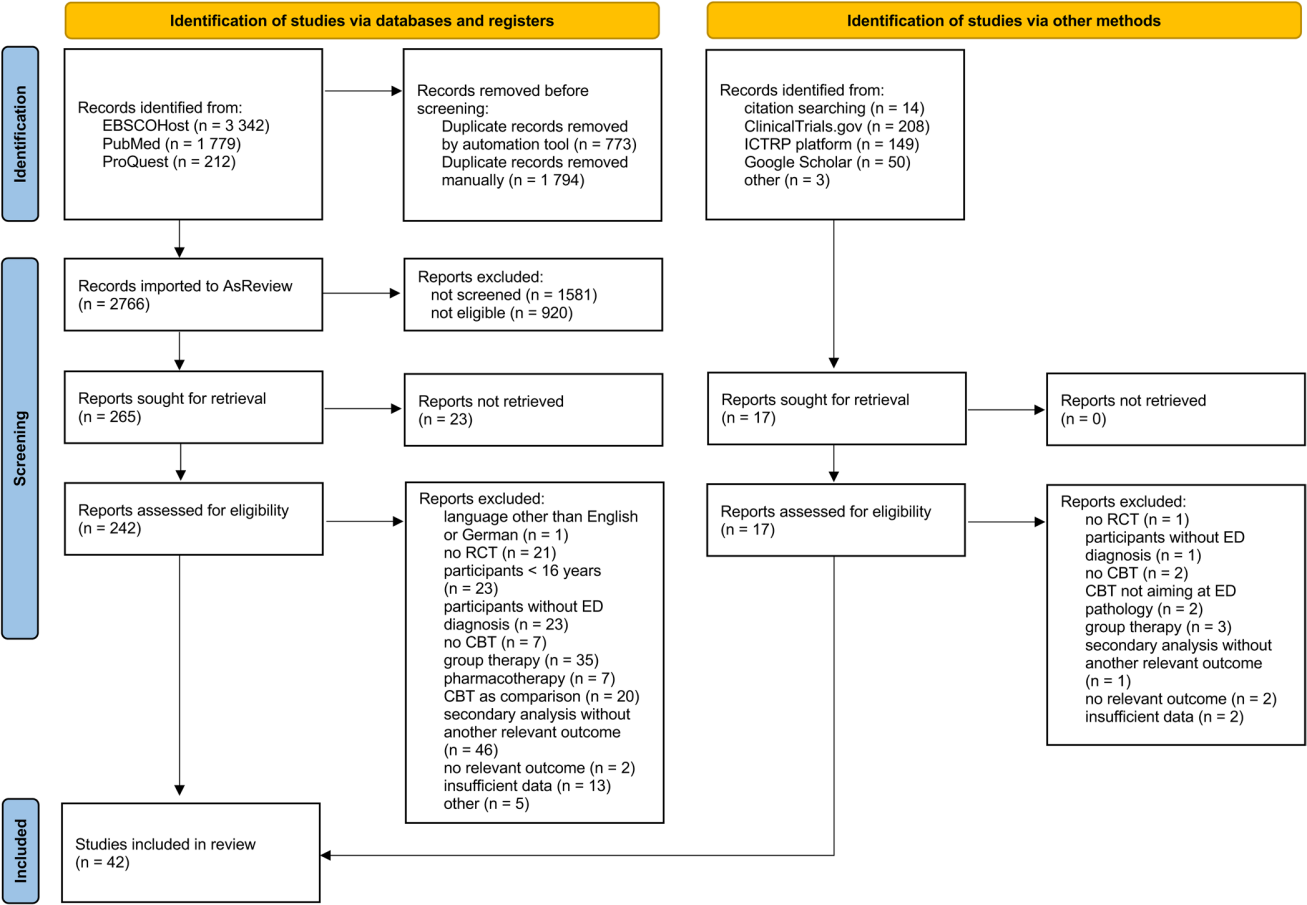
PRISMA flowchart. CBT = cognitive behavioral therapy; ED = eating disorder; RCT = randomized controlled trial.

Figure 1 depicts the study identification and selection process. The database search resulted in 5333 hits, out of which 2567 were duplicates. Abstract screening with ASReview was discontinued after checking 974 abstracts. Combined with 17 records identified through other sources, this led to a total of 259 full texts being assessed for eligibility. Of these, we included *k* = 42 studies (41 published peer‐review articles and 1 dissertation) with *c* = 56 comparisons in this meta‐analysis. The abstract of the article excluded due to language can be found in the Supporting Information.

### 3.2 Characteristics of Included Studies

Table 1 shows an overview of study characteristics by ED diagnosis. A detailed study overview for each ED diagnosis, including gender, age, race, and socioeconomic status, can be found in Table 2. All studies were conducted in North America, Europe, or Australia. Participants were predominantly female (> 90% on average), mostly White (> 70% in each sample), and rather educated (across the 19 studies that reported education, an average of 56.42% were college educated). Average mean sample ages ranged from 26.9 for BN studies to 38.2 for BED studies. The means of the average symptom scores at baseline ranged from 52.67% of the maximum score in AN studies to 63.81% in studies with mixed ED diagnoses.

**TABLE 1:** Overview of characteristics of included studies by eating disorder diagnosis.

	AN	BN	BED	Mixed
Sample				
Participants in treatment groups *N*	97	578	547	652
Participants in comparison groups *N*	166	593	670	675
Gender (% female) *M*	99.20	98.00	91.30	97.20
Mean age *M* (SD)	27.80 (4.98)	26.90 (3.47)	38.20 (5.52)	29.60 (6.60)
Baseline ED severity (% of maximum possible score) *M* (SD)	52.67 (3.77)	54.32 (14.63)	55.51 (8.47)	63.81 (7.73)
Treatment				
Format				
Therapist‐led *c* (*k*)	7 (4)	16 (11)		3 (2)
Guided self‐help *c* (*k*)		3 (3)	12 (9)	8 (7)
Pure self‐help *c* (*k*)		4 (4)	3 (3)	
Duration (in weeks) *M* (SD)	29.00 (8.87)	14.48 (4.66)	14.58 (5.09)	22.70 (16.81)
Type of comparison group				
Waitlist *c* (*k*)		9 (7)	8 (7)	5 (5)
Active treatment *c* (*k*)	7 (4)	13 (11)	5 (3)	4 (3)
Treatment as usual *c* (*k*)		1 (1)	2 (2)	2 (2)
Outcome				
Eating disorder pathology *c* (*k*)	7 (4)	23 (17)	15 (12)	11 (9)
Binge‐eating frequency *c* (*k*)		13 (10)	13 (10)	5 (4)
Frequency of compensatory behaviors *c* (*k*)		14 (9)		
BMI *c* (*k*)	5 (3)			
Type of analysis				
Intention‐to‐treat *c* (*k*)	7 (4)	7 (6)	9 (7)	5 (3)
Completer *c* (*k*)		16 (11)	6 (5)	6 (6)
Risk of bias				
Total score *M* (SD)	4.71 (2.14)	4.08 (1.83)	5.80 (1.66)	5.55 (1.13)
Low risk of bias (score ≥ 7) *c* (*k*)	3 (2)	3 (3)	6 (4)	2 (2)

Abbreviations: AN = anorexia nervosa; BED = binge‐eating disorder; BMI = body mass index; BN = bulimia nervosa; c = number of comparisons; k = number of studies; Mixed = samples with mixed eating disorder diagnoses.

**TABLE 2:** Characteristics of included studies by eating disorder diagnosis.

	Study	Country	Sample		Treatment	Comparison	Outcomes	Analysis	Risk of bias
Anorexia nervosa									
1	Byrne et al. (2017)	AUS	Gender: 95.8% female Age: *M* = 26.2, SD = 9.5 Race: NR Ethnicity: NR Nationality: 82.5% Australian, 7.5% UK, 4.2% South Africa Education: 43.6% tertiary education Occupation: NR Baseline severity: 55.33%	1	CBT‐E (therapist‐led, 40 weeks, 25/30/40 50‐min sessions) *N* = 39	MANTRA (therapist‐led, 40 weeks, 25/30/40 50‐min sessions) *N* = 41	EDE	ITT	7
				2	CBT‐E (therapist‐led, 40 weeks, 25/30/40 50‐min sessions) *N* = 39	SSCM (therapist‐led, 40 weeks, 25/30/40 50‐min sessions) *N* = 40	EDE	ITT	7
2	Channon et al. (1989)	GBR	Gender: 100% female age: *M* = 23.8 Race: NR Ethnicity: NR Education: NR Occupation: NR	3	CBT (therapist‐led, 24 weeks, 18 60‐min sessions) *N* = 8	BT (therapist‐led, 24 weeks, 18 60‐min sessions) *N* = 8	EDI Subscales BMI	ITT	3
				4	CBT (therapist‐led, 24 weeks, 18 60‐min sessions) *N* = 8	CT (therapist‐led, 24 weeks, 18 30‐min sessions) *N* = 8	EDI Subscales BMI	ITT	3
3	McIntosh et al. (2005)	NZL	Gender: 100% female Age: NR Race: NR Ethnicity: NR Education: NR Occupation: NR	5	CBT (therapist‐led, 20 weeks, 20 60‐min sessions) *N* = 19	IPT (therapist‐led, 20 weeks, 20 60‐min sessions) *N* = 21	EDE Subscales BMI	ITT	3
				6	CBT (therapist‐led, 20 weeks, 20 60‐min sessions) *N* = 19	NSSCM (therapist‐led, 20 weeks, 20 60‐min sessions) *N* = 16	EDE Subscales BMI	ITT	3
4	Touyz et al. (2013)	AUS GBR	Gender: 100% female Age: *M* = 33.4, SD = 9.6, 20–62 Race: NR Ethnicity: NR Education: 74.6% college degree Occupation: 12.2% full‐time employment Baseline severity: 50.00%	7	CBT (therapist‐led, 32 weeks, 30 sessions) *N* = 31	SSCM (therapist‐led, 32 weeks, 30 sessions) *N* = 32	EDE BMI	ITT	7
Bulimia nervosa									
1	Agras et al. (1989)	USA	Gender: 100% female Age: *M* = 29.2, SD = 8.6, 18–61 Race: NR Ethnicity: NR Education: 42% college graduates Occupation: 75% employed	1	CBT (therapist‐led, 16 weeks, 14 60‐min sessions) *N* = 17	Waitlist (16 weeks) *N* = 18	Aggregated Subscales Episodes of Self‐Induced Vomiting and Laxative Use	COM	3
				2	CBT (therapist‐led, 16 weeks, 14 60‐min sessions) *N* = 17	NDT (therapist‐led, 16 weeks, 14 60‐min sessions) *N* = 16	Aggregated Subscales Episodes of Self‐Induced Vomiting and Laxative Use	COM	3
				3	CBT + Response Prevention (therapist‐led, 16 weeks, 14 60‐min sessions) *N* = 16	waitlist (16 weeks) *N* = 18	Aggregated Subscales Episodes of Self‐Induced Vomiting and Laxative Use	COM	3
				4	CBT + Response Prevention (therapist‐led, 16 weeks, 14 60‐min sessions) *N* = 16	NDT (therapist‐led, 16 weeks, 14 60‐min sessions) *N* = 16	Aggregated Subscales Episodes of Self‐Induced Vomiting and Laxative Use	COM	3
2	Agras et al. (2000)	USA	Gender: 100% female Age: *M* = 28.1, SD = 7.2 Race: 77.0% White, 6.0% African American, 5.0% Asian, 1.0% American Indian Ethnicity: 11.0% Hispanic Education: NR Occupation: NR Baseline severity: 50.83%	5	CBT‐BN (therapist‐led, 20 weeks, 19 50‐min sessions) *N* = 65	IPT (therapist‐led, 20 weeks, 19 20‐min sessions) *N* = 64	EDE	COM	5
3	Banasiak et al. (2005)	AUS	Gender: 100% female age: *M* = 28.9 Race: NR Ethnicity: NR Education: 78.0% tertiary education Occupation: 61.5% in paid work Baseline severity: 64.67%	6	CBT (guided self‐help, 16 weeks, 9 20–30‐min sessions) *N* = 54	Waitlist (17 weeks) *N* = 55	EDE EDE Objective Binge Episodes	ITT	7
4	Barakat et al. (2023)	AUS	Gender: 96.3% female Age: *M* = 31.1, SD = 10.2, 16–65 Race: NR Ethnicity: NR Education: 86.2% more than 12 years of school attendance Occupation: 83.5% employed Baseline severity: 72.61%	7	CBT (guided self‐help, 12 weeks, 10 30‐min sessions) *N* = 37	Waitlist (10 weeks) *N* = 34	EDE‐Q EDE‐Q Objective Binges EDE‐Q Compensatory Behaviors	ITT	7
			Baseline severity: 69.12%	8	CBT (pure self‐help, 12 weeks) *N* = 37	Waitlist (10 weeks) *N* = 34	EDE‐Q EDE‐Q Objective Binges EDE‐Q Compen‐satory Behaviors	ITT	5
5	Carter et al. (2003)	CAN	Gender: 100% female Age: M = 27, SD = 8, 17–53 Race: 83% Caucasian, 7% Asian. 2% African Caribbean Ethnicity: NR Education: NR Occupation: NR Baseline severity: 72.25%	9	CBT (pure self‐help, 8 weeks) *N* = 28	Waitlist (8 weeks) *N* = 29	EDE Subscales	ITT	6
6	Cooper and Steere (1995)	GBR	Gender: 100% female Age: *M* = 23.8, 18–33 Race: NR Ethnicity: NR Education: NR Occupation: NR Baseline severity: 39.08%	10	CBT (therapist‐led, 18 weeks, 19 50‐min sessions) *N* = 13	BT (therapist‐led, 18 weeks, 19 50‐min sessions) *N* = 14	EAT Bulimic Episodes Frequency of Self‐Induced Vomiting	COM	4
7	Dicker (2003)	USA	Gender: 100% female Age: *M* = 20.1, SD = 1.9, 18–25 Race: 92% Caucasian, 4% Asian American Ethnicity: 4% Hispanic Education: NR Occupation: NR Baseline severity: 66.67%	11	AF‐CBT (therapist‐led, 8 weeks, 8 60‐min sessions) *N* = 13	Waitlist (8 weeks) *N* = 13	EDE‐Q	ITT	3
8	Fairburn et al. (1986)	GBR	Gender: 100% female Age: *M* = 22.9, SD = 4.4 Race: NR Ethnicity: NR Education: NR Occupation: NR Baseline severity: 37.79%	12	CBT‐BN (therapist‐led, 18 weeks, 19 sessions) *N* = 11	FP (therapist‐led, 18 weeks, 19 sessions) *N* = 11	EAT	COM	3
9	Fairburn et al. (1991)	GBR	Gender: 100% female Age: *M* = 24.2., SD = 6.2 Race: NR Ethnicity: NR Education: NR Occupation: NR Baseline severity: 39.83%	13	CBT‐BN (therapist‐led, 18 weeks, 19 40–50‐min sessions) *N* = 21	BT (therapist‐led, 18 weeks, 19 40–50‐min sessions) *N* = 18	EAT Objective Bulimic Episodes	COM	3
			baseline severity: 38.13%	14	CBT‐BN (therapist‐led, 18 weeks, 19 40–50‐min sessions) *N* = 21	IPT (therapist‐led, 18 weeks, 19 40–50‐min sessions) *N* = 21	EAT Objective Bulimic Episodes	COM	3
10	Garner et al. (1993)	USA	Gender: 100% female Age: NR Race: NR Ethnicity: NR Education: NR Occupation: NR Baseline severity: 28.29%	15	CBT‐BN (therapist‐led, 18 weeks, 19 45–60‐min sessions) *N* = 25	SET (therapist‐led, 18 weeks, 19 45–60‐min sessions) *N* = 25	EAT Frequency of Binge Eating Frequency of Self‐Induced Vomiting	COM	4
11	Griffiths et al. (1994)	AUS	Gender: 100% female Age: *M* = 25.9, SD = 5.7 Race: NR Ethnicity: NR Education: NR Occupation: 64.1% employed, 11.5% students Baseline severity: 41.77%	16	CBT (therapist‐led, 8 weeks, 7 50–60‐min sessions) *N* = 19	Waitlist (8 weeks) *N* = 22	EAT Frequency of Binge Eating Frequency of Purges	COM	1
			Baseline severity: 39.30%	17	CBT (therapist‐led, 8 weeks, 7 50–60‐min sessions) *N* = 19	HBT (therapist‐led, 8 weeks, 7 50–60‐min sessions) *N* = 21	EAT Frequency of Binge Eating Frequency of Purges	COM	1
12	Hartmann et al. (2024)	GER	Gender: 96.8% female Age: *M* = 29.6, SD = 8.6 Race: NR Ethnicity: NR Nationality: 96.1% German Education: 51.3% university degree Occupation: 50.0% in occupation	18	CBT (pure self‐help, 16 weeks) *N* = 60	TAU (16 weeks) *N* = 66	EDE‐Q EDE‐Q Binge Eating Episodes EDE‐Q Compensa‐tory Behaviors	COM	5
13	Juarascio et al. (2021)	USA	Gender: 88.6% female Age: *M* = 33.2, 18–60 Race: 84.1% White, 9.1% Black, 6.8% Asian Ethnicity: 13.6% Hispanic Education: NR Occupation: NR Baseline severity: 55.14%	19	CBT‐Ef (therapist‐led, 20 weeks, 20 sessions) *N* = 18	MABT (therapist‐led, 20 weeks, 20 sessions) *N* = 26	EDE EDE Objective Binges	ITT	3
14	Poulsen et al. (2014)	DEN	Gender: 96.8% female Age: *M* = 25.8, SD = 4.9 Race: NR Ethnicity: NR Education: NR Occupation: NR Baseline severity: 63.27%	20	CBT‐Ef (therapist‐led, 20 weeks, 20 50‐min sessions) *N* = 33	PT (therapist‐led, 104 weeks, 104 50‐min sessions) *N* = 24	EDE EDE Objective Binges Purge Frequency	COM	6
15	Schmidt et al. (2008)	GBR	Gender: 95.9% female Age: *M* = 27.1 Race: 73.3% White British Ethnicity: NR Education: NR Occupation: NR Baseline severity: 56.43%	21	CBT (pure self‐help, 12 weeks) *N* = 35	Waitlist (12 weeks) *N* = 39	EDE	COM	5
16	Steele and Wade (2008)	AUS	Gender: 97.9% female Age: *M* = 26.0, 17–39 Race: NR Ethnicity: NR Education: 24.2% tertiary education Occupation: 63.6% in paid work Baseline severity: 71.75%	22	CBT (guided self‐help, 6 weeks, 8 40‐min sessions) *N* = 12	MBCT (guided self‐help, 6 weeks, 8 40‐min sessions) *N* = 11	EDE	COM	3
17	Wonderlich et al. (2014)	USA	Gender: 90.0% female Age: *M* = 27.3, SD = 9.6 Race: 87.5% White, 6.3% Asian, 1.3% African American, 1.3% Native American Ethnicity: 2.5% Hispanic Education: 45.0% college degree Occupation: NR Baseline severity: 54.17%	23	CBT‐Ef (therapist‐led, 19 weeks, 21 50‐min sessions) *N* = 40	ICAT (therapist‐led, 19 weeks, 21 50‐min sessions) *N* = 40	EDE Objective Binge Episodes Purging Episodes	ITT	8
Binge‐eating disorder									
1	Cachelin et al. (2019)	USA	Gender: 100% female Age: *M* = 27.0, SD = 8.9 Race: NR Ethnicity: 100% Latinas SES: 37.4 Hollinghead index Baseline severity: 64.86%	1	CBT‐BN (guided self‐help, 12 weeks, 8 25‐min sessions) *N* = 21	Waitlist (12 weeks) *N* = 19	EDE Subscales EDE Objective Binge Episodes	ITT	5
2	Carrard et al. (2011)	CHE	Gender: 100% female Age: *M* = 36.0, SD = 11.4, 21–60 Race: NR Ethnicity: NR Education: 48.6% university education Occupation: 75.7% employed Baseline severity: 57.50%	2	CBT (guided self‐help, 24 weeks) *N* = 37	Waitlist (24 weeks) *N* = 37	EDE‐Q	ITT	5
3	de Bar et al. (2011)	USA	Gender: 100% female Age: *M* = 39.1, SD = 6.7 Race: 91.0% White Ethnicity: 4% Hispanic Education: NR Occupation: NR Baseline severity: 68.55%	3	CBT‐BN (guided self‐help, 12 weeks, 8 20–25‐min sessions) *N* = 73	TAU (12 weeks) *N* = 76	EDE‐Q Subscales	COM	3
4	Grilo and Masheb (2005)	USA	Gender: 79.0% female Age: *M* = 46.3, SD = 9.0, 20–60 Race: 77.0% Caucasian, 10.0% African American Ethnicity: 11.0% Hispanic Education: 87% college education Occupation: NR Baseline severity: 61.88%	4	CBT‐BN (guided self‐help, 12 weeks, 6 15–20‐min sessions) *N* = 37	BWL (guided self‐help, 12 weeks, 6 15–20‐min sessions) *N* = 38	EDE‐Q Subscales EDE‐Q Objective Binge Episodes	ITT	8
			Baseline severity: 59.57%	5	CBT‐BN (guided self‐help, 12 weeks, 6 15–20‐min sessions) *N* = 37	CT (guided self‐help, 12 weeks, 6 15–20‐min sessions) *N* = 15	EDE‐Q Subscales EDE‐Q Objective Binge Episodes	ITT	8
5	Grilo et al. (2013)	USA	Gender: 79.2% female Age: *M* = 45.8, SD = 11.0 Race: 45.8% White, 35.4% African American Ethnicity: 6.3% Hispanic Education: 48.9% college degree Occupation: NR Baseline severity: 41.25%	6	CBT‐BN (pure self‐help, 16 weeks) *N* = 24	TAU (16 weeks) *N* = 24	EDE Subscales EDE Objective Binge Episodes	ITT	5
6	Melisse et al. (2023)	NLD	Gender: 90.6% female Age: *M* = 39.4, SD = 13.1 Race: NR Ethnicity: NR Education: 21.1% university education Occupation: 66.7% employed, 10.6% student Baseline severity: 53.33%	7	CBT‐E (guided self‐help, 12 weeks, 12 20‐min sessions) *N* = 90	Waitlist (12 weeks) *N* = 90	EDE Objective Binges	ITT	7
7	Peterson et al. (2020)	USA	Gender: 85.7% female Age: *M* = 39.7, SD = 13.4 Race: 91.1% Caucasian Ethnicity: NR Education: 68.8% college degree Occupation: NR Baseline severity: 44.74%	8	CBT‐E (guided self‐help, 17 weeks, 10 30‐min sessions) *N* = 39	ICAT (therapist‐led, 17 weeks, 21 50‐min sessions) *N* = 45	EDE	COM	6
8	Pruessner et al. (2024)	GER	Gender: 96.1% female Age: *M* = 35.9, SD = 10.6 Race: NR Ethnicity: NR Education: 52.3% university degree Occupation: 80.5% employed, 13.0% student Baseline severity: 59.83%	9	CBT (pure self‐help, 12 weeks) *N* = 60	Waitlist (12 weeks) *N* = 67	EDE‐Q EDE‐Q Objective Binge Episodes	COM	5
9	Shapiro et al. (2007)	USA	Gender: 93.2% female Age: NR Race: 46.9% White, 16.6% Black, 2.9% Asian Ethnicity: NR Education: NR Occupation: NR Baseline severity: 61.16%	10	CBT (pure self‐help, 10 weeks) *N* = 15	Waitlist (10 weeks) *N* = 20	BES Binge Days	COM	3
10	Wagner et al. (2016)	GER	Gender: 96.4% female Age: *M* = 35.1, SD = 9.9, 18–61 Race: NR Ethnicity: NR Education: 71.7% high education Occupation: NR Baseline severity: 65.00%	11	CBT (guided self‐help, 16 weeks) *N* = 69	Waitlist (16 weeks) *N* = 70	EDE‐Q EDE‐Q Objective Binge Episodes	ITT	8
11	Wilson et al. (2010)	USA	Gender: NR Age: 19–77 Race: 79.3% White, 14.2% Black, 0.5% American Indian Ethnicity: 5.9% Hispanic Education: 34% college degree Occupation: NR Baseline severity: 45.87%	12	CBT‐BN (guided self‐help, 24 weeks, 10 25‐min sessions) *N* = 66	IPT (therapist‐led, 24 weeks, 19 50–60‐min sessions) *N* = 75	EDE Binge Days	ITT	7
			Baseline severity: 45.82%	13	CBT‐BN (guided self‐help, 24 weeks, 10 25‐min sessions) *N* = 66	BWL (therapist‐led, 24 weeks, 19 50–60‐min sessions) *N* = 64	EDE Binge Days	ITT	7
Mixed ED diagnoses									
1	Fairburn et al. (2015)	GBR	Gender: 97.7% female Age: *M* = 25.9, SD = 7.7 Race: 95.4% White, 0.8% Black British, 0.8% Asian Chinese, 0.8% Asian British, 2.3% mixed Ethnicity: NR Education: NR Occupation: 12.3% higher occupational social class, 16.2% lower occupational class, 50.0% students Baseline severity: 59.25%	1	CBT‐Ef (therapist‐led, 20 weeks, 20 50‐min sessions) *N* = 60	IPT (therapist‐led, 20 weeks, 20 50‐min sessions) *N* = 58	EDE	COM	6
2	Fitzsimmons‐Craft et al. (2020)	USA	Gender: 100% female Age: *M* = 22.1, SD = 4.9 Race: 60.0% White, 17.1% Asian, 5.4% Black, 0.4% American Indian/Alaskan Native, 0.1% Native Hawaiian, 7.7% multiracial Ethnicity: 17.4% Hispanic Education: NR Occupation: 89.0% students Baseline severity: 59.82%	2	CBT (guided self‐help, 32 weeks) *N* = 227	TAU (32 weeks) *N* = 243	EDE‐Q Binges Purges	COM	6
3	Ljotsson et al. (2007)	SWE	Gender: 94.2% female Age: *M* = 34.4 Race: NR Ethnicity: NR Education: NR Occupation: NR Baseline severity: 62.43%	3	CBT‐BN (guided self‐help, 12 weeks) *N* = 24	Waitlist (12 weeks) *N* = 34	EDE‐Q EDE‐Q Objective Bulimic Episodes	COM	6
4	McIntosh et al. (2016)	NZL	Gender: 100% female Age: NR Race: NR Ethnicity: NR Education: *M* = 15.4 years Occupation: 11% unemployed Baseline severity: 59.17%	4	CBT‐E (therapist‐led, 52 weeks, 39 sessions) *N* = 38	ST (therapist‐led, 52 weeks, 39 sessions) *N* = 38	EDE Objective Binge Eating	ITT	6
			Baseline severity: 59.19%	5	AF‐CBT (therapist‐led, 52 weeks, 39 sessions) *N* = 36	ST (therapist‐led, 52 weeks, 39 sessions) *N* = 38	EDE Objective Binge Eating	ITT	6
5	Robinson and Serfaty (2008)	GBR	Gender: NR Age: *M* = 24.5, SD = 7.29 Race: NR Ethnicity: NR Education: NR Occupation: NR Baseline severity: 79.26%	6	CBT (guided self‐help, 12 weeks) *N* = 36	Waitlist (12 weeks) *N* = 27	BITE	ITT	4
			Baseline severity: 78.98%	7	CBT (guided self‐help, 12 weeks) *N* = 36	SDW (pure self‐help, 12 weeks) *N* = 34	BITE	ITT	4
6	Sánchez‐Ortís et al. (2011)	GBR	Gender: NR Age: *M* = 23.9, SD = 5.9 Race: NR Ethnicity: NR Nationality: 59% British Education: NR Occupation: NR Baseline severity: 60.00%	8	CBT (guided self‐help, 12 weeks, 8 45‐min sessions) *N* = 36	Waitlist (12 weeks) *N* = 31	EDE Objective Binges	COM	4
7	Strandskov et al. (2017)	SWE	Gender: 96.7% female Age: *M* = 29.1, SD = 6.7, 18–64 Race: NR Ethnicity: NR Education: 47.6% university education Occupation: NR Baseline severity: 62.50%	9	CBT (guided self‐help, 8 weeks) *N* = 46	Waitlist (8 weeks) *N* = 46	EDE‐Q	ITT	7
8	Striegel‐Moore et al. (2010)	USA	Gender: 91.9% female Age: *M* = 37.2, SD = 7.8 Race: 96.7% White Ethnicity: 3.6% Hispanic Education: 82.1% college education Occupation: NR Baseline severity: 63.00%	10	CBT‐BN (guided self‐help, 12 weeks, 8 20–25‐min sessions) *N* = 52	TAU (12 weeks) *N* = 60	EDE‐Q Subscales	COM	7
9	ter Huurne et al. (2015)	NLD	Gender: 100% female Age: *M* = 39.4, SD = 11.6 Race: NR Ethnicity: NR Education: 50.9% highly educated Occupation: 79.0% employed Baseline severity: 58.33%	11	CBT (guided self‐help, 15 weeks) *N* = 97	Waitlist (15 weeks) *N* = 104	EDE‐Q	COM	5
12	Wyssen et al. (2021)	GER	Gender: 87.3% female Age: *M* = 37.2, SD = 10.4 Race: NR Ethnicity: NR Nationality: 88.9% Swiss, 95% German, 1.6% Austrian Education: 48% university degree Occupation: NR Baseline severity: 50.24%	14	CBT (guided self‐help, 8 weeks, 8 sessions) *N* = 16	Waitlist (4 weeks) *N* = 15	EDE‐Q EDE‐Q Binges	COM	5
			Baseline severity: 52.95%	15	CBT (guided self‐help, 8 weeks, 8 sessions) *N* = 16	Waitlist (4 weeks) *N* = 15	EDE‐Q EDE‐Q Binges	COM	5

Abbreviations: Country: AUS = Australia; CAN = Canada; CHE = Switzerland; DEN = Denmark; GBR = Great Britain; GER = Germany; NDL = Netherlands; NZL = New Zealand; SWE = Sweden; USA = United States of America. Sample: NR = not reported. Treatment: CBT = cognitive‐behavioral therapy; CBT‐BN = cognitive‐behavioral therapy for bulimia nervosa (Fairburn 1995; Fairburn et al. 1993); CBT‐E = enhanced cognitive‐behavioral therapy for eating disorders (Fairburn 2015); CBT‐Ef = enhanced cognitive‐behavioral therapy for eating disorders, focused version (Fairburn 2015), AF‐CBT = appetite‐focused CBT. Comparison: BT = behavioral therapy; BWL = behavioral weight loss; CT = control therapy; FP = focal psychotherapy; HBT = hypno‐behavioral therapy; ICAT = integrative cognitive‐affective therapy; IPT = interpersonal psychotherapy, MABT = mindfulness and acceptance‐based treatment; MBCT = mindfulness‐based cognitive therapy; MANTRA = Maudsley Anorexia Nervosa Treatment for Adult; NDT = non‐directive therapy; NSSCM = nonspecific supportive clinical management; PT = psychoanalytic therapy; SDW = self‐directed writing; SET = supportive‐expressive therapy; SSCM = Specialist Supportive Clinical Management; ST = schema therapy; TAU = treatment as usual. Outcomes: BITE = Bulimic Investigatory Test Edinburgh; BMI = body mass index; EAT = Eating Attitudes Test; EDE = Eating Disorder Examination; EDE‐Q = Eating Disorder Examination Questionnaire; EDI = Eating Disorder Inventory. Analysis: COM = completer; ITT = intention‐to‐treat.

We included *c* = 7 comparisons reported in *k* = 4 studies investigating AN. All of these studies compared therapist‐led CBT to an active treatment condition. On average, CBT for AN lasted 29 weeks. BN was studied in *c* = 23 comparisons in *k* = 17 studies. Most comparisons examined therapist‐led CBT (70%); only a few comparisons investigated GSH (13%) or pure SH CBT (17%). The most frequent type of comparison group was other active treatments (57%). The average treatment length was about 15 weeks. Concerning BED, *c* = 15 comparisons reported in *k* = 12 studies were included. Most of these comparisons concerned GSH CBT (80%); the others studied pure SH. About 53% of comparisons compared CBT to waitlist. CBT treatment lasted about 15 weeks on average. Finally, we included *c* = 11 comparisons in *k* = 9 studies that investigated samples with mixed ED diagnoses. GSH was the most common treatment format, with about 73%; the remaining studies examined therapist‐led CBT. Forty‐five percent of studies examined waitlist controls, 36% examined active treatment, and 19% examined TAU comparison groups, respectively. The average CBT treatment duration was about 23 weeks. Overall, study quality was poor: only *k* = 2 studies with *c* = 3 comparisons for AN, *k* = 3 studies with *c* = 3 comparisons for BN, *k* = 4 studies with *c* = 6 comparisons for BED, and *k* = 2 studies with *c* = 3 comparisons for mixed EDs had a study quality score of 7 or above, indicating low risk of bias. Most studies did not include information about socioeconomic status, and race and ethnicity were inconsistently reported.

In the following, we describe the most important meta‐analytic results for each ED diagnosis. Full results are shown in Table 3. Separate forest plots per diagnosis depicting the results for each of the relevant outcomes as well as results of the sensitivity analyses are available in the Supporting Information.

**TABLE 3:** Results of the moderator analyses.

Type of comparison group	Bulimia nervosa			Binge‐eating disorder			Mixed eating disorder diagnoses		
	*g*	95% CI	95% PI	*g*	95% CI	95% PI	*g*	95% CI	95% PI
Waitlist									
ED pathology	−0.78	[−1.16; −0.40]	[−1.92; 0.36]	−0.61	[−0.99; −0.22]	[−1.57; 0.36]	−0.76	[−1.07; −0.46]	[−1.42; −0.11]
BE frequency	−0.59	[−0.84; −0.35]	[−0.84; −0.34]	−0.60	[−0.95; −0.25]	[−1.48; 0.27]	−0.40	[−0.75; −0.06]	[−0.75; −0.06]
CB frequency	−0.66	[−0.94; −0.38]	[−0.96; −0.36]						
Active treatment									
ED pathology	−0.20	[−0.53; 0.14]	[−1.32; 0.93]	−0.06	[−0.61; 0.49]	[−1.11; 0.98]	−0.26	[−0.62; 0.09]	[−0.95; 0.42]
BE frequency	−0.15	[−0.36; 0.05]	[−0.36; 0.06]	−0.24	[−0.83; 0.35]	[−1.23; 0.75]	0.12	[−0.26; 0.50]	[−0.26; 0.50]
CB frequency	−0.14	[−0.35; 0.07]	[−0.38; 0.10]						
Treatment as usual									
ED pathology	−0.28	[−1.42; 0.85]	[−1.85; 1.28]	−0.74	[−1.45; −0.04]	[−1.88; 0.39]	−0.47	[−0.93; −0.01]	[−1.21; 0.27]
BE frequency	−0.46	[−0.81; −0.10]	[−0.82; −0.10]	−0.41	[−1.34; 0.51]	[−1.64; 0.81]	−0.17	[−0.35; 0.01]	[−0.35; 0.02]
CB frequency	−0.37	[−0.74; 0.002]	[−0.75; 0.02]						
Treatment format	*g*	95% CI	95%‐PI	*g*	95%‐CI	95%‐PI	*g*	95% CI	95%‐PI
Therapist‐led									
ED pathology	−0.45	[−0.79; −0.11]	[−1.46; 0.55]				−0.39	[−0.86; 0.08]	[−1.11; 0.33]
BE frequency	−0.21	[−0.41; −0.01]	[−0.42; −0.00]				0.07	[−0.36; 0.51]	[−0.44; 0.59]
CB frequency	−0.24	[−0.45; −0.04]	[−0.54; 0.05]						
Guided self‐help									
ED pathology	−0.57	[−1.09; −0.06]	[−1.65; 0.50]	−0.55	[−0.90; −0.20]	[−1.57; 0.46]	−0.58	[−0.83; −0.32]	[−1.19; 0.03]
BE frequency	−0.65	[−0.94; −0.36]	[−0.94; −0.36]	−0.58	[−0.91; −0.24]	[−1.44; 0.29]	−0.25	[−0.50; −0.01]	[−0.62; 0.12]
CB frequency	−0.22	[−0.63; 0.20]	[−0.68; 0.25]						
Pure self‐help									
ED pathology	−0.18	[−0.65; 0.29]	[−1.24; 0.88]	−0.24	[−0.87; 0.39]	[−1.38; 0.90]			
BE frequency	−0.40	[−0.67; −0.13]	[−0.67; −0.13]	−0.26	[−0.81; 0.29]	[−1.23; 0.71]			
CB frequency	−0.42	[−0.73; −0.12]	[−0.79; −0.06]						
Treatment duration	*b*	*z*	*p*	*b*	*z*	*p*	*b*	*z*	*p*
ED pathology	0.01	0.47	0.641	0.02	0.48	0.631	0.01	2.97	0.023
BE frequency	0.03	1.63	0.103	0.02	0.62	0.536			
CB frequency	0.04	1.87	0.061						
Baseline severity	*b*	*z*	*p*	*b*	*z*	*p*	*b*	*z*	*p*
ED pathology	0.004	0.45	0.651	−0.04	−2.97	0.003	0.01	0.37	0.713
BE frequency	−0.01	−1.22	0.223	−0.04	−2.68	0.007	−0.14	−1.43	0.153
CB frequency	0.003	0.46	0.647						
Publication year	*b*	*z*	*p*	*b*	*z*	*p*	*b*	*z*	*p*
ED pathology	0.02	1.68	0.094	−0.01	−0.39	0.697	0.04	2.87	0.004
BE frequency	−0.002	−0.38	0.707	−0.01	−0.63	0.526			
CB frequency	0.004	0.66	0.506						

Abbreviations: BE = binge eating; CB = compensatory behaviors; ED = eating disorder.

### 3.3 Meta‐Analytic Results

#### 3.3.1 Anorexia Nervosa Studies

In comparison to other active treatments, therapist‐led CBT for AN was not associated with lower levels of ED pathology, *g* = −0.06, 95% CI = [−0.38; 0.27]. Descriptively, BMI was lower following CBT compared to active treatment, but not significantly so, *g* = 0.15, 95% CI = [−0.18; 0.48]. For ED pathology, there was moderate heterogeneity, *τ* = 0.19, 95%‐PI = [−0.55; 0.43]. Heterogeneity for BMI was small, *τ* = 0.04, 95% PI = [−0.19; 0.49]. These results remained consistent in the sensitivity analyses (see Supporting Information). Due to the small number of studies, no moderator analyses were conducted.

#### 3.3.2 Bulimia Nervosa Studies

Across all types of comparison groups and treatment formats, there was an effect in favor of CBT for ED pathology, *g* = −0.42, 95% CI = [−0.68; −0.16], binge‐eating frequency, *g* = −0.35, 95% CI = [−0.50; −0.19], and frequency of compensatory behaviors, *g* = −0.31, 95% CI = [−0.47; −0.15] post‐treatment. Of note, most studies reported purging behaviors as outcomes, but aggregated frequencies often entailed non‐purging behaviors such as driven exercise (e.g., Juarascio et al. 2021) and Barakat et al. (2023) reported driven exercise separately. There was large heterogeneity for ED pathology, *τ* = 0.47, 95% PI = [−1.38; 0.55], and small heterogeneity for binge‐eating frequency, *τ* = 0.07, 95% PI = [−0.55; −0.14], and frequency of compensatory behaviors, *τ* = 0.08, 95% PI = [−0.53; −0.09]. The effect for frequency of compensatory behaviors was not significant in the univariate analyses. When only including ITT comparisons, only the effect for binge‐eating frequency remained significant. In addition, the effects for ED pathology and binge‐eating frequency, but not for frequency of compensatory behaviors, were significant when excluding low‐quality studies. Of note, all comparisons comparing therapist‐led CBT for BN against waitlist comparison groups were excluded in the ITT and study quality sensitivity analyses, as they did not use ITT analyses and had low quality (see Supporting Information).

##### 3.3.2.1 Type of Comparison Group

Type of comparison group emerged as a significant moderator for ED pathology, *Q*(2) = 9.56, *p* = 0.008, binge‐eating frequency, *Q*(2) = 8.47, *p* = 0.015, and frequency of compensatory behaviors, *Q*(2) = 10.29, *p* = 0.006. When compared to waitlist, CBT for BN was associated with lower levels of ED pathology, less binge eating, and less compensatory behaviors (see Table 2). There were no significant differences between CBT and other active treatments for either outcome. In comparison to TAU, CBT for BN was associated with lower binge‐eating frequency, but there were no differences for ED pathology or frequency of compensatory behaviors. Residual heterogeneity was large for ED pathology and small for both binge eating and the frequency of compensatory behaviors (see Table 2). These results remained mostly consistent for different values for ρ. The only exception was an effect in favor of CBT compared to TAU for frequency of compensatory behaviors that was significant when *ρ* ≤ 0.3 (see Supporting Information).

##### 3.3.2.2 Treatment Format

Treatment format did not moderate the magnitude of the effect for ED pathology, *Q*(2) = 3.36, *p* = 0.186, binge‐eating frequency, *Q*(2) = 5.93, *p* = 0.051, or frequency of compensatory behaviors, *Q*(2) = 1.43, *p* = 0.489. After therapist‐led CBT for BN, participants showed lower levels of ED pathology, a lower binge‐eating frequency, and a lower frequency of compensatory behaviors compared to participants in any type of comparison group (see Table 2). GSH for BN was associated with less ED pathology and a lower binge‐eating frequency compared to comparison groups, but there were no differences for frequency of compensatory behaviors. There was an effect in favor of pure SH CBT for binge‐eating frequency and frequency of compensatory behaviors in comparison groups, but not for ED pathology. Residual heterogeneity was high for ED pathology, small for binge‐eating frequency, and moderate for frequency of compensatory behaviors (see Table 2). These results remained consistent for different values for *ρ* (see Supporting Information).

##### 3.3.2.3 Treatment Duration

Treatment duration was not a significant moderator (see Table 2). These results remained consistent for different values of *ρ* (see Supporting Information).

##### 3.3.2.4 Baseline Eating Disorder Severity

Baseline ED severity did not moderate the magnitude of the effect for any outcome (see Table 2). This was consistent in the sensitivity analyses (see Supporting Information).

##### 3.3.2.5 Publication Year

The magnitude of effect sizes was not moderated by publication year for any outcome (*see* Table 2). This was consistent in the sensitivity analyses (see Supporting Information).

#### 3.3.3 Binge‐Eating Disorder Studies

CBT for BED was associated with lower levels of ED pathology, *g* = −0.48, 95% CI = [−0.78; −0.18], and a lower binge‐eating frequency, *g* = −0.50, 95% CI = [−0.77; −0.22], across all types of comparison groups and treatment formats. Heterogeneity was large for both ED pathology, *τ* = 0.45, 95% PI = [−1.45; 0.49], and binge‐eating frequency, *τ* = 0.51, 95% PI = [−1.31; 0.32]. The results were consistent in the sensitivity analyses (see Supporting Information).

##### 3.3.3.1 Type of Comparison Group

Type of comparison group did not moderate the magnitude of the effect for ED pathology, *Q*(2) = 3.16, *p* = 0.206, or binge‐eating frequency, *Q*(2) = 1.10, *p* = 0.576, that is, effect sizes did not differ between types of comparison groups. There was an effect in favor of CBT for both ED pathology and binge‐eating frequency when compared to waitlist (see Table 2). We found no differences for either outcome between CBT and active treatments. In comparison to TAU, CBT was associated with less ED pathology, but not with less binge eating. Residual heterogeneity was high for both ED pathology and binge‐eating frequency (see Table 2).

##### 3.3.3.2 Treatment Format

Treatment format did not emerge as a moderator for ED pathology, *Q*(1) = 0.72, *p* = 0.397, or binge‐eating frequency, *Q*(1) = 0.92, *p* = 0.336, that is, effect sizes did not differ between treatment formats. Results showed an effect in favor of GSH CBT compared to comparison groups for both ED pathology and binge‐eating frequency (see Table 2). Pure SH CBT was not associated with lower levels of ED pathology or less binge eating in contrast to comparison groups. There was high residual heterogeneity for both ED pathology and binge‐eating frequency (see Table 2).

##### 3.3.3.3 Treatment Duration

Treatment duration did not moderate the magnitude of the effect for ED pathology or binge‐eating frequency (see Table 2).

##### 3.3.3.4 Baseline Eating Disorder Severity

The magnitude of the effects for ED pathology and binge‐eating frequency were moderated by baseline ED severity. For BED, the positive effect of CBT was larger in studies with higher levels of baseline ED severity (see Table 2). These results remained consistent in the sensitivity analyses (see Supporting Information).

##### 3.3.3.5 Publication Year

Publication year was not a significant moderator for any outcome (see Table 2).

#### 3.3.4 Mixed Eating Disorders Studies

In samples with mixed ED diagnoses, there was an effect in favor of CBT for ED pathology, *g* = −0.53, 95% CI = [−0.73; −0.33]; but not for binge‐eating frequency, *g* = −0.18, 95% CI = [−0.39; 0.04]. There was large heterogeneity for ED pathology, *τ* = 0.22, 95% PI = [−1.04; −0.02], and moderate heterogeneity for binge‐eating frequency, *τ* = 0.10, 95% PI = [−0.53; 0.18]. These results remained consistent in the sensitivity analyses (see Supporting Information).

##### 3.3.4.1 Type of Comparison Group

Type of comparison group moderated the magnitude of the effect for ED pathology, *Q*(2) = 6.80, *p* = 0.033, but not for binge‐eating frequency, *Q*(2) = 4.10, *p* = 0.129. Participants with mixed ED diagnoses had lower levels of ED pathology and a lower binge‐eating frequency after CBT treatment compared to participants in waitlist comparison groups (see Table 2). CBT was neither associated with lower levels of ED pathology nor less binge eating in comparison to other active treatments. When compared with TAU, CBT was associated with lower levels of ED pathology, but not less binge eating. For ED pathology, heterogeneity was large, and there was small heterogeneity for binge‐eating frequency (see Table 2). These results remained consistent in the sensitivity analyses (see Supporting Information).

##### 3.3.4.2 Treatment Format

Treatment format did not emerge as a significant moderator for ED pathology, *Q*(1) = 0.48, *p* = 0.488, or frequency of binge eating, *Q*(1) = 1.65, *p* = 0.120; that is, effect sizes did not differ between treatment formats. In samples with mixed ED diagnoses, therapist‐led CBT was neither associated with lower levels of ED pathology nor with a lower binge‐eating frequency compared to comparison groups (see Table 2). There was an effect in favor of GSH CBT for ED pathology as well as frequency of binge eating in contrast to comparison groups. Residual heterogeneity was large for ED pathology and small for binge‐eating frequency (see Table 2). These results remained consistent in the sensitivity analyses (see Supporting Information).

##### 3.3.4.3 Treatment Duration

For ED pathology, the magnitude of the effect was moderated by treatment duration, with a longer treatment duration relating to smaller effects in favor of CBT (see Table 2). There was not enough variance in the moderator to investigate its role regarding binge‐eating frequency.

##### 3.3.4.4 Baseline Eating Disorder Severity

The magnitude of the effect was not moderated by baseline ED severity (see Table 2). This remained consistent in the sensitivity analyses (see Supporting Information).

##### 3.3.4.5 Publication Year

Publication year emerged as a significant moderator in samples with mixed ED diagnoses (see Table 2). A later publication year was associated with smaller effects for CBT regarding ED pathology. Variance was too small to investigate its role regarding binge‐eating frequency.

#### 3.3.5 Publication Bias

For studies investigating AN, the Egger test did not indicate publication bias for ED pathology, *b* = −0.39, *p* = 0.843, or BMI, *b* = 0.27, *p* = 0.889. There was an indication of publication bias for BN studies for ED pathology, *b* = −2.87, *p* = 0.033, but not for binge‐eating frequency, *b* = 0.56, *p* = 0.662, and frequency of compensatory behaviors, *b* = −1.10, *p* = 0.284. In studies investigating BED, the Egger test was significant for ED pathology, *b* = 3.79, *p* = 0.045, but not for binge‐eating frequency, *b* = 1.85, *p* = 0.296. For studies with mixed ED samples, there was an indication of publication for ED pathology, *b* = −3.21, *p* = 0.039, but not for binge‐eating frequency, *b* = −0.95, *p* = 0.579. Funnel plots for each outcome and ED diagnosis are available in the Supporting Information.

## 4 Discussion

This meta‐analysis investigated the efficacy of individual CBT for EDs. To this end, we included 42 RCTs including a total of 56 comparisons and conducted multivariate multilevel meta‐analyses. Overall, CBT emerged as an efficacious treatment in comparison to all comparison groups combined for BN, BED, and mixed ED diagnoses. When differentiating between comparison groups, CBT appears to not be superior to the heterogeneous group of active treatments that were used in the included RCTs; but it is consistently superior to waitlist comparison groups.

### 4.1 Anorexia Nervosa

For AN, studies were very scarce. Unfortunately, the largest available RCT (Zipfel et al. 2014) could not be included as descriptive statistics (i.e., unadjusted means) were not obtainable for meta‐analysis. However, their results were comparable to our findings as they reported no significant differences between CBT, focal psychodynamic therapy, and optimized TAU. In our meta‐analysis, CBT showed no superiority to other active treatments regarding ED pathology or BMI. This is in line with meta‐analytic results by Linardon, Wade, et al. (2017), van den Berg et al. (2019), and Solmi et al. (2021). Active treatments were very heterogeneous and ranged from less complex or theory‐driven treatment approaches like nonspecific supportive clinical management to theory‐driven treatments such as the Maudsley Anorexia Nervosa Treatment for Adults (MANTRA). Active treatments could not be analyzed separately as the number of studies with the same active treatments was too low. When considering other meta‐analytic evidence, a meta‐analysis on the effectiveness of MANTRA suggested that MANTRA is not superior to other psychological interventions (Fernández García and Quiles Marcos 2024) and despite promising evidence in Monteleone et al. (2022) network meta‐analysis for family therapy for young adults with AN, a Cochrane review from 2019 assessed the available studies mostly as low quality (Fisher et al. 2019). Recently, there have been efforts to improve CBT for AN, for example, by adapting it to the often severe and persistent nature of the disorder (Dalle Grave 2020). In addition, it was examined whether medication such as ketamine, which has shown promise for other disorders (Keeler et al. 2024), is effective in patients with AN. However, to date, there are not enough high‐quality studies to make an informed recommendation for the treatment of adult patients with AN based on meta‐analytic evidence.

### 4.2 Bulimia Nervosa

For BN, CBT was more efficacious with regard to ED pathology, binge‐eating frequency, and frequency of compensatory behaviors in comparison to waitlist, but not superior to active treatments. These findings are partly consistent with those of Linardon, Wade, et al. (2017), who also reported the superiority of therapist‐led individual CBT over inactive treatments for binge eating and purging. However, Linardon et al. additionally found therapist‐led individual CBT to outperform other active treatments—a discrepancy that can likely be attributed to methodological differences. First, for active controls, Linardon, Wade, et al. (2017) and Linardon, de la Piedad Garcia, and Brennan (2017) grouped together both psychotherapeutic and psychopharmacological interventions. Second, their analysis focused exclusively on therapist‐led treatment formats, the treatment format that was most consistently efficacious across outcomes in our study. In contrast, we compared individual CBT across treatment formats with psychotherapeutic interventions only. Third, we incorporated three randomized controlled trials (RCTs) on BN that were not included in Linardon et al.'s review, including an RCT comparing CBT‐E with mindfulness‐based therapy (Juarascio et al. 2021), which showed promising results for both approaches.

One explanation for the non‐significant difference post‐treatment between CBT and other active treatments pertains to substantial overlaps between CBT and other active treatments, specifically between regular CBT and third‐wave CBT (Juarascio et al. 2021; Steele and Wade 2008; Wonderlich et al. 2014) or CBT and behavioral treatment strategies (Cooper and Steere 1995). These similarities likely led to comparable results post‐treatment and it can be argued that these treatments belong to a broader CBT‐spectrum instead of distinct active treatments.

Our findings comply with Slade et al. (2018) who found superiority of individual CBT in comparison to waitlist in reaching remission. Slade et al. (2018) also drew a cautious conclusion that individual CBT and GSH CBT are more efficacious than other active treatments such as interpersonal therapy. However, they pointed out that the evidence base is limited, which still appears to be the case 7 years later. In our meta‐analysis, active treatments covered a range of approaches, from psychodynamic, systemic, mindfulness‐based to more eclectic treatments. Although BN was the most frequently studied diagnosis with regard to the number of eligible RCTs, only one RCT (Hartmann et al. 2024) compared CBT to TAU. This represents a relevant research gap as comparing evidence‐based treatments to what is routinely practiced can provide an answer to the question whether there is “a real benefit in turning to some other treatment beyond what is usually done” (Kazdin 2015, 171). The available study (Hartmann et al. 2024) suggests that CBT is more efficacious than TAU regarding binge eating, but not ED pathology or compensatory behaviors. Overall, more research is needed to investigate the efficacy of TAU in BN. Therapist‐led formats were consistently efficacious for all outcomes while GSH or pure SH were not. This aligns with Linardon, Wade, et al. (2017) finding that treatment outcomes were only consistently better for therapist‐led CBT in comparison to inactive controls, while for SH CBT, only remission rates and ED pathology were better, but not binge‐eating frequency and frequency of compensatory behaviors.

ED severity at baseline did not emerge as a relevant moderator for treatment outcome. However, it seems possible that the variance of ED severity was too small and the moderator analysis likely underpowered to detect a moderating effect of ED severity. Taking a closer look at our meta‐analytic sample, we found that ED severity at baseline was higher in studies using GSH or pure SH formats than in therapist‐led formats. In addition, we found that therapist‐led treatments, which consistently showed an effect across all outcomes, were administered over more weeks than GSH or pure SH that showed only effects for single outcomes. This confounding impedes interpretability of the moderator analyses. As GSH and pure SH are less intensive treatment options in a stepped‐care approach (Wilson et al. 2000; Musiat and Schmidt 2010), it seems relevant to note that in our meta‐analysis, samples who received GSH or pure SH tended to report greater baseline symptom severity than those in therapist‐led treatment formats. When disseminating and implementing evidence‐based treatments, SH treatments are more likely to be directed at individuals with less severe ED pathology, which points to a potential research‐practice gap. Overall, our results support that, at this point, in an individual therapy session, there is good evidence for the efficacy of therapist‐led CBT for BN. While we cannot conclude from our findings that CBT is clearly better than other treatments, which contrasts international treatment guidelines (Hilbert et al. 2017), it shows robust evidence, especially for binge‐eating frequency.

### 4.3 Binge‐Eating Disorder

For BED, CBT was efficacious regarding ED pathology and binge‐eating frequency, which is in line with previous meta‐analytic evidence (Ghaderi et al. 2018; Hilbert et al. 2019). However, CBT was not more efficacious than other active treatments regarding ED pathology or binge‐eating frequency, which complies with the analysis of individual therapy by Linardon, Wade, et al. (2017) and the findings by Hilbert et al. (2019) when comparing CBT to other psychotherapies. To add to this, Hilbert et al. (2019) reported in their meta‐analysis that CBT differed only from humanist therapies and only for binge‐eating abstinence, while CBT did not differ from more ED‐specific bona fide treatments.

In our meta‐analysis, baseline severity of ED pathology in BED samples was comparable to that of samples with AN and BN. However, they were exclusively treated using GSH or pure SH. A reason for this might be that GSH treatments emerged at the same time as the diagnosis of BED, suggesting that the lack of studies using other treatment formats for this diagnosis might have been due to a research trend. Another reason might be that a less intensive treatment format is repeatedly chosen for BED as it is generally seen as less severe and easier to treat by the public (Reas 2017) which is contradicted by research (Giel et al. 2022). Research is needed to investigate if therapist‐led individual CBT outperforms GSH or pure SH formats for BED. Our moderator analysis indicated that treatment effects of CBT were larger for those with greater baseline symptom severity. This extends the meta‐analysis by Hilbert et al. (2019) which found that samples with more frequent baseline binge‐eating episodes experienced greater reduction in binge‐eating episodes. However, evidence at this point is inconclusive since changes and post‐scores reflect different phenomena, underlining the need for more research on moderators for treatment response in EDs (also see Linardon, de la Piedad Garcia, and Brennan 2017).

Our results suggest that for individual therapy, CBT is clearly better than not undergoing treatment. However, CBT does not outperform other psychotherapies such as interpersonal therapy or integrative cognitive‐affective therapy (ICAT) as well as behavioral weight loss treatment in an individual treatment setting, which questions the clear recommendation of GSH for BED in treatment guidelines (National Institute for Health and Care Excellence 2017).

### 4.4 Mixed Eating Disorder Diagnoses

This is the first meta‐analysis systematically investigating mixed ED diagnoses. For mixed ED diagnoses, we found that CBT was more efficacious regarding ED pathology and binge‐eating frequency than waitlist and more efficacious considering ED pathology than TAU but not different from the active comparisons. This is a relevant finding and plausible in that, for example, general practitioners or nutrition counselors would advise establishing a regular meal pattern, which would likely help binge eating to subside. At the same time, not all professionals are equipped to help deal with dysfunctional cognitions or feelings that also tend to take longer to subside (Wilson et al. 2000). This may explain why it takes more targeted treatments such as CBT to address overall ED pathology that encompasses, for instance, suppressing or ruminating on unwanted emotions (Dingemans et al. 2017), for example, guilt (Schaefer et al. 2020). The fact that GSH emerged as favorable in comparison to other treatment formats should be interpreted cautiously, as only 27% of the studies in mixed EDs investigated other treatment formats. Interestingly, samples with mixed ED diagnoses were those with the greatest symptom severity and, following AN samples, the second longest treatment duration. For ED pathology overall, longer treatment durations were associated with smaller effects in favor of CBT. Notably, the two most influential studies in the analysis—Fitzsimmons‐Craft et al. (2020; *n* = 570) and ter Huurne et al. (2015; *n* = 201)—had the largest sample sizes but relatively short treatment durations (32 and 15 weeks, respectively). Both studies used TAU or waitlist control groups, which may have contributed to larger observed effects for CBT. This suggests that treatment duration may have been confounded with the type of comparison group. As such, caution is warranted when interpreting the moderator analysis of treatment duration.

Later publication years were associated with smaller effects in ED pathology for CBT. However, there are no clearly identifiable factors that explain this trend. Later studies did not consistently show a lower risk of bias, higher baseline symptom severity, or more conservative control conditions (i.e., active treatment comparisons) which might otherwise account for the reduced effects. We suggest that future meta‐analyses should investigate if this moderator effect replicates.

Mixed ED samples had not been investigated separately by Linardon, Wade, et al. (2017). However, our findings underline the necessity to include mixed ED samples and EDNOS and OSFED diagnoses in meta‐analytic syntheses as they feature differential severity, treatment duration, and treatment responses. Regarding treatment implications, our study cautiously suggests that individuals with unclear symptomatology should be offered a GSH CBT approach as it was efficacious for both overall ED pathology and behavioral symptoms.

### 4.5 Strengths and Limitations of the Present Meta‐Analysis

Strengths include that first, all information was extracted from the studies by two coders, improving the reliability of our meta‐analytic results. Second, we conducted risk of bias assessments and ran sensitivity analyses to explore the potential for bias arising from differential attrition and low study quality. Third, we conducted separate analyses for ED diagnoses, included mixed ED diagnoses, and used multivariate multilevel meta‐analyses to account for dependencies in the hierarchical nature of the study data. Overall, the rather high granularity (i.e., investigating individual CBT for adults in contrast to other psychotherapies, TAU, or waitlist) allowed us to draw differentiated conclusions.

Our meta‐analysis also has limitations: First, despite these narrow inclusion criteria, especially regarding effects on ED pathology, there was great heterogeneity that limits interpretability. Second, the heterogeneity of active comparison groups, which included both unspecific and ED‐specific complex approaches, limited possible subanalyses about the efficacy of specific alternatives to CBT. Of the included studies with an active comparison group, only 50% of AN studies and 40% of BN studies had sample sizes large enough to detect large effects with a power of 0.80. If effects were smaller, the required sample sizes would have to be even larger. It is possible that a lack of power prevented us from finding differences between CBT and active treatments. Third, all studies that were included were from Western parts of the world, and patients were mainly White, female, and rather educated. Moreover, many studies did not consistently report ethnicity, race, and socioeconomic status, which represents a relevant limitation of the included literature. Results thus cannot be generalized to other parts of the world, men, or non‐binary individuals with ED. Fourth, although we did search for gray literature in ProQuest and trial registries, other databases such as Google Scholar were not included. Fifth, we were unable to conduct analyses for follow‐up time points, as only 15 studies reported follow‐up data, and time scales differed greatly. Hence, we cannot draw conclusions regarding the stability of treatment effects. Sixth, only 18 studies had a risk of bias assessment that was good, which highlights the need for further high‐quality RCTs in this context. Beyond that, there is a paucity of studies comparing CBT to alternative treatments for AN, as well as a lack of studies examining therapist‐led treatment formats for patients with severe BED.

## 5 Conclusion

Our meta‐analysis synthesized treatment studies for individual CBT for adults with EDs over almost four decades of research. In line with previous meta‐analyses, CBT consistently outperformed waitlist comparisons for ED pathology and binge eating and frequency of compensatory behaviors in BN, BED, and mixed disorders, which justifies its role in treatment guidelines. However, other than indicated by Linardon, Wade, et al. (2017), CBT did not clearly emerge as more efficacious than active treatments, which were a heterogeneous group of treatments that ranged from unspecific treatments to evidence‐based theory‐driven approaches. Although weight gain is the key treatment goal for AN, BMI was not consistently reported, and descriptively, CBT even showed lower post‐treatment BMI than other active treatments. This represents a crucial area for future research. With regard to different treatment formats, more intensive and guided treatment formats tended to show a more consistent effect than unguided treatment formats. Neither ED severity at baseline nor treatment duration or publication year emerged as consistent moderators of CBT efficacy. However, these findings have to be interpreted cautiously because of methodological limitations, such as potential confounding. Future research should target RCTs investigating specialized treatments for AN, compare CBT for BN to the clinical care as ordinarily practiced, and conduct trials that examine therapist‐led treatments for BED.

## Author Contributions

**Jana Bruns:** conceptualization, investigation, writing – original draft, writing – review and editing, methodology, formal analysis, data curation. **Marieke Meier:** conceptualization, writing – review and editing, writing – original draft. **Katrin Jansen:** supervision, writing – review and editing, conceptualization, methodology.

## Conflicts of Interest

The authors declare no conflicts of interest.

## Supporting information


**Data S1:** Supporting information.

## Data Availability

Code, data, and Supporting Information are available at https://osf.io/5t78f/.
