# Supplementary material for: The Efficacy of Individual Cognitive Behavioral Therapy for Eating Disorders: A Meta‐Analysis of Randomized Controlled Trials
Source: Int J Eat Disord. 2025 Aug 14;58(11):2029–57. doi: 10.1002/eat.24519 (PMC12605742; doi:10.1002/eat.24519)
Supplement: Supplementary file 1 — Data S1: Supporting information. [file EAT-58-2029-s001.pdf]

Supplemental Material for the Article

„The Efficacy of Individual Cognitive-Behavioral Therapy for Eating Disorder: A Meta-Analysis of Randomized Controlled Trials"

Jana Bruns*[1], Marieke Meier*[1] & Katrin Jansen[1]

[1]University of Münster

*shared first authorship

Forest Plots 5

Figure S1. *Forest Plot for Studies Investigating Anorexia Nervosa: Eating Disorder Pathology* 5

Figure S2. *Forest Plot for Studies Investigating Anorexia Nervosa: Body Mass Index* 6

Figure S3. *Forest Plot for Studies Investigating Bulimia Nervosa: Eating Disorder Pathology* 7

Figure S4. *Forest Plot for Studies Investigating Bulimia Nervosa: Binge-Eating Frequency* 9

Figure S5. *Forest Plot for Studies Investigating Bulimia Nervosa: Frequency of Compensatory Behavior* 10

Figure S6. *Forest Plot for Studies Investigating Binge-Eating Disorder: Eating Disorder Pathology* 11

Figure S7. *Forest Plot for Studies Investigating Binge-Eating Disorder: Binge-Eating Frequency* ... 12

Figure S8. *Forest Plot for Studies Investigating Mixed Eating Disorders: Eating Disorder Pathology* 13

Figure S9. *Forest Plot for Studies Investigating Mixed Eating Disorders: Binge-Eating Frequency* . 14

Scatterplots for Continuous Moderators 15

Figure S10. *Scatterplot Depicting Effect Sizes for Eating Disorder Pathology and Treatment Duration for Studies Investigating Anorexia Nervosa* 15

Figure S11. *Scatterplot Depicting Effect Sizes for Body Mass Index and Treatment Duration for Studies Investigating Anorexia Nervosa* 15

Figure S12. *Scatterplot Depicting Effect Sizes for Eating Disorder Pathology and Baseline Eating Disorder Severity for Studies Investigating Anorexia Nervosa* 16

Figure S13. *Scatterplot Depicting Effect Sizes for Body Mass Index and Baseline Eating Disorder Severity for Studies Investigating Anorexia Nervosa* 16

Figure S14. *Scatterplot Depicting Effect Sizes for Eating Disorder Pathology and Publication Year for Studies Investigating Anorexia Nervosa* 17

Figure S15. *Scatterplot Depicting Effect Sizes for Body Mass Index and Publication Year for Studies Investigating Anorexia Nervosa* 17

Figure S16. *Scatterplot Depicting Effect Sizes for Eating Disorder Pathology and Treatment Duration for Studies Investigating Bulimia Nervosa* 18

Figure S17. *Scatterplot Depicting Effect Sizes for Binge-Eating Frequency and Treatment Duration for Studies Investigating Bulimia Nervosa* 18

Figure S18. *Scatterplot Depicting Effect Sizes for Frequency of Compensatory Behaviors and Treatment Duration for Studies Investigating Bulimia Nervosa* 19

Figure S19. *Scatterplot Depicting Effect Sizes for Eating Disorder Pathology and Baseline Eating Disorder Severity for Studies Investigating Bulimia Nervosa* 20

Figure S20. *Scatterplot Depicting Effect Sizes for Binge-Eating Frequency and Baseline Eating Disorder Severity for Studies Investigating Bulimia Nervosa* 20

Figure S21. *Scatterplot Depicting Effect Sizes for Frequency of Compensatory Behaviors and Eating Disorder Pathology for Studies Investigating Bulimia Nervosa* 21

Figure S22. *Scatterplot Depicting Effect Sizes for Eating Disorder Pathology and Publication Year for Studies Investigating Bulimia Nervosa* 22

Figure S23. *Scatterplot Depicting Effect Sizes for Binge-Eating Frequency and Publication Year for Studies Investigating Bulimia Nervosa* 22

Figure S24. *Scatterplot Depicting Effect Sizes for Frequency of Compensatory Behaviors and Publication Year for Studies Investigating Bulimia Nervosa* 23

Figure S25. *Scatterplot Depicting Effect Sizes for Eating Disorder Pathology and Treatment Duration for Studies Investigating Binge-Eating Disorder* 24

Figure S26. *Scatterplot Depicting Effect Sizes for Binge-Eating Frequency and Treatment Duration for Studies Investigating Binge-Eating Disorder* 24

Figure S27. *Scatterplot Depicting Effect Sizes for Eating Disorder Pathology and Baseline Eating Disorder Severity for Studies Investigating Binge-Eating Disorder* 25

Figure S28. *Scatterplot Depicting Effect Sizes for Binge-Eating Frequency and Baseline Eating Disorder Severity for Studies Investigating Binge-Eating Disorder* 25

Figure S29. *Scatterplot Depicting Effect Sizes for Eating Disorder Pathology and Publication Year for Studies Investigating Binge-Eating Disorder* 26

Figure S30. *Scatterplot Depicting Effect Sizes for Binge-Eating Frequency and Publication Year for Studies Investigating Binge-Eating Disorder* 26

Figure S31. *Scatterplot Depicting Effect Sizes for Eating Disorder Pathology and Treatment Duration for Studies Investigating Mixed Eating Disorders* 27

Figure S32. *Scatterplot Depicting Effect Sizes for Binge-Eating Frequency and Treatment Duration for Studies Investigating Mixed Eating Disorders* 27

Figure S33. *Scatterplot Depicting Effect Sizes for Eating Disorder Pathology and Baseline Eating Disorder Severity for Studies Investigating Mixed Eating Disorders* 28

Figure S34. *Scatterplot Depicting Effect Sizes for Binge-Eating Frequency and Baseline Eating Disorder Severity for Studies Investigating Mixed Eating Disorders* 28

Figure S35. *Scatterplot Depicting Effect Sizes for Eating Disorder Pathology and Publication Year for Studies Investigating Mixed Eating Disorders* 29

Figure S36. *Scatterplot Depicting Effect Sizes for Binge-Eating Frequency and Publication Year for Studies Investigating Mixed Eating Disorders* 29

Results of the Sensitivity Analyses 30

Anorexia Nervosa Studies 30

Bulimia Nervosa Studies 32

Binge-Eating Disorder Studies 42

Mixed Eating Disorders Studies 51

Funnel Plots 59

Figure S37. *Funnel Plot for Studies Investigating Anorexia Nervosa: Eating Disorder Pathology* 59

Figure S38. *Funnel Plot for Studies Investigating Anorexia Nervosa: Body Mass Index* 59

Figure S39. *Funnel Plot for Studies Investigating Bulimia Nervosa: Eating Disorder Pathology* 60

Figure S40. *Funnel Plot for Studies Investigating Bulimia Nervosa: Binge-Eating Frequency* 60

Figure S41. *Funnel Plot for Studies Investigating Bulimia Nervosa: Frequency of Compensatory Behavior* 61

Figure S42. *Funnel Plot for Studies Investigating Binge-Eating Disorder: Eating Disorder Pathology* 62

Figure S43. *Funnel Plot for Studies Investigating Binge-Eating Disorder: Binge-Eating Frequency* 62

Figure S44. *Funnel Plot for Studies Investigating Mixed Eating Disorders: Eating Disorder Pathology* 63

Figure S45. *Funnel Plot for Studies Investigating Mixed Eating Disorders: Binge-Eating Frequency* 63

Non-English Citations 64

Forest Plots

Figure S1. *Forest Plot for Studies Investigating Anorexia Nervosa: Eating Disorder Pathology*

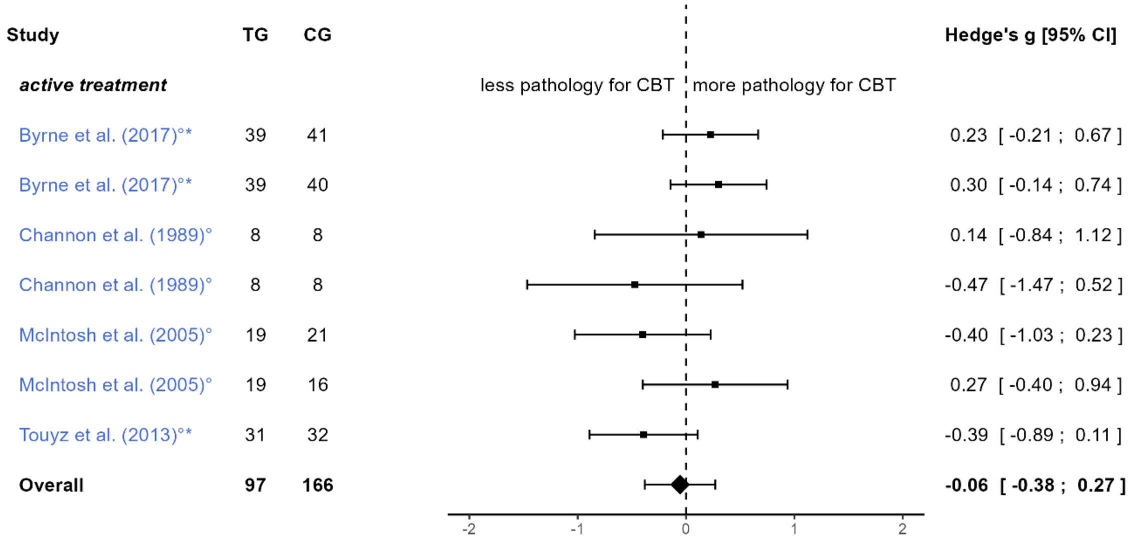

Note. TG = treatment group, CG = comparison group, CI = confidence interval; studies are sorted by type of comparison group; colors indicate the treatment format in the treatment group: blue = therapist-led, green = guided self-help, lightgreen = pure self-help; ° indicates that the study used intention-to-treat analysis; * indicates that the study has a quality score of 7 or 8; the whiskers represent 95% confidence intervals; results in bold and italic indicate results from subgroup analyses; results in bold indicate results from the main analysis.

Figure S2. *Forest Plot for Studies Investigating Anorexia Nervosa: Body Mass Index*

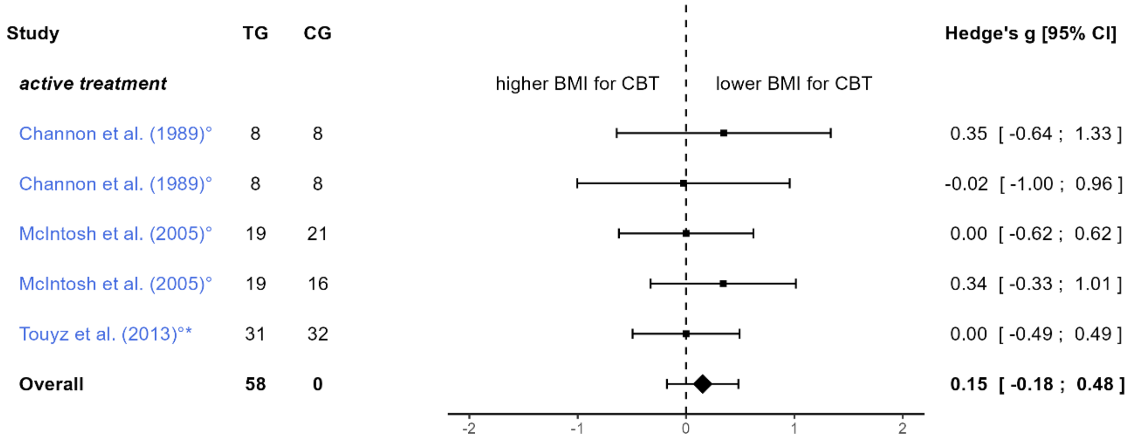

Note. TG = treatment group, CG = comparison group, CI = confidence interval; studies are sorted by type of comparison group; colors indicate the treatment format in the treatment group: blue = therapist-led, green = guided self-help, lightgreen = pure self-help; ° indicates that the study used intention-to-treat analysis; * indicates that the study has a quality score of 7 or 8; the whiskers represent 95% confidence intervals; results in bold and italic indicate results from subgroup analyses; results in bold indicate results from the main analysis.

Figure S3. *Forest Plot for Studies Investigating Bulimia Nervosa: Eating Disorder Pathology*

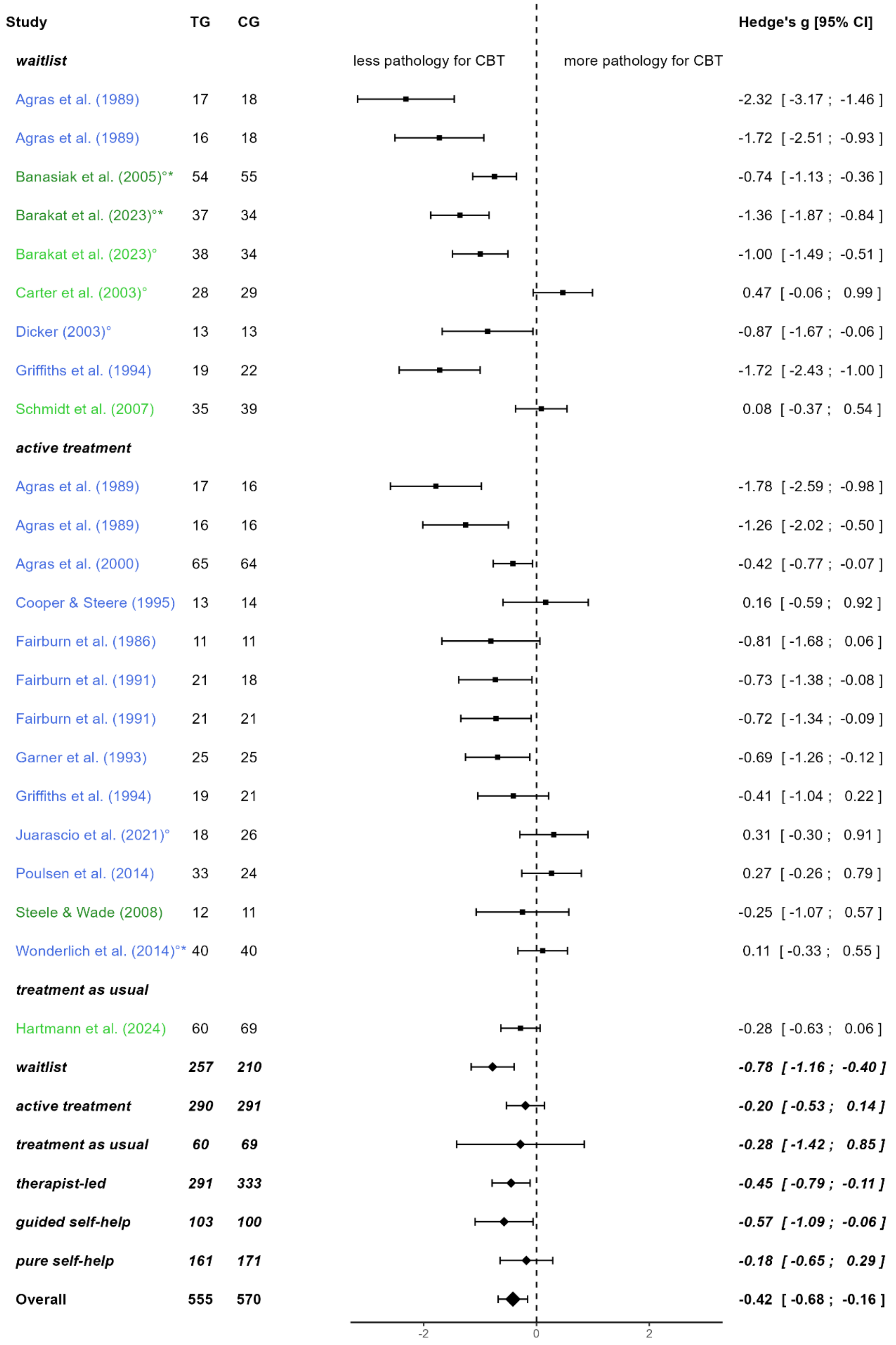

Note. TG = treatment group, CG = comparison group, CI = confidence interval; studies are sorted by type of comparison group; colors indicate the treatment format in the treatment group: blue = therapist-

led, green = guided self-help, lightgreen = pure self-help; ° indicates that the study used intention-to-treat analysis; * indicates that the study has a quality score of 7 or 8; the whiskers represent 95% confidence intervals; results in bold and italic indicate results from subgroup analyses; results in bold indicate results from the main analysis.

Figure S4. *Forest Plot for Studies Investigating Bulimia Nervosa: Binge-Eating Frequency*

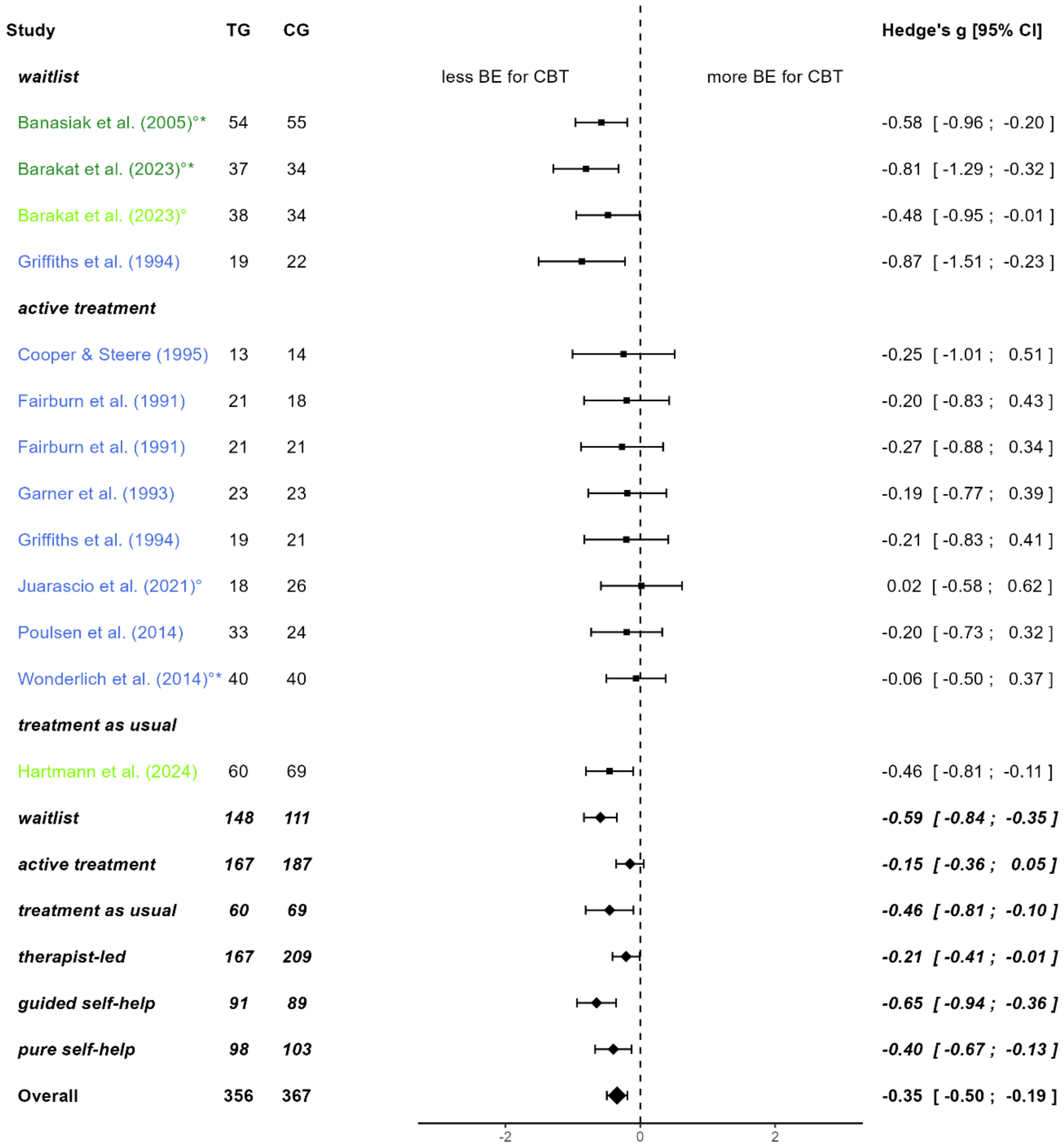

Note. TG = treatment group, CG = comparison group, CI = confidence interval, BE = binge-eating; studies are sorted by type of comparison group; colors indicate the treatment format in the treatment group: blue = therapist-led, green = guided self-help, lightgreen = pure self-help; ° indicates that the study used intention-to-treat analysis; * indicates that the study has a quality score of 7 or 8; the whiskers represent 95% confidence intervals; results in bold and italic indicate results from subgroup analyses; results in bold indicate results from the main analysis.

Figure S5. *Forest Plot for Studies Investigating Bulimia Nervosa: Frequency of Compensatory Behavior*

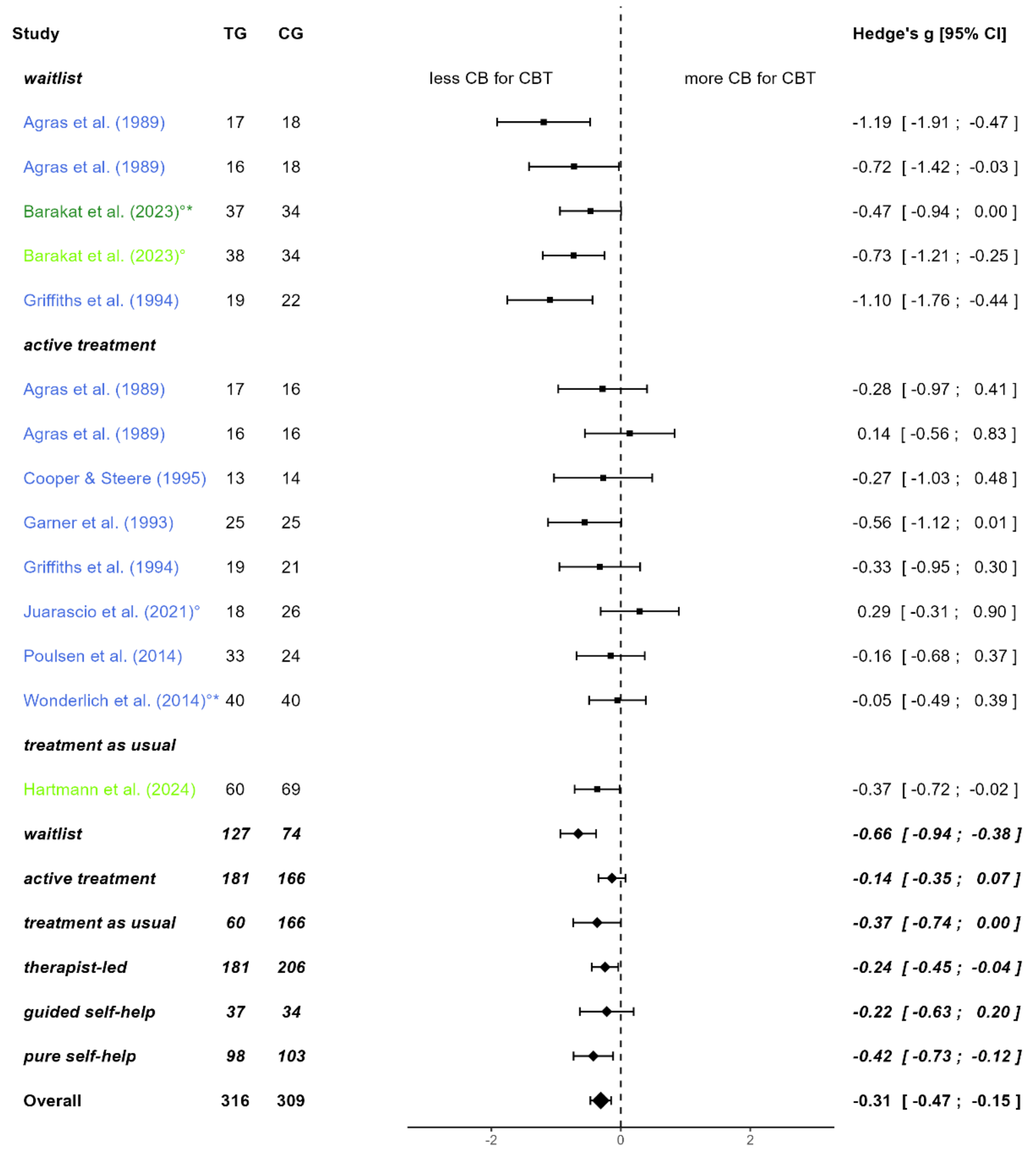

Note. TG = treatment group, CG = comparison group, CI = confidence interval, CB = compensatory behaviors; studies are sorted by type of comparison group; colors indicate the treatment format in the treatment group: blue = therapist-led, green = guided self-help, lightgreen = pure self-help; ° indicates that the study used intention-to-treat analysis; * indicates that the study has a quality score of 7 or 8; the whiskers represent 95% confidence intervals; results in bold and italic indicate results from subgroup analyses; results in bold indicate results from the main analysis.

Figure S6. *Forest Plot for Studies Investigating Binge-Eating Disorder: Eating Disorder Pathology*

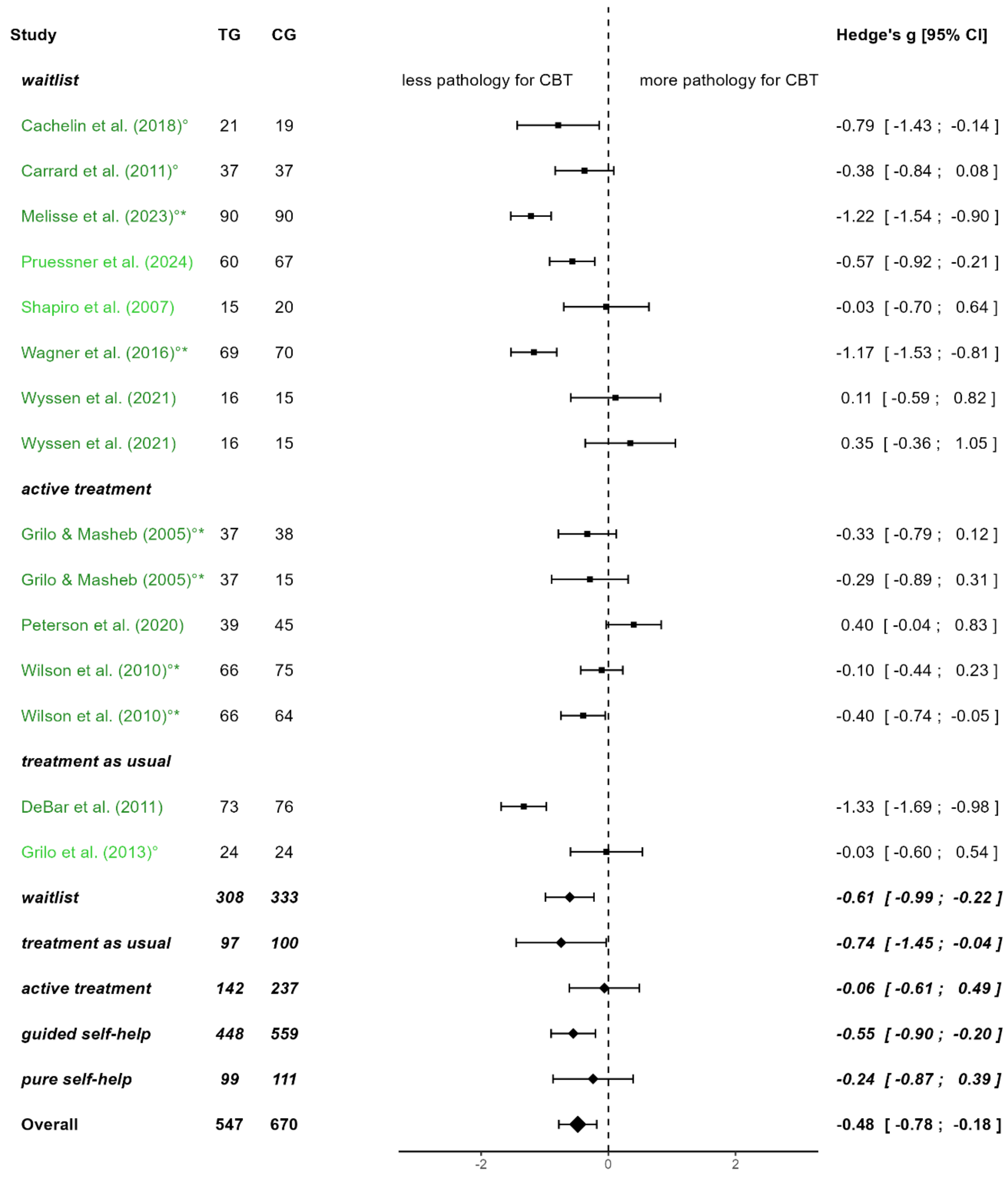

Note. TG = treatment group, CG = comparison group, CI = confidence interval; studies are sorted by type of comparison group; colors indicate the treatment format in the treatment group: blue = therapist-led, green = guided self-help, lightgreen = pure self-help; ° indicates that the study used intention-to-treat analysis; * indicates that the study has a quality score of 7 or 8; the whiskers represent 95% confidence intervals; results in bold and italic indicate results from subgroup analyses; results in bold indicate results from the main analysis.

Figure S7. *Forest Plot for Studies Investigating Binge-Eating Disorder: Binge-Eating Frequency*

Study	TG	CG	Hedge's g [95% CI]
waitlist			
Cachelin et al. (2018)°	21	19	-1.16 [-1.83 ; -0.49]
Carrard et al. (2011)°	37	37	-0.44 [-0.90 ; 0.02]
Melisse et al. (2023)°*	90	90	-1.00 [-1.31 ; -0.69]
Pruessner et al. (2024)	60	67	-0.60 [-0.96 ; -0.25]
Shapiro et al. (2007)	15	20	0.06 [-0.61 ; 0.73]
Wagner et al. (2016)°*	69	70	-1.06 [-1.41 ; -0.70]
Wyssen et al. (2021)	16	15	-0.29 [-0.99 ; 0.42]
Wyssen et al. (2021)	16	15	0.88 [0.14 ; 1.62]
active treatment			
Grilo & Masheb (2005)°*	37	38	-0.57 [-1.04 ; -0.11]
Grilo & Masheb (2005)°*	37	15	-0.92 [-1.55 ; -0.30]
Wilson et al. (2010)°*	66	75	0.01 [-0.32 ; 0.34]
Wilson et al. (2010)°*	66	64	-0.07 [-0.41 ; 0.28]
treatment as usual			
Grilo et al. (2013)°	24	24	-0.11 [-0.68 ; 0.45]
waitlist	***308***	***333***	***-0.60 [-0.95 ; -0.25]***
treatment as usual	***24***	***24***	***-0.41 [-1.34 ; 0.51]***
active treatment	***103***	***192***	***-0.24 [-0.83 ; 0.35]***
guided self-help	***336***	***438***	***-0.58 [-0.91 ; -0.24]***
pure self-help	***99***	***111***	***-0.26 [-0.81 ; 0.29]***
**Overall**	**435**	**549**	**-0.50 [-0.77 ; -0.22]**

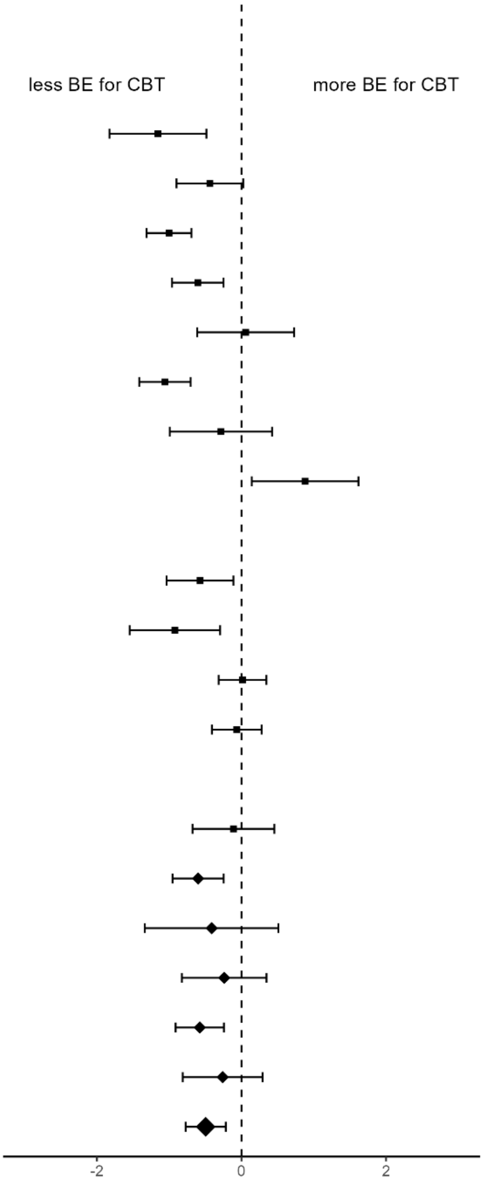

Note. TG = treatment group, CG = comparison group, CI = confidence interval, BE = binge-eating; studies are sorted by type of comparison group; colors indicate the treatment format in the treatment group: blue = therapist-led, green = guided self-help, lightgreen = pure self-help; ° indicates that the study used intention-to-treat analysis; * indicates that the study has a quality score of 7 or 8; the whiskers represent 95% confidence intervals; results in bold and italic indicate results from subgroup analyses; results in bold indicate results from the main analysis.

Figure S8. *Forest Plot for Studies Investigating Mixed Eating Disorders: Eating Disorder Pathology*

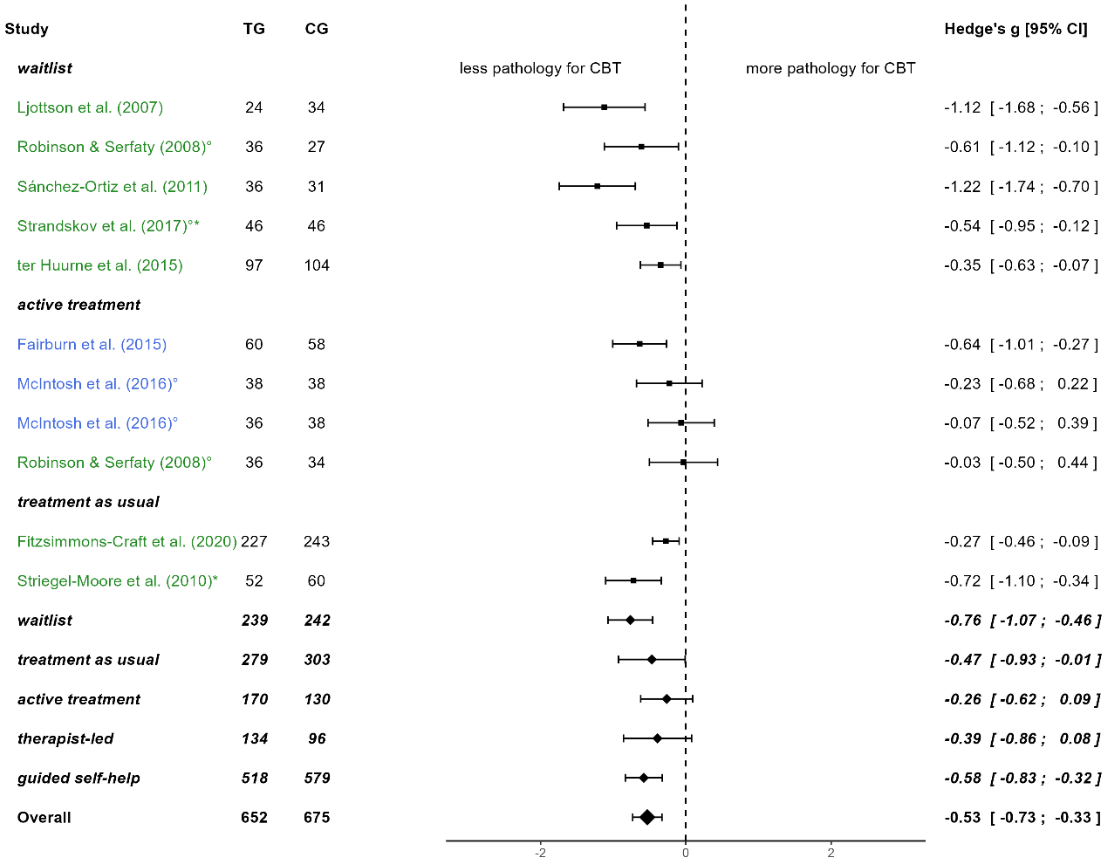

Note. TG = treatment group, CG = comparison group, CI = confidence interval; studies are sorted by type of comparison group; colors indicate the treatment format in the treatment group: blue = therapist-led, green = guided self-help, lightgreen = pure self-help; ° indicates that the study used intention-to-treat analysis; * indicates that the study has a quality score of 7 or 8; the whiskers represent 95% confidence intervals; results in bold and italic indicate results from subgroup analyses; results in bold indicate results from the main analysis.

Figure S9. *Forest Plot for Studies Investigating Mixed Eating Disorders: Binge-Eating Frequency*

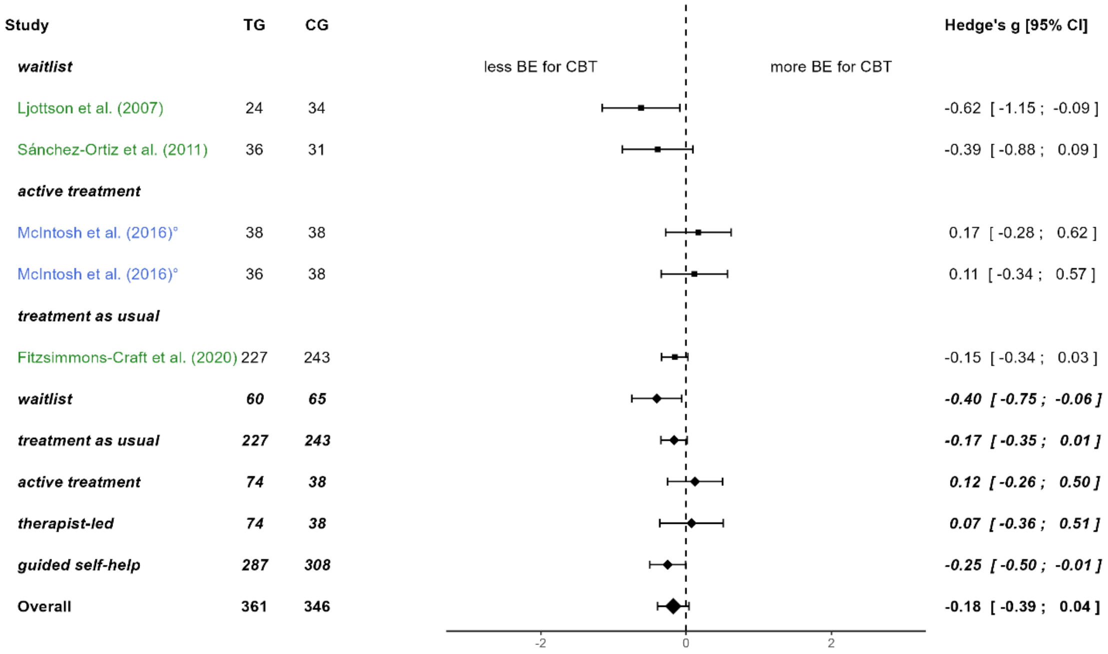

Note. TG = treatment group, CG = comparison group, CI = confidence interval, BE = binge-eating; studies are sorted by type of comparison group; colors indicate the treatment format in the treatment group: blue = therapist-led, green = guided self-help, lightgreen = pure self-help; ° indicates that the study used intention-to-treat analysis; * indicates that the study has a quality score of 7 or 8; the whiskers represent 95% confidence intervals; results in bold and italic indicate results from subgroup analyses; results in bold indicate results from the main analysis.

Scatterplots for Continuous Moderators

Figure S10. *Scatterplot Depicting Effect Sizes for Eating Disorder Pathology and Treatment Duration for Studies Investigating Anorexia Nervosa*

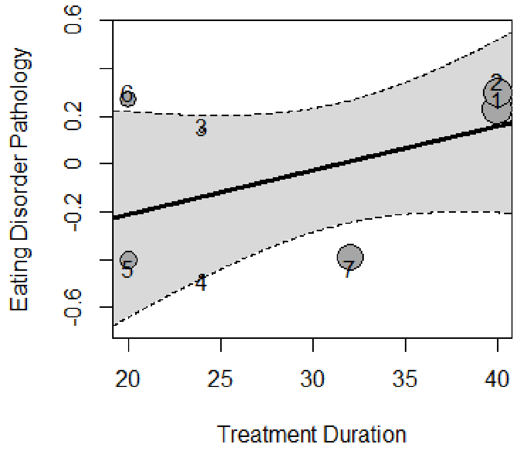

Note. size of circles indicates sample size.

Figure S11. *Scatterplot Depicting Effect Sizes for Body Mass Index and Treatment Duration for Studies Investigating Anorexia Nervosa*

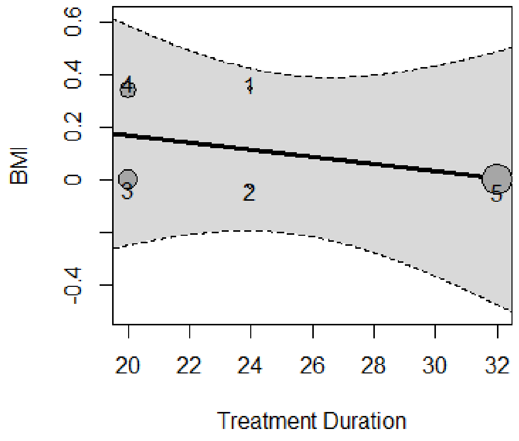

Note. size of circles indicates sample size.

Figure S12. *Scatterplot Depicting Effect Sizes for Eating Disorder Pathology and Baseline Eating Disorder Severity for Studies Investigating Anorexia Nervosa*

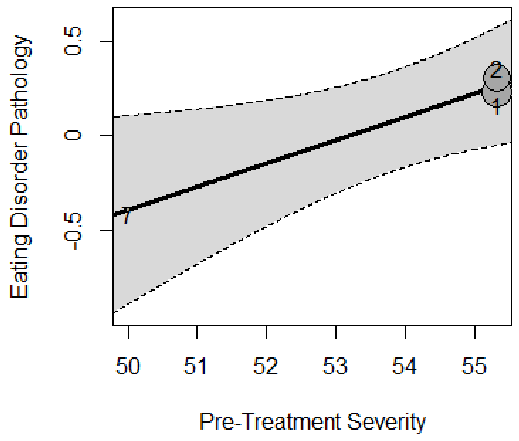

Note. size of circles indicates sample size.

Figure S13. *Scatterplot Depicting Effect Sizes for Body Mass Index and Baseline Eating Disorder Severity for Studies Investigating Anorexia Nervosa*

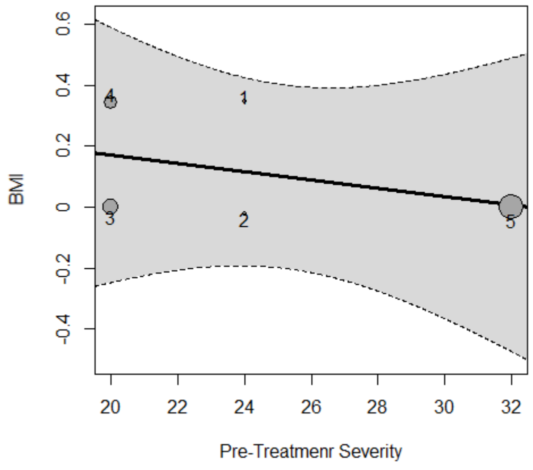

Note. size of circles indicates sample size.

Figure S14. *Scatterplot Depicting Effect Sizes for Eating Disorder Pathology and Publication Year for Studies Investigating Anorexia Nervosa*

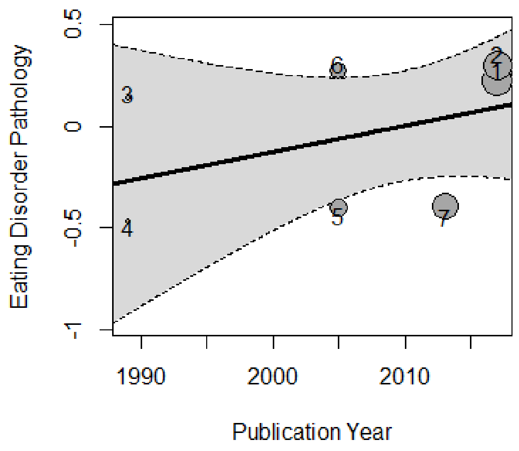

Note. size of circles indicates sample size.

Figure S15. *Scatterplot Depicting Effect Sizes for Body Mass Index and Publication Year for Studies Investigating Anorexia Nervosa*

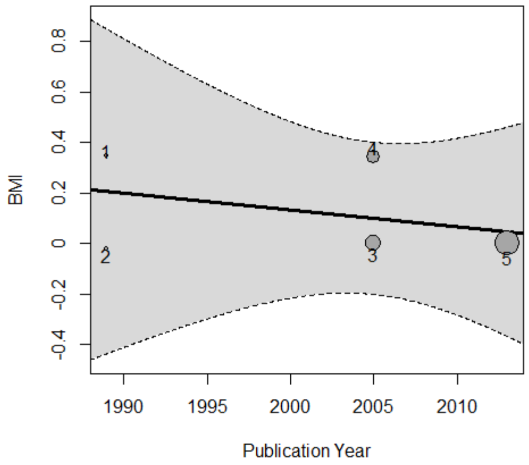

Note. size of circles indicates sample size.

Figure S16. *Scatterplot Depicting Effect Sizes for Eating Disorder Pathology and Treatment Duration for Studies Investigating Bulimia Nervosa*

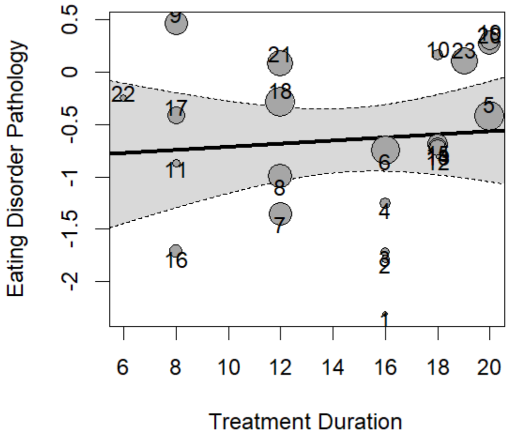

Note. size of circles indicates sample size.

Figure S17. *Scatterplot Depicting Effect Sizes for Binge-Eating Frequency and Treatment Duration for Studies Investigating Bulimia Nervosa*

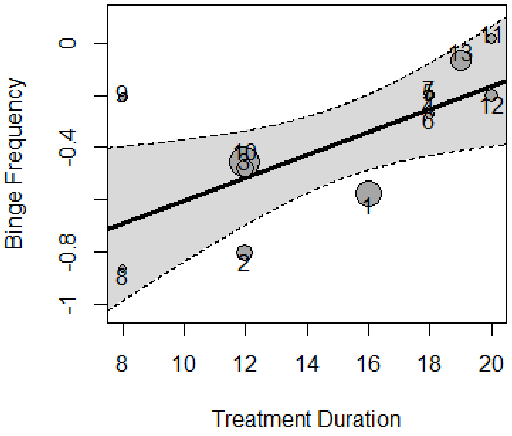

Note. size of circles indicates sample size.

Figure S18. *Scatterplot Depicting Effect Sizes for Frequency of Compensatory Behaviors and Treatment Duration for Studies Investigating Bulimia Nervosa*

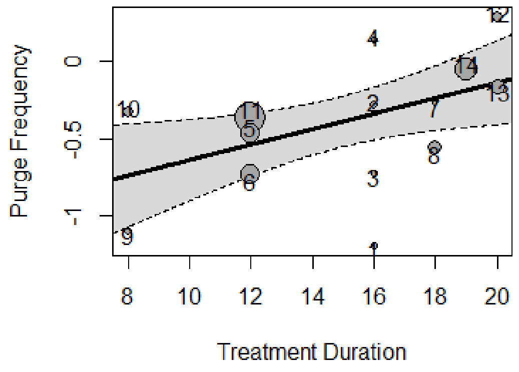

Note. size of circles indicates sample size.

Figure S19. *Scatterplot Depicting Effect Sizes for Eating Disorder Pathology and Baseline Eating Disorder Severity for Studies Investigating Bulimia Nervosa*

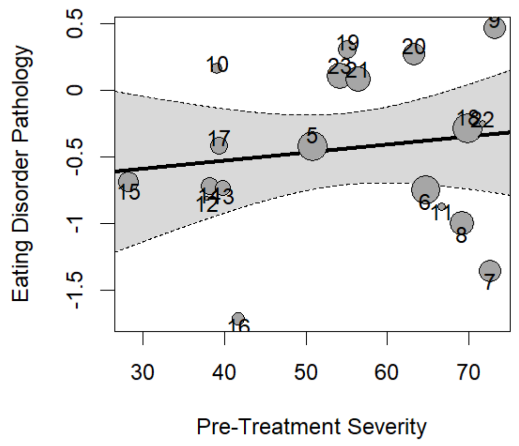

Note. size of circles indicates sample size.

Figure S20. *Scatterplot Depicting Effect Sizes for Binge-Eating Frequency and Baseline Eating Disorder Severity for Studies Investigating Bulimia Nervosa*

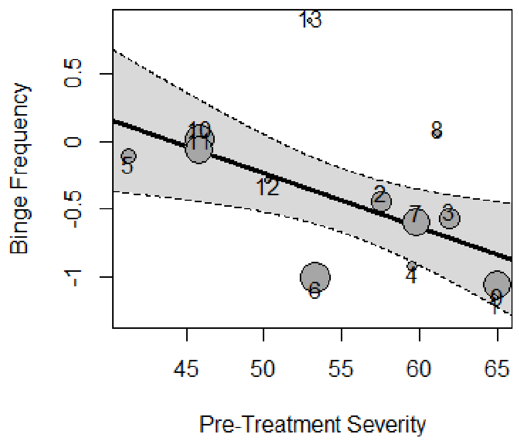

Note. size of circles indicates sample size.

Figure S21. *Scatterplot Depicting Effect Sizes for Frequency of Compensatory Behaviors and Eating Disorder Pathology for Studies Investigating Bulimia Nervosa*

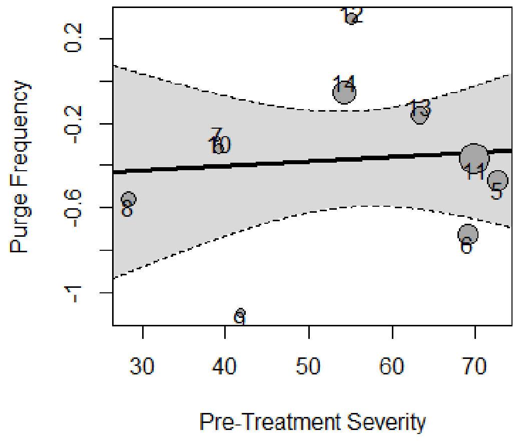

Note. size of circles indicates sample size.

Figure S22. *Scatterplot Depicting Effect Sizes for Eating Disorder Pathology and Publication Year for Studies Investigating Bulimia Nervosa*

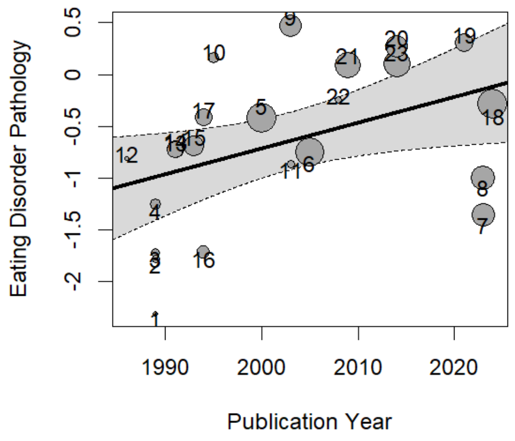

Note. size of circles indicates sample size.

Figure S23. *Scatterplot Depicting Effect Sizes for Binge-Eating Frequency and Publication Year for Studies Investigating Bulimia Nervosa*

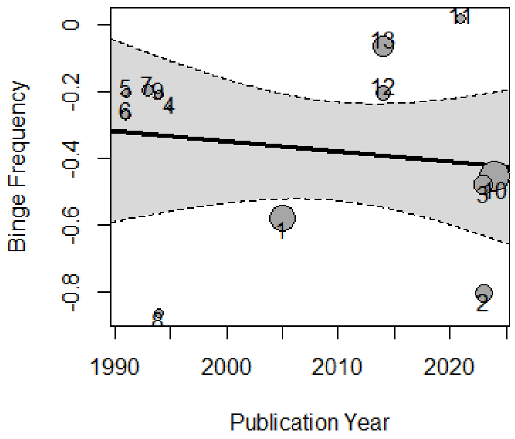

Note. size of circles indicates sample size.

Figure S24. *Scatterplot Depicting Effect Sizes for Frequency of Compensatory Behaviors and Publication Year for Studies Investigating Bulimia Nervosa*

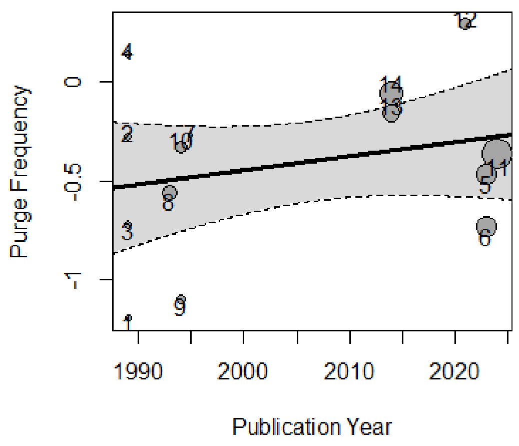

Note. size of circles indicates sample size.

Figure S25. *Scatterplot Depicting Effect Sizes for Eating Disorder Pathology and Treatment Duration for Studies Investigating Binge-Eating Disorder*

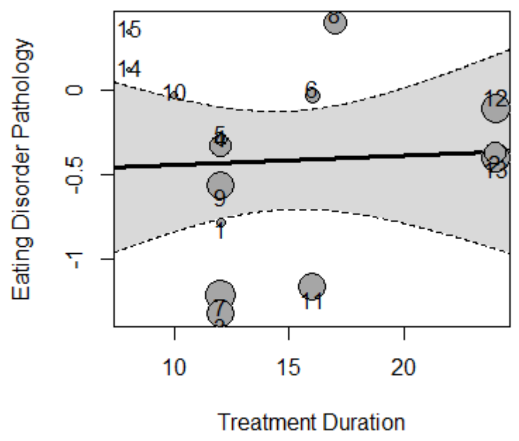

Note. size of circles indicates sample size.

Figure S26. *Scatterplot Depicting Effect Sizes for Binge-Eating Frequency and Treatment Duration for Studies Investigating Binge-Eating Disorder*

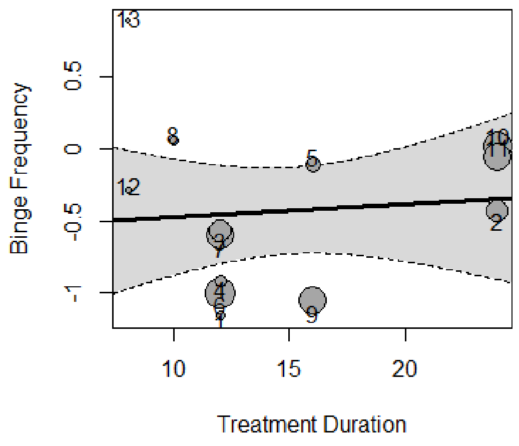

Note. size of circles indicates sample size.

Figure S27. *Scatterplot Depicting Effect Sizes for Eating Disorder Pathology and Baseline Eating Disorder Severity for Studies Investigating Binge-Eating Disorder*

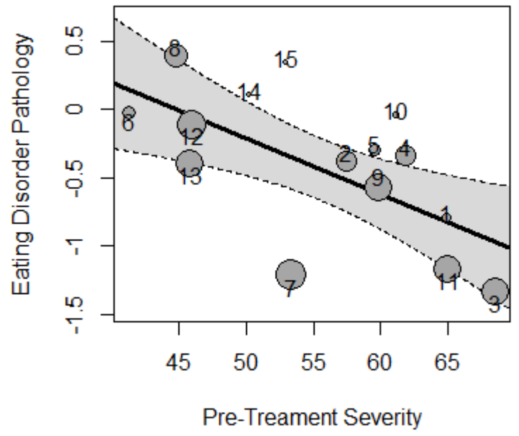

Note. size of circles indicates sample size.

Figure S28. *Scatterplot Depicting Effect Sizes for Binge-Eating Frequency and Baseline Eating Disorder Severity for Studies Investigating Binge-Eating Disorder*

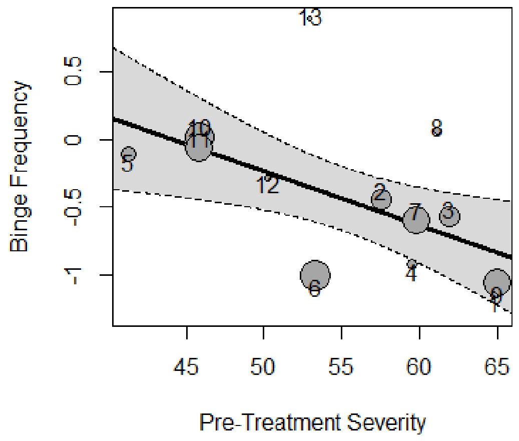

Note. size of circles indicates sample size.

Figure S29. *Scatterplot Depicting Effect Sizes for Eating Disorder Pathology and Publication Year for Studies Investigating Binge-Eating Disorder*

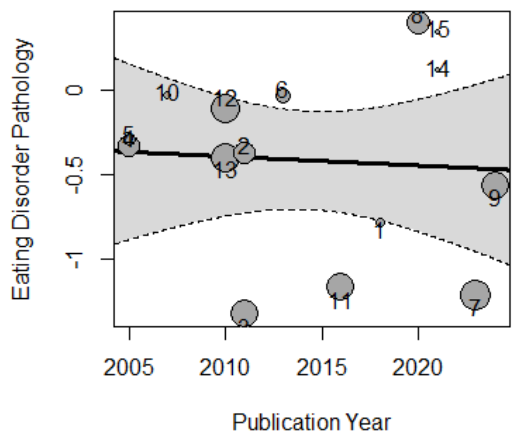

Note. size of circles indicates sample size.

Figure S30. *Scatterplot Depicting Effect Sizes for Binge-Eating Frequency and Publication Year for Studies Investigating Binge-Eating Disorder*

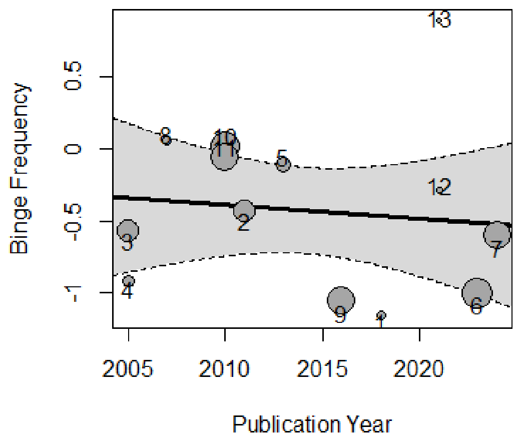

Note. size of circles indicates sample size.

Figure S31. *Scatterplot Depicting Effect Sizes for Eating Disorder Pathology and Treatment Duration for Studies Investigating Mixed Eating Disorders*

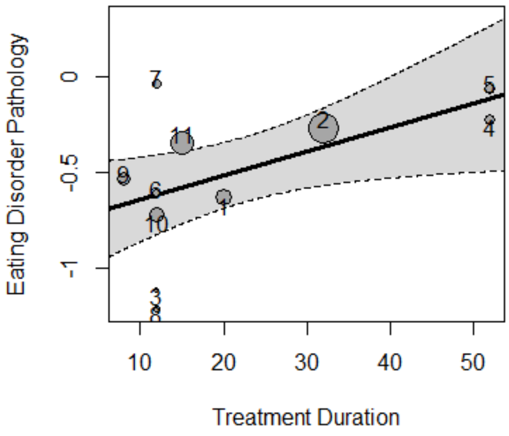

Note. size of circles indicates sample size.

Figure S32. *Scatterplot Depicting Effect Sizes for Binge-Eating Frequency and Treatment Duration for Studies Investigating Mixed Eating Disorders*

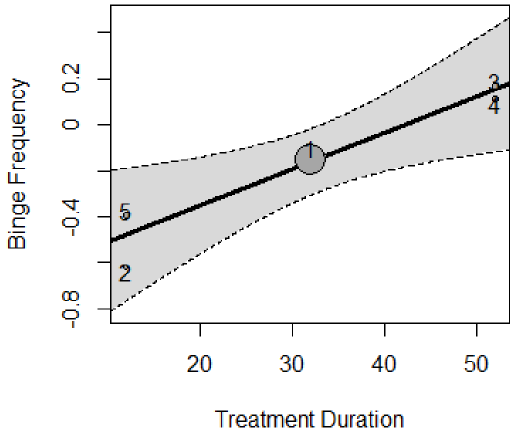

Note. size of circles indicates sample size.

Figure S33. *Scatterplot Depicting Effect Sizes for Eating Disorder Pathology and Baseline Eating Disorder Severity for Studies Investigating Mixed Eating Disorders*

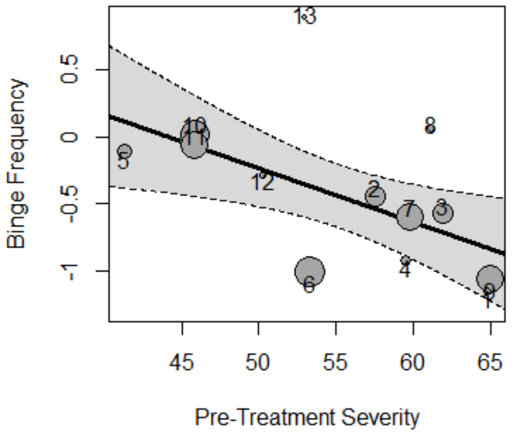

Note. size of circles indicates sample size.

Figure S34. *Scatterplot Depicting Effect Sizes for Binge-Eating Frequency and Baseline Eating Disorder Severity for Studies Investigating Mixed Eating Disorders*

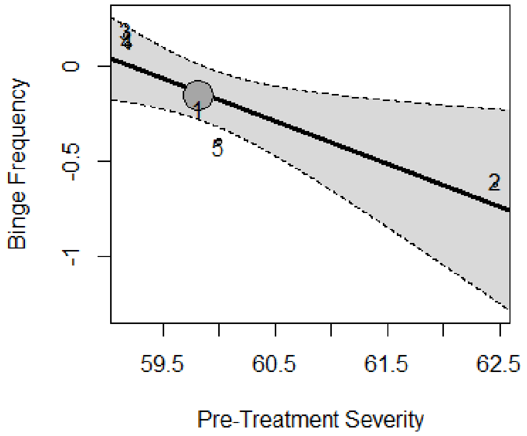

Note. size of circles indicates sample size.

Figure S35. *Scatterplot Depicting Effect Sizes for Eating Disorder Pathology and Publication Year for Studies Investigating Mixed Eating Disorders*

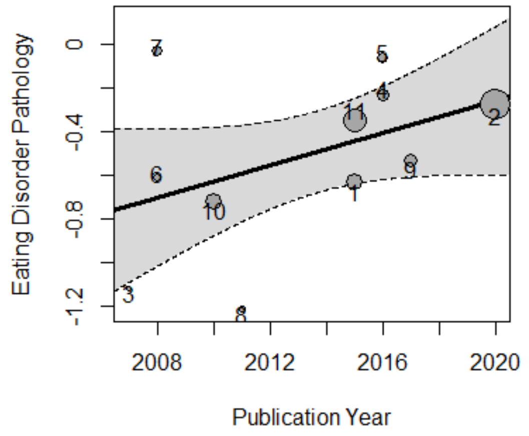

Note. size of circles indicates sample size.

Figure S36. *Scatterplot Depicting Effect Sizes for Binge-Eating Frequency and Publication Year for Studies Investigating Mixed Eating Disorders*

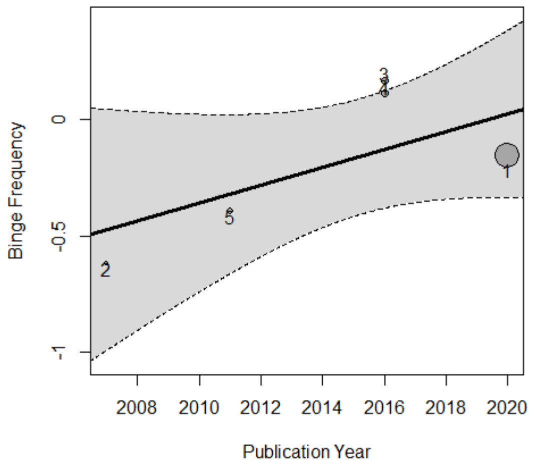

Note. size of circles indicates sample size.

Results of the Sensitivity Analyses

Anorexia Nervosa Studies

Univariate Analysis

Compared to other active treatments, there was no effect in favor of therapist-led CBT for AN for either ED pathology, g = -0.06, 95%-CI [-0.40; 0.28], or BMI, g = 0.08, 95%-CI [-0.26; 0.42], in the univariate analysis.

Intention-to-Treat

All included comparisons used ITT-analyses.

Study Quality

There were c = 3 comparisons reported in k = 2 studies with a quality score of 7 or above. The analysis yielded no effect in favor of therapist-led CBT for AN compared to active treatments for ED pathology, g = -0.04, 95%-CI [-0.68; 0.59], or BMI, g = 0.08, 95%-CI [-0.43; 0.60].

Different Values for ρ

Across different values for ρ, therapist-led CBT for AN was not associated with either less ED pathology or a higher BMI in comparison to active treatments (see Table S5).

Table S5. *Results of Sensitivity Analysis using Different Values of ρ for Studies Investigating Anorexia Nervosa*

ρ	eating disorder pathology			body mass index		
	g	95%-CI	95%-PI	*g*	95%-CI	95%-PI
main analysis						
-0.9	-0.06	[-0.38; 0.27]	[-0.54; 0.43]	0.15	[-0.18; 0.47]	[-0.18; 0.47]
-0.7	-0.06	[-0.38; 0.27]	[-0.54; 0.43]	0.15	[-0.18; 0.47]	[-0.18; 0.47]
-0.5	-0.06	[-0.38; 0.27]	[-0.54; 0.43]	0.15	[-0.18; 0.47]	[-0.18; 0.47]
-0.3	-0.06	[-0.38; 0.27]	[-0.54; 0.43]	0.15	[-0.18; 0.47]	[-0.18; 0.47]
-0.1	-0.06	[-0.38; 0.27]	[-0.54; 0.43]	0.15	[-0.18; 0.47]	[-0.18; 0.47]
0.1	-0.06	[-0.38; 0.27]	[-0.54; 0.43]	0.15	[-0.18; 0.47]	[-0.18; 0.48]
0.3	-0.06	[-0.38; 0.27]	[-0.54; 0.43]	0.15	[-0.18; 0.48]	[-0.18; 0.48]
**0.5**	**-0.06**	**[-0.38; 0.27]**	**[-0.55; 0.43]**	**0.15**	**[-0.18; 0.48]**	**[-0.19; 0.49]**
0.7	-0.06	[-0.39; 0.27]	[-0.56; 0.45]	0.16	[-0.17; 0.49]	[-0.20; 0.52]
0.9	-0.06	[-0.39; 0.28]	[-0.58; 0.47]	0.17	[-0.16; 0.51]	[-0.21; 0.55]

Note. results in bold indicate results reported in the main results section.

Bulimia Nervosa Studies

Univariate Analysis

Across all types of comparison groups and treatment formats, CBT for BN was associated with lower levels of ED pathology, g = -0.42, 95%-CI [-0.71; -0.13] and a lower binge-eating frequency, g = -0.36, 95%-CI [-0.51; -0.21], but not a lower frequency of compensatory behavior, g = -0.22, 95%-CI [-0.46; 0.01], in the univariate analysis.

Intention-to-Treat

Only including ITT analysis, we included c = 7 comparisons reported in k = 6 studies. The analysis yielded differences between CBT for BN and comparison groups for binge-eating frequency, g = -0.34, 95%-CI [-0.63; -0.04], but not for ED pathology, g = -0.32, 95%-CI [-0.80; 0.15], and frequency of compensatory behavior, g = -0.20, 95%-CI [-0.58; 0.18].

Study Quality

There were c = 3 comparisons reported in k = 3 studies with a quality score of 7 or above. The analysis showed an effect in favor of CBT for BN to comparison groups for ED pathology, g = -0.65, 95%-CI [-1.29; -0.01], and binge-eating frequency, g = -0.48, 95%-CI [-0.83; -0.13], but showed no significant differences for frequency of compensatory behavior, g = -0.27, 95%-CI [-0.60; 0.06].

Different Values for ρ

Across different values for ρ, CBT for BN was associated with less ED pathology, a lower binge-eating frequency, and a lower frequency of compensatory behavior compared to comparison groups. In comparison to waitlist, there was an effect in favor of CBT for all outcomes, but there were no significant differences between CBT and active treatments for either outcome. CBT was superior to TAU for binge-eating frequency, but not for ED pathology. When $\rho \leq .1$, there was an effect in favor of CBT for BN compared to TAU for frequency of

compensatory behaviors. Therapist-led CBT was superior to comparison groups for all outcomes, whereas we found superiority for guided self-help CBT only for ED pathology and binge-eating frequency. There was an effect in favor of pure self-help CBT for binge-eating frequency and frequency of compensatory behaviors. Treatment duration did not emerge as a significant moderator. Baseline eating disorder severity did not moderate the size of the effect. Publication year was not a significant moderator for either outcome (see Table S6).

Table S6. *Results of Sensitivity Analysis using Different Values of ρ for Studies Investigating Bulimia Nervosa*

		eating disorder pathology			binge-eating frequency			frequency of compensatory behaviors		
main analysis	ρ	*g*	95%-CI	95%-PI	*g*	95%-CI	95%-PI	*g*	95%-CI	95%-PI
	-0.9	-0.42	[-0.68; -0.16]	[-1.37; 0.53]	-0.35	[-0.50; -0.20]	[-0.50; -0.20]	-0.32	[-0.47; -0.16]	[-0.47; -0.16]
	-0.7	-0.42	[-0.68; -0.16]	[-1.37; 0.53]	-0.35	[-0.50; -0.20]	[-0.50; -0.20]	-0.32	[-0.47; -0.16]	[-0.47; -0.16]
	-0.5	-0.42	[-0.68; -0.16]	[-1.37; 0.53]	-0.35	[-0.50; -0.20]	[-0.50; -0.20]	-0.32	[-0.47; -0.16]	[-0.47; -0.16]
	-0.3	-0.42	[-0.68; -0.16]	[-1.37; 0.53]	-0.35	[-0.50; -0.20]	[-0.50; -0.20]	-0.32	[-0.47; -0.16]	[-0.47; -0.16]
	-0.1	-0.42	[-0.68; -0.16]	[-1.37; 0.53]	-0.35	[-0.50; -0.20]	[-0.50; -0.20]	-0.32	[-0.47; -0.16]	[-0.47; -0.16]
	0.1	-0.42	[-0.68; -0.16]	[-1.37; 0.53]	-0.35	[-0.50; -0.20]	[-0.50; -0.20]	-0.32	[-0.47; -0.16]	[-0.47; -0.16]
	0.3	-0.42	[-0.68; -0.16]	[-1.37; 0.53]	-0.35	[-0.50; -0.20]	[-0.52; -0.18]	-0.31	[-0.47; -0.16]	[-0.49; -0.13]
	**0.5**	**-0.42**	**[-0.68; -0.16]**	**[-1.38; 0.55]**	**-0.34**	**[-0.50; -0.19]**	**[-0.55; -0.14]**	**-0.31**	**[-0.47; -0.15]**	**[-0.53; -0.09]**
	0.7	-0.42	[-0.68; -0.15]	[-1.40; 0.57]	-0.34	[-0.50; -0.19]	[-0.59; -0.10]	-0.31	[-0.47; -0.14]	[-0.56; -0.05]
	0.9	-0.42	[-0.69; -0.14]	[-1.44; 0.61]	-0.34	[-0.50; -0.18]	[-0.63; -0.06]	-0.30	[-0.47; -0.14]	[-0.58; -0.03]

		eating disorder pathology			binge-eating frequency			frequency of compensatory behaviors		
type of comparison group	ρ	*g*	95%-CI	95%-PI	*g*	95%-CI	95%-PI	*g*	95%-CI	95%-PI
waitlist	-0.9	-0.77	[-1.15; -0.40]	[-1.90; 0.36]	-0.60	[-0.84; -0.35]	[-0.84; -0.35]	-0.67	[-0.95; -0.40]	[-0.95; -0.40]
	-0.7	-0.77	[-1.15; -0.40]	[-1.90; 0.36]	-0.59	[-0.84; -0.35]	[-0.84; -0.35]	-0.67	[-0.94; -0.40]	[-0.95; -0.40]
	-0.5	-0.77	[-1.15; -0.40]	[-1.90; 0.36]	-0.59	[-0.84; -0.35]	[-0.84; -0.35]	-0.67	[-0.94; -0.40]	[-0.95; -0.40]
	-0.3	-0.77	[-1.15; -0.40]	[-1.90; 0.36]	-0.59	[-0.84; -0.35]	[-0.84; -0.35]	-0.67	[-0.94; -0.40]	[-0.95; -0.40]
	-0.1	-0.77	[-1.15; -0.40]	[-1.90; 0.36]	-0.59	[-0.84; -0.35]	[-0.84; -0.35]	-0.67	[-0.94; -0.40]	[-0.95; -0.40]
	0.1	-0.77	[-1.15; -0.40]	[-1.90; 0.36]	-0.59	[-0.84; -0.35]	[-0.84; -0.35]	-0.67	[-0.94; -0.40]	[-0.95; -0.39]
	0.3	-0.78	[-1.15; -0.40]	[-1.91; 0.36]	-0.59	[-0.84; -0.35]	[-0.84; -0.35]	-0.67	[-0.94; -0.39]	[-0.96; -0.38]
	**0.5**	**-0.78**	**[-1.16; -0.40]**	**[-1.92; 0.36]**	**-0.59**	**[-0.84; -0.35]**	**[-0.84; -0.34]**	**-0.66**	**[-0.94; -0.38]**	**[-0.96; -0.36]**
	0.7	-0.78	[-1.17; -0.40]	[-1.93; 0.37]	-0.59	[-0.83; -0.34]	[-0.84; -0.33]	-0.65	[-0.92; -0.37]	[-0.96; -0.34]
	0.9	-0.79	[-1.17; -0.40]	[-1.95; 0.38]	-0.58	[-0.83; -0.34]	[-0.85; -0.31]	-0.64	[-0.91; -0.36]	[-0.95; -0.32]
active treatment	-0.9	-0.20	[-0.53; 0.14]	[-1.31; 0.92]	-0.16	[-0.36; 0.05]	[-0.36; 0.05]	-0.14	[-0.35; 0.07]	[-0.35; 0.07]
	-0.7	-0.20	[-0.53; 0.14]	[-1.31; 0.92]	-0.16	[-0.36; 0.05]	[-0.36; 0.05]	-0.14	[-0.35; 0.07]	[-0.35; 0.07]
	-0.5	-0.20	[-0.53; 0.14]	[-1.31; 0.92]	-0.16	[-0.36; 0.05]	[-0.36; 0.05]	-0.14	[-0.35; 0.07]	[-0.35; 0.07]

		eating disorder pathology			binge-eating frequency			frequency of compensatory behaviors		
	-0.3	-0.20	[-0.53; 0.14]	[-1.31; 0.92]	-0.16	[-0.36; 0.05]	[-0.36; 0.05]	-0.14	[-0.35; 0.07]	[-0.35; 0.07]
	-0.1	-0.20	[-0.53; 0.14]	[-1.31; 0.92]	-0.16	[-0.36; 0.05]	[-0.36; 0.05]	-0.14	[-0.35; 0.07]	[-0.35; 0.07]
	0.1	-0.20	[-0.53; 0.14]	[-1.31; 0.92]	-0.16	[-0.36; 0.05]	[-0.36; 0.05]	-0.14	[-0.35; 0.07]	[-0.35; 0.07]
	0.3	-0.20	[-0.53; 0.14]	[-1.32; 0.92]	-0.15	[-0.36; 0.05]	[-0.36; 0.05]	-0.14	[-0.35; 0.07]	[-0.36; 0.09]
	**0.5**	**-0.20**	**[-0.53; 0.14]**	**[-1.32; 0.93]**	**-0.15**	**[-0.36; 0.05]**	**[-0.36; 0.06]**	**-0.14**	**[-0.35; 0.07]**	**[-0.38; 0.10]**
	0.7	-0.19	[-0.53; 0.14]	[-1.32; 0.94]	-0.15	[-0.36; 0.05]	[-0.37; 0.07]	-0.14	[-0.35; 0.07]	[-0.39; 0.12]
	0.9	-0.19	[-0.54; 0.15]	[-1.35; 0.96]	-0.15	[-0.36; 0.05]	[-0.39; 0.08]	-0.13	[-0.34; 0.07]	[-0.39; 0.13]
treat. as usual	-0.9	-0.28	[-1.41; 0.84]	[-1.83; 1.26]	-0.46	[-0.81; -0.10]	[-0.81; -0.10]	-0.37	[-0.72; -0.02]	[-0.72; -0.01]
	-0.7	-0.28	[-1.41; 0.84]	[-1.83; 1.26]	-0.46	[-0.81; -0.10]	[-0.81; -0.10]	-0.37	[-0.72; -0.02]	[-0.72; -0.01]
	-0.5	-0.28	[-1.41; 0.84]	[-1.83; 1.26]	-0.46	[-0.81; -0.10]	[-0.81; -0.10]	-0.37	[-0.72; -0.02]	[-0.72; -0.01]
	-0.3	-0.28	[-1.41; 0.84]	[-1.83; 1.26]	-0.46	[-0.81; -0.10]	[-0.81; -0.10]	-0.37	[-0.72; -0.02]	[-0.72; -0.01]
	-0.1	-0.28	[-1.41; 0.84]	[-1.83; 1.26]	-0.46	[-0.81; -0.10]	[-0.81; -0.10]	-0.37	[-0.72; -0.02]	[-0.72; -0.01]
	0.1	-0.28	[-1.41; 0.84]	[-1.83; 1.26]	-0.46	[-0.81; -0.10]	[-0.81; -0.10]	-0.37	[-0.72; -0.01]	[-0.72; -0.01]
	0.3	-0.28	[-1.41; 0.84]	[-1.84; 1.27]	-0.46	[-0.81; -0.10]	[-0.81; -0.10]	-0.37	[-0.73; -0.01]	[-0.74; 0.00]
	**0.5**	**-0.28**	**[-1.42; 0.85]**	**[-1.85; 1.28]**	**-0.46**	**[-0.81; -0.10]**	**[-0.82; -0.10]**	**-0.37**	**[-0.74; 0.00]**	**[-0.75; 0.02]**

		eating disorder pathology			binge-eating frequency			frequency of compensatory behaviors		
	0.7	-0.28	[-1.42; 0.86]	[-1.86; 1.29]	-0.46	[-0.81; -0.10]	[-0.82; -0.09]	-0.37	[-0.74; 0.01]	[-0.77; 0.04]
	0.9	-0.28	[-1.44; 0.87]	[-1.88; 1.31]	-0.46	[-0.82; -0.09]	[-0.84; -0.07]	-0.37	[-0.75; 0.02]	[-0.78; 0.05]
treatment format	ρ	0.9	95%-CI	95%-PI	*g*	95%-CI	95%-PI	*g*	95%-CI	95%-PI
therapist-led	-0.9	0.45	[-0.78; -0.12]	[-1.43; 0.53]	-0.21	[-0.41; -0.01]	[-0.41; -0.01]	-0.23	[-0.42; -0.04]	[-0.42; -0.04]
	-0.7	0.45	[-0.78; -0.12]	[-1.43; 0.53]	-0.21	[-0.41; -0.01]	[-0.41; -0.01]	-0.23	[-0.42; -0.04]	[-0.42; -0.04]
	-0.5	0.45	[-0.78; -0.12]	[-1.43; 0.53]	-0.21	[-0.41; -0.01]	[-0.41; -0.01]	-0.23	[-0.42; -0.04]	[-0.42; -0.04]
	-0.3	0.45	[-0.78; -0.12]	[-1.43; 0.53]	-0.21	[-0.41; -0.01]	[-0.41; -0.01]	-0.23	[-0.42; -0.04]	[-0.42; -0.04]
	-0.1	0.45	[-0.78; -0.12]	[-1.43; 0.53]	-0.21	[-0.41; -0.01]	[-0.41; -0.01]	-0.23	[-0.42; -0.04]	[-0.42; -0.04]
	0.1	0.45	[-0.78; -0.12]	[-1.43; 0.54]	-0.21	[-0.41; -0.01]	[-0.41; -0.01]	-0.23	[-0.42; -0.04]	[-0.45; -0.01]
	0.3	0.45	[-0.78; -0.12]	[-1.44; 0.54]	-0.21	[-0.41; -0.01]	[-0.41; -0.01]	-0.24	[-0.44; -0.04]	[-0.50; 0.03]
	**0.5**	**0.45**	**[-0.79; -0.11]**	**[-1.46; 0.55]**	**-0.21**	**[-0.41; -0.01]**	**[-0.42; -0.004]**	**-0.24**	**[-0.45; -0.04]**	**[-0.54; 0.05]**
	0.7	0.45	[-0.79; -0.11]	[-1.48; 0.57]	-0.21	[-0.42; -0.01]	[-0.43; 0.002]	-0.25	[-0.46; -0.04]	[-0.56; 0.06]
	0.9	0.45	[-0.80; -0.10]	[-1.51; 0.60]	-0.22	[-0.42; -0.01]	[-0.44; 0.01]	-0.25	[-0.46; -0.05]	[-0.57; 0.06]
guided self-help	-0.9	-0.58	[-1.09; -0.08]	[-1.64; 0.47]	-0.66	[-0.94; -0.37]	[-0.94; -0.37]	-0.27	[-0.67; 0.12]	[-0.67; 0.12]
	-0.7	-0.58	[-1.09; -0.08]	[-1.64; 0.47]	-0.66	[-0.94; -0.37]	[-0.94; -0.37]	-0.27	[-0.67; 0.12]	[-0.67; 0.12]

		eating disorder pathology			binge-eating frequency			frequency of compensatory behaviors		
	-0.5	-0.58	[-1.09; -0.08]	[-1.64; 0.47]	-0.66	[-0.94; -0.37]	[-0.94; -0.37]	-0.27	[-0.67; 0.12]	[-0.67; 0.12]
	-0.3	-0.58	[-1.09; -0.08]	[-1.64; 0.47]	-0.66	[-0.94; -0.37]	[-0.94; -0.37]	-0.27	[-0.67; 0.12]	[-0.67; 0.12]
	-0.1	-0.58	[-1.09; -0.08]	[-1.64; 0.47]	-0.66	[-0.94; -0.37]	[-0.94; -0.37]	-0.27	[-0.67; 0.12]	[-0.67; 0.12]
	0.1	-0.58	[-1.09; -0.08]	[-1.64; 0.47]	-0.66	[-0.94; -0.37]	[-0.94; -0.37]	-0.26	[-0.67; 0.14]	[-0.68; 0.15]
	0.3	-0.58	[-1.09; -0.07]	[-1.64; 0.49]	-0.65	[-0.94; -0.37]	[-0.94; -0.36]	-0.24	[-0.65; 0.17]	[-0.69; 0.21]
	0.5	**-0.57**	**[-1.09; -0.06]**	**[-1.65; 0.50]**	**-0.65**	**[-0.94; -0.36]**	**[-0.94; -0.36]**	**-0.22**	**[-0.63; 0.20]**	**[-0.68; 0.25]**
	0.7	-0.57	[-1.09; -0.05]	[-1.67; 0.53]	-0.64	[-0.93; -0.35]	[-0.94; -0.34]	-0.20	[-0.61; 0.22]	[-0.67; 0.28]
	0.9	-0.57	[-1.10; -0.04]	[-1.70; 0.55]	-0.63	[-0.92; -0.34]	[-0.94; -0.32]	-0.18	[-0.59; 0.23]	[-0.65; 0.29]
pure self-help	-0.9	-0.17	[-0.63; 0.28]	[-1.20; 0.86]	-0.40	[-0.67; -0.14]	[-0.67; -0.14]	-0.45	[-0.72; -0.17]	[-0.72; -0.17]
	-0.7	-0.17	[-0.63; 0.28]	[-1.20; 0.86]	-0.40	[-0.67; -0.14]	[-0.67; -0.14]	-0.45	[-0.72; -0.17]	[-0.72; -0.17]
	-0.5	-0.17	[-0.63; 0.28]	[-1.20; 0.86]	-0.40	[-0.67; -0.14]	[-0.67; -0.14]	-0.45	[-0.72; -0.17]	[-0.72; -0.17]
	-0.3	-0.17	[-0.63; 0.28]	[-1.20; 0.86]	-0.40	[-0.67; -0.14]	[-0.67; -0.14]	-0.45	[-0.72; -0.17]	[-0.72; -0.17]
	-0.1	-0.17	[-0.63; 0.28]	[-1.20; 0.86]	-0.40	[-0.67; -0.14]	[-0.67; -0.14]	-0.45	[-0.72; -0.17]	[-0.72; -0.17]
	0.1	-0.18	[-0.63; 0.28]	[-1.21; 0.86]	-0.40	[-0.67; -0.14]	[-0.67; -0.14]	-0.45	[-0.73; -0.16]	[-0.76; -0.14]
	0.3	-0.18	[-0.64; 0.28]	[-1.22; 0.86]	-0.40	[-0.67; -0.13]	[-0.67; -0.13]	-0.44	[-0.74; -0.14]	[-0.79; -0.09]

		eating disorder pathology			binge-eating frequency			frequency of compensatory behaviors		
	**0.5**	**-0.18**	**[-0.65; 0.29]**	**[-1.24; 0.88]**	**-0.40**	**[-0.67; -0.13]**	**[-0.67; -0.13]**	**-0.42**	**[-0.73; -0.12]**	**[-0.79; -0.06]**
	0.7	-0.18	[-0.65; 0.29]	[-1.26; 0.90]	-0.39	[-0.66; -0.12]	[-0.67; -0.11]	-0.41	[-0.71; -0.10]	[-0.79; -0.03]
	0.9	-0.18	[-0.66; 0.30]	[-1.29; 0.92]	-0.39	[-0.66; -0.11]	[-0.67; -0.10]	-0.39	[-0.69; -0.10]	[-0.77; -0.01]
treatment duration	ρ	*b*	*z*	*p*	*b*	*z*	*p*	*b*	*z*	*p*
	-0.9	0.01	0.47	.640	0.04	1.72	.086	0.04	1.94	.053
	-0.7	0.01	0.47	.640	0.04	1.72	.086	0.04	1.94	.053
	-0.5	0.01	0.47	.640	0.04	1.72	.086	0.04	1.94	.053
	-0.3	0.01	0.47	.640	0.04	1.72	.086	0.04	1.94	.053
	-0.1	0.01	0.47	.640	0.04	1.72	.086	0.04	1.94	.053
	0.1	0.01	0.47	.640	0.04	1.71	.086	0.04	1.94	.053
	0.3	0.01	0.47	.640	0.03	1.69	.091	0.04	1.91	.056
	**0.5**	**0.01**	**0.47**	**.641**	**0.03**	**1.63**	**.103**	**0.04**	**1.87**	**.061**
	0.7	0.01	0.47	.642	0.03	1.53	.125	0.04	1.80	.071
	0.9	0.01	0.46	.645	0.03	1.39	.164	0.04	1.72	.086

		eating disorder pathology			binge-eating frequency			frequency of compensatory behaviors		
baseline severity	ρ	*b*	*z*	*p*	*b*	*z*	*p*	*b*	*z*	*p*
	-0.9	0.004	0.45	.655	-0.01	-1.29	.195	0.002	0.33	.743
	-0.7	0.004	0.45	.655	-0.01	-1.29	.195	0.002	0.33	.743
	-0.5	0.004	0.45	.655	-0.01	-1.29	.195	0.002	0.33	.743
	-0.3	0.004	0.45	.655	-0.01	-1.29	.195	0.002	0.33	.743
	-0.1	0.004	0.45	.655	-0.01	-1.29	.195	0.002	0.33	.743
	0.1	0.004	0.45	.654	-0.01	-1.29	.196	0.002	0.34	.734
	0.3	0.004	0.45	.651	-0.01	-1.27	.204	0.003	0.40	.692
	**0.5**	**0.004**	**0.45**	**.651**	**-0.01**	**-1.22**	**.223**	**0.003**	**0.46**	**.647**
	0.7	0.004	0.44	.657	-0.01	-1.15	.249	0.004	0.51	.608
	0.9	0.004	0.41	.681	-0.01	-1.09	.277	0.004	0.55	.583
publication year	ρ	*b*	*z*	*p*	*b*	*z*	*p*	*b*	*z*	*p*
	-0.9	0.02	1.67	.095	-0.003	-0.50	.617	0.004	0.65	.519
	-0.7	0.02	1.67	.095	-0.003	-0.50	.617	0.004	0.65	.519
	-0.5	0.02	1.67	.095	-0.003	-0.50	.617	0.004	0.65	.519

	eating disorder pathology			binge-eating frequency			frequency of compensatory behaviors		
-0.3	0.02	1.67	.095	-0.003	-0.50	.617	0.004	0.65	.519
-0.1	0.02	1.67	.095	-0.003	-0.50	.617	0.004	0.65	.519
0.1	0.02	1.67	.095	-0.003	-0.50	.620	0.004	0.65	.518
0.3	0.02	1.68	.094	-0.003	-0.45	.650	0.004	0.65	.513
**0.5**	**0.02**	**1.68**	**.094**	**-0.002**	**-0.38**	**.707**	**0.004**	**0.66**	**.506**
0.7	0.02	1.67	.096	-0.002	-0.28	.776	0.004	0.67	.503
0.9	0.02	1.63	.102	-0.001	-0.17	.861	0.004	0.66	.507

Note. results in bold indicate results reported in the main results section.

Binge-Eating Disorder Studies

Univariate Analysis

Across all types of comparison groups and self-help treatment formats, CBT for BED was associated with lower levels of ED pathology, $g = -0.48$, 95%-CI [-0.81; -0.15], and a lower binge-eating frequency, $g = -0.50$, 95%-CI [-0.80; -0.20].

Intention-to-Treat

Only including ITT analysis, we included $c = 9$ comparisons reported in $k = 7$ studies. The analysis yielded significant differences between CBT for BED and comparison groups for ED pathology, $g = -0.63$, 95%-CI [-0.99; -0.27], and binge-eating frequency, $g = -0.59$, 95%-CI [-0.93; -0.25]. Baseline eating disorder severity was a significant moderator for ED pathology, $b = -0.04$, $p = .010$, and binge-eating frequency, $b = -0.05$, $p < .001$.

Study Quality

There were $c = 6$ comparisons reported in $k = 4$ studies with a quality score of 7 or above. The analysis showed an effect in favor of CBT for BN compared to comparison groups for ED pathology, $g = -0.75$, 95%-CI [-1.26; -0.25], and binge-eating frequency, $g = -0.66$, 95%-CI [-1.15; -0.17].

Different Values for ρ

Across different values for ρ, CBT for BED was superior to comparison groups for both ED pathology and binging frequency. While CBT was associated with less ED pathology and a lower binge-eating frequency compared to waitlist, there were no differences between CBT and other active treatments for either outcome. There was an effect in favor of CBT compared to TAU for ED pathology, but nor binge-eating frequency. The effect for ED pathology was not significant when $\rho = .9$. Guided self-help CBT for BED was associated with less ED pathology and a lower binge-eating frequency than comparison groups, while there were no differences

between pure self-help CBT and comparison groups. Treatment duration was not a significant moderator for either outcome. Baseline eating disorder severity consistently moderate the magnitude of the effect sizes for both ED pathology and binging frequency. A larger baseline severity was associated with larger effects in favor of CBT. Publication year did not emerge as a moderator for either outcome. (see Table S7).

Table S7. *Results of Sensitivity Analysis using Different Values of ρ for Studies Investigating Binge-Eating Disorder*

		eating disorder pathology			binge-eating frequency		
main analysis	ρ	*g*	95%-CI	95%-PI	*g*	95%-CI	95%-PI
	-0.9	-0.47	[-0.76; -0.18]	[-1.45; 0.52]	-0.47	[-0.72; -0.22]	[-1.22; 0.28]
	-0.7	-0.47	[-0.76; -0.18]	[-1.45; 0.52]	-0.47	[-0.72; -0.22]	[-1.22; 0.28]
	-0.5	-0.47	[-0.76; -0.18]	[-1.45; 0.52]	-0.47	[-0.72; -0.22]	[-1.22; 0.28]
	-0.3	-0.47	[-0.76; -0.18]	[-1.45; 0.52]	-0.47	[-0.72; -0.22]	[-1.22; 0.28]
	-0.1	-0.48	[-0.79; -0.17]	[-1.50; 0.54]	-0.47	[-0.78; -0.22]	[-1.33; 0.33]
	0.1	-0.48	[-0.78; -0.18]	[-1.48; 0.52]	-0.50	[-0.78; -0.22]	[-1.32; 0.32]
	0.3	-0.48	[-0.78; -0.18]	[-1.47; 0.50]	-0.50	[-0.78; -0.22]	[-1.31; 0.31]
	**0.5**	**-0.48**	**[-0.78; -0.18]**	**[-1.45; 0.49]**	**-0.50**	**[-0.77; -0.22]**	**[-1.31; 0.32]**
	0.7	-0.48	[-0.78; -0.18]	[-1.45; 0.49]	-0.49	[-0.77; -0.21]	[-1.33; 0.35]
	0.9	-0.48	[-0.79; -0.17]	[-1.50; 0.54]	-0.48	[-0.78; -0.19]	[-1.41; 0.44]
type of comparison group	ρ	*g*	95%-CI	95%-PI	*g*	95%-CI	95%-PI
waitlist	-0.9	-0.61	[-0.99; -0.24]	[-1.57; 0.34]	-0.60	[-0.90; -0.30]	[-1.31; 0.11]
	-0.7	-0.61	[-0.99; -0.24]	[-1.57; 0.34]	-0.60	[-0.90; -0.30]	[-1.31; 0.11]

		eating disorder pathology			binge-eating frequency		
	-0.5	-0.61	[-0.99; -0.24]	[-1.57; 0.34]	-0.60	[-0.90; -0.30]	[-1.31; 0.11]
	-0.3	-0.62	[-1.00; -0.23]	[-1.60; 0.37]	-0.62	[-0.90; -0.27]	[-1.47; 0.24]
	-0.1	-0.62	[-1.01; -0.23]	[-1.61; 0.38]	-0.61	[-0.96; -0.26]	[-1.49; 0.26]
	0.1	-0.61	[-1.00; -0.23]	[-1.59; 0.37]	-0.61	[-0.96; -0.26]	[-1.48; 0.26]
	0.3	-0.61	[-0.99; -0.23]	[-1.58; 0.36]	-0.61	[-0.96; -0.26]	[-1.47; 0.26]
	**0.5**	**-0.61**	**[-0.99; -0.22]**	**[-1.57; 0.36]**	**-0.60**	**[-0.95; -0.25]**	**[-1.48; 0.27]**
	0.7	-0.60	[-0.99; -0.22]	[-1.58; 0.37]	-0.59	[-0.96; -0.23]	[-1.50; 0.31]
	0.9	-0.60	[-1.00; -0.20]	[-1.64; 0.44]	-0.58	[-0.98; -0.19]	[-1.58; 0.42]
active treatment	-0.9	-0.08	[-0.58; 0.43]	[-1.09; 0.93]	-0.30	[-0.71; 0.12]	[-1.06; 0.47]
	-0.7	-0.08	[-0.58; 0.43]	[-1.09; 0.93]	-0.30	[-0.71; 0.12]	[-1.06; 0.47]
	-0.5	-0.08	[-0.58; 0.43]	[-1.09; 0.93]	-0.30	[-0.71; 0.12]	[-1.06; 0.47]
	-0.3	-0.06	[-0.61; 0.48]	[-1.12; 0.93]	-0.35	[-0.91; 0.21]	[-1.31; 0.62]
	-0.1	-0.06	[-0.62; 0.50]	[-1.13; 0.99]	-0.33	[-0.92; 0.26]	[-1.32; 0.66]
	0.1	-0.06	[-0.61; 0.49]	[-1.12; 1.01]	-0.30	[-0.89; 0.29]	[-1.29; 0.68]
	0.3	-0.06	[-0.61; 0.49]	[-1.11; 0.99]	-0.27	[-0.86; 0.31]	[-1.26; 0.71]

			eating disorder pathology			binge-eating frequency	
	**0.5**	**-0.06**	**[-0.61; 0.49]**	**[-1.11; 0.98]**	**-0.24**	**[-0.83; 0.35]**	**[-1.23; 0.75]**
	0.7	-0.07	[-0.62; 0.49]	[-1.12; 0.99]	-0.20	[-0.79; 0.39]	[-1.22; 0.82]
	0.9	-0.07	[-0.66; 0.52]	[-1.20; 1.05]	-0.14	[-0.75; 0.47]	[-1.25; 0.96]
treatment as usual	-0.9	-0.74	[-1.45; -0.04]	[-1.87; 0.38]	-0.22	[-1.07; 0.64]	[-1.29; 0.85]
	-0.7	-0.74	[-1.45; -0.04]	[-1.87; 0.38]	-0.22	[-1.07; 0.64]	[-1.29; 0.85]
	-0.5	-0.74	[-1.45; -0.04]	[-1.87; 0.38]	-0.22	[-1.07; 0.64]	[-1.29; 0.85]
	-0.3	-0.74	[-1.46; -0.02]	[-1.90; 0.42]	-0.12	[-1.09; 0.84]	[-1.37; 1.12]
	-0.1	-0.74	[-1.46; -0.02]	[-1.91; 0.43]	-0.18	[-1.15; 0.80]	[-1.44; 1.08]
	0.1	-0.74	[-1.46; -0.02]	[-1.89; 0.41]	-0.25	[-1.21; 0.71]	[-1.49; 1.00]
	0.3	-0.74	[-1.45; -0.03]	[-1.88; 0.40]	-0.33	[-1.27; 0.62]	[-1.56; 0.90]
	**0.5**	**-0.74**	**[-1.45; -0.04]**	**[-1.88; 0.39]**	**-0.41**	**[-1.34; 0.51]**	**[-1.64; 0.81]**
	0.7	-0.74	[-1.46; -0.03]	[-1.89; 0.41]	-0.51	[-1.41; 0.40]	[-1.74; 0.72]
	0.9	-0.74	[-1.49; 0.02]	[-1.95; 0.48]	-0.63	[-1.53; 0.27]	[-1.91; 0.66]

		eating disorder pathology			binge-eating frequency		
treatment format	ρ	*g*	95%-CI	95%-PI	*g*	95%-CI	95%-PI
guided self-help	-0.9	-0.53	[-0.87; -0.18]	[-1.56; 0.51]	-0.53	[-0.81; -0.24]	[-1.31; 0.26]
	-0.7	-0.53	[-0.87; -0.18]	[-1.56; 0.51]	-0.53	[-0.81; -0.24]	[-1.31; 0.26]
	-0.5	-0.53	[-0.87; -0.18]	[-1.56; 0.51]	-0.53	[-0.81; -0.24]	[-1.31; 0.26]
	-0.3	-0.54	[-0.90; -0.18]	[-1.62; 0.54]	-0.57	[-0.91; -0.23]	[-1.46; 0.32]
	-0.1	-0.55	[-0.91; -0.18]	[-1.62; 0.53]	-0.58	[-0.93; -0.24]	[-1.47; 0.30]
	0.1	-0.55	[-0.91; -0.19]	[-1.60; 0.50]	-0.58	[-0.92; -0.24]	[-1.45; 0.29]
	0.3	-0.55	[-0.90; -0.20]	[-1.58; 0.48]	-0.58	[-0.92; -0.25]	[-1.44; 0.28]
	**0.5**	**-0.55**	**[-0.90; -0.20]**	**[-1.57; 0.46]**	**-0.58**	**[-0.91; -0.24]**	**[-1.44; 0.29]**
	0.7	-0.55	[-0.90; -0.21]	[-1.57; 0.46]	-0.57	[-0.91; -0.23]	[-1.46; 0.32]
	0.9	-0.56	[-0.92; -0.20]	[-1.62; 0.50]	-0.56	[-0.91; -0.20]	[-1.53; 0.42]
pure self-help	-0.9	-0.25	[-0.90; 0.39]	[-1.43; 0.92]	-0.27	[-0.79; 0.24]	[-1.17; 0.62]
	-0.7	-0.25	[-0.90; 0.39]	[-1.43; 0.92]	-0.27	[-0.79; 0.24]	[-1.17; 0.62]
	-0.5	-0.25	[-0.90; 0.39]	[-1.43; 0.92]	-0.27	[-0.79; 0.24]	[-1.17; 0.62]
	-0.3	-0.26	[-0.92; 0.41]	[-1.47; 0.92]	-0.27	[-0.84; 0.29]	[-1.27; 0.72]

		eating disorder pathology			binge-eating frequency		
	-0.1	-0.25	[-0.91; 0.41]	[-1.46; 0.92]	-0.27	[-0.83; 0.29]	[-1.26; 0.72]
	0.1	-0.25	[-0.90; 0.40]	[-1.43; 0.92]	-0.27	[-0.82; 0.29]	[-1.24; 0.71]
	0.3	-0.24	[-0.88; 0.39]	[-1.40; 0.92]	-0.26	[-0.81; 0.29]	[-1.23; 0.70]
	**0.5**	**-0.24**	**[-0.87; 0.39]**	**[-1.38; 0.92]**	**-0.26**	**[-0.81; 0.29]**	**[-1.23; 0.71]**
	0.7	-0.24	[-0.87; 0.39]	[-1.38; 0.92]	-0.26	[-0.82; 0.31]	[-1.26; 0.74]
	0.9	-0.23	[-0.88; 0.42]	[-1.42; 0.92]	-0.25	[-0.86; 0.36]	[-1.35; 0.84]
treatment duration	ρ	*b*	*z*	*p*	*b*	*z*	*p*
	-0.9	0.02	0.53	.594	0.02	0.85	.396
	-0.7	0.02	0.53	.594	0.02	0.85	.396
	-0.5	0.02	0.53	.594	0.02	0.85	.396
	-0.3	0.02	0.53	.594	0.02	0.85	.396
	-0.1	0.02	0.51	.607	0.01	0.48	.633
	0.1	0.02	0.50	.613	0.01	0.51	.607
	0.3	0.02	0.49	.621	0.02	0.57	.572
	**0.5**	**0.02**	**0.48**	**.631**	**0.02**	**0.62**	**.536**

		eating disorder pathology			binge-eating frequency		
	0.7	0.01	0.46	.649	0.02	0.67	.502
	0.9	0.01	0.40	.690	0.02	0.71	.479
baseline severity	ρ	*b*	*z*	*p*	*b*	*z*	*p*
	-0.9	-0.04	-3.08	.002	-0.04	-2.95	.003
	-0.7	-0.04	-3.08	.002	-0.04	-2.95	.003
	-0.5	-0.04	-3.08	.002	-0.04	-2.95	.003
	-0.3	-0.04	-3.08	.002	-0.04	-2.95	.003
	-0.1	-0.04	-3.08	.002	-0.04	-2.95	.003
	0.1	-0.04	-3.05	.002	-0.04	-2.69	.007
	0.3	-0.04	-3.02	.003	-0.04	-2.61	.009
	**0.5**	**-0.04**	**-2.97**	**.003**	**-0.04**	**-2.68**	**.007**
	0.7	-0.04	-2.88	.004	-0.04	-2.81	.005
	0.9	-0.04	-2.68	.007	-0.04	-2.99	.003

		eating disorder pathology			binge-eating frequency		
publication year	ρ	*b*	*z*	*p*	*b*	*z*	*p*
	-0.9	-0.01	-0.44	.662	-0.02	-0.88	.378
	-0.7	-0.01	-0.44	.662	-0.02	-0.88	.378
	-0.5	-0.01	-0.44	.662	-0.02	-0.88	.378
	-0.3	-0.01	-0.39	.697	-0.02	-1.03	.303
	-0.1	-0.01	-0.39	.699	-0.02	-0.96	.336
	0.1	-0.01	-0.39	.698	-0.02	-0.87	.383
	0.3	-0.01	-0.39	.697	-0.02	-0.76	.445
	**0.5**	**-0.01**	**-0.39**	**.697**	**-0.01**	**-0.63**	**.526**
	0.7	-0.01	-0.39	.700	-0.01	-0.48	.635
	0.9	-0.01	-0.37	.712	-0.01	-0.26	.791

Note. results in bold indicate results reported in the main results section.

Mixed Eating Disorders Studies

Univariate Analysis

Across all types of comparison groups and treatment formats, CBT was associated with lower levels of ED pathology, *g* = -0.54, 95%-CI [-0.76; -0.32], but not a lower binge-eating frequency, *g* = -0.21, 95%-CI [-0.47; 0.06], in samples with mixed ED diagnoses in the univariate analysis.

Intention-to-Treat

Only including ITT analysis, we included *c* = 5 comparisons reported in *k* = 3 studies. The analysis yielded significant differences between CBT and comparison groups for ED pathology, *g* = -0.31, 95%-CI [-0.58; -0.04], but not for binge-eating frequency, *g* = 0.08, 95%-CI [-0.35; 0.50], in samples with mixed ED diagnoses.

Study Quality

There were *c* = 2 comparisons reported in *k* = 2 studies with a quality score of 7 or above. The analysis showed an effect in favor of guided self-help CBT compared to comparison groups for ED pathology, *g* = -0.64, 95%-CI [-0.92; -0.35] in samples with mixed ED diagnoses. No estimate could be obtained for binge-eating frequency, as all comparisons measuring binge eating were excluded.

Different Values for ρ

Across different values for ρ, CBT was associated with less ED pathology, but not a lower frequency of binge eating than comparison groups. There were significant differences between CBT and waitlist comparison groups for both ED pathology and binge-eating frequency in samples with mixed ED diagnoses. While CBT was superior to TAU only for ED pathology, there were no differences between CBT and active treatment for either outcome. Guided self-help CBT was associated with a less ED pathology and a lower binge-eating

frequency than comparison groups, whereas there were no differences between therapist-led CBT and comparison groups. Baseline eating disorder severity was not a moderator for either outcome.

Table S8. *Results of Sensitivity Analysis using Different Values of ρ for Studies Investigating Mixed Eating Disorder Diagnoses*

		eating disorder pathology			binge-eating frequency		
main analysis	ρ	*g*	95%-CI	95%-PI	*g*	95%-CI	95%-PI
	-0.9	-0.52	[-0.71; -0.33]	[-0.99; -0.06]	-0.16	[-0.36; 0.04]	[-0.45; 0.14]
	-0.7	-0.52	[-0.71; -0.33]	[-0.99; -0.06]	-0.16	[-0.36; 0.04]	[-0.45; 0.14]
	-0.5	-0.52	[-0.71; -0.33]	[-0.99; -0.06]	-0.16	[-0.36; 0.04]	[-0.45; 0.14]
	-0.3	-0.52	[-0.71; -0.33]	[-0.99; -0.06]	-0.16	[-0.36; 0.04]	[-0.45; 0.14]
	-0.1	-0.52	[-0.71; -0.33]	[-0.99; -0.06]	-0.16	[-0.36; 0.04]	[-0.45; 0.14]
	0.1	-0.52	[-0.71; -0.33]	[-0.99; -0.05]	-0.16	[-0.36; 0.04]	[-0.46; 0.14]
	0.3	-0.53	[-0.72; -0.33]	[-1.01; -0.04]	-0.17	[-0.38; 0.04]	[-0.50; 0.16]
	**0.5**	**-0.53**	**[-0.73; -0.33]**	**[-1.04; -0.02]**	**-0.18**	**[-0.39; 0.04]**	**[-0.53; 0.18]**
	0.7	-0.53	[-0.74; -0.33]	[-1.07; -0.0001]	-0.19	[-0.40; 0.03]	[-0.56; 0.19]
	0.9	-0.54	[-0.75; -0.32]	[-1.10; 0.22]	-0.19	[-0.41; 0.02]	[-0.58; 0.19]
type of comparison group	ρ	*g*	95%-CI	95%-PI	*g*	95%-CI	95%-PI
waitlist	-0.9	-0.76	[-1.07; -0.46]	[-1.42; -0.11]	-0.41	[-0.75; -0.06]	[-0.75; -0.06]
	-0.7	-0.76	[-1.07; -0.46]	[-1.42; -0.11]	-0.41	[-0.75; -0.06]	[-0.75; -0.06]

		eating disorder pathology			binge-eating frequency		
	-0.5	-0.76	[-1.07; -0.46]	[-1.42; -0.11]	-0.41	[-0.75; -0.06]	[-0.75; -0.06]
	-0.3	-0.76	[-1.07; -0.46]	[-1.42; -0.11]	-0.41	[-0.75; -0.06]	[-0.75; -0.06]
	-0.1	-0.76	[-1.07; -0.46]	[-1.42; -0.11]	-0.41	[-0.75; -0.06]	[-0.75; -0.06]
	0.1	-0.76	[-1.07; -0.46]	[-1.42; -0.11]	-0.41	[-0.75; -0.06]	[-0.75; -0.06]
	0.3	-0.76	[-1.07; -0.46]	[-1.42; -0.11]	-0.41	[-0.75; -0.06]	[-0.75; -0.06]
	**0.5**	**-0.76**	**[-1.07; -0.46]**	**[-1.42; -0.11]**	**-0.40**	**[-0.75; -0.06]**	**[-0.75; -0.06]**
	0.7	-0.76	[-1.07; -0.46]	[-1.42; -0.11]	-0.40	[-0.74; -0.06]	[-0.75; -0.06]
	0.9	-0.76	[-1.07; -0.46]	[-1.42; -0.11]	-0.39	[-0.74; -0.05]	[-0.74; -0.05]
active treatment	-0.9	-0.26	[-0.62; 0.09]	[-0.95; 0.42]	0.12	[-0.26; 0.50]	[-0.26; 0.50]
	-0.7	-0.26	[-0.62; 0.09]	[-0.95; 0.42]	0.12	[-0.26; 0.50]	[-0.26; 0.50]
	-0.5	-0.26	[-0.62; 0.09]	[-0.95; 0.42]	0.12	[-0.26; 0.50]	[-0.26; 0.50]
	-0.3	-0.26	[-0.62; 0.09]	[-0.95; 0.42]	0.12	[-0.26; 0.50]	[-0.26; 0.50]
	-0.1	-0.26	[-0.62; 0.09]	[-0.95; 0.42]	0.12	[-0.26; 0.50]	[-0.26; 0.50]
	0.1	-0.26	[-0.62; 0.09]	[-0.95; 0.42]	0.12	[-0.26; 0.50]	[-0.26; 0.50]
	0.3	-0.26	[-0.62; 0.09]	[-0.95; 0.42]	0.12	[-0.26; 0.50]	[-0.26; 0.50]

			eating disorder pathology			binge-eating frequency	
	**0.5**	**-0.26**	**[-0.62; 0.09]**	**[-0.95; 0.42]**	**0.12**	**[-0.26; 0.50]**	**[-0.26; 0.50]**
	0.7	-0.26	[-0.62; 0.09]	[-0.95; 0.42]	0.12	[-0.26; 0.50]	[-0.26; 0.50]
	0.9	-0.26	[-0.62; 0.09]	[-0.95; 0.42]	0.12	[-0.26; 0.50]	[-0.26; 0.50]
treatment as usual	-0.9	-0.47	[-0.93; -0.01]	[-1.21; 0.27]	-0.16	[-0.34; 0.02]	[-0.34; 0.02]
	-0.7	-0.47	[-0.93; -0.01]	[-1.21; 0.27]	-0.16	[-0.34; 0.02]	[-0.34; 0.02]
	-0.5	-0.47	[-0.93; -0.01]	[-1.21; 0.27]	-0.16	[-0.34; 0.02]	[-0.34; 0.02]
	-0.3	-0.47	[-0.93; -0.01]	[-1.21; 0.27]	-0.16	[-0.34; 0.02]	[-0.34; 0.02]
	-0.1	-0.47	[-0.93; -0.01]	[-1.21; 0.27]	-0.16	[-0.34; 0.02]	[-0.34; 0.02]
	0.1	-0.47	[-0.93; -0.01]	[-1.21; 0.27]	-0.16	[-0.34; 0.02]	[-0.34; 0.02]
	0.3	-0.47	[-0.93; -0.01]	[-1.21; 0.27]	-0.16	[-0.34; 0.02]	[-0.34; 0.02]
	**0.5**	**-0.47**	**[-0.93; -0.01]**	**[-1.21; 0.27]**	**-0.17**	**[-0.35; 0.01]**	**[-0.35; 0.02]**
	0.7	-0.47	[-0.93; -0.01]	[-1.21; 0.27]	-0.17	[-0.35; 0.01]	[-0.35; 0.01]
	0.9	-0.47	[-0.93; -0.01]	[-1.21; 0.27]	-0.17	[-0.35; 0.01]	[-0.36; 0.01]

		eating disorder pathology			binge-eating frequency		
treatment format	ρ	0.9	95%-CI	95%-PI	*g*	95%-CI	95%-PI
therapist-led	-0.9	-0.39	[-0.84; 0.07]	[-1.09; 0.31]	0.09	[-0.33; 0.52]	[-0.40; 0.59]
	-0.7	-0.39	[-0.84; 0.07]	[-1.09; 0.31]	0.09	[-0.33; 0.52]	[-0.40; 0.59]
	-0.5	-0.39	[-0.84; 0.07]	[-1.09; 0.31]	0.09	[-0.33; 0.52]	[-0.40; 0.59]
	-0.3	-0.39	[-0.84; 0.07]	[-1.09; 0.31]	0.09	[-0.33; 0.52]	[-0.40; 0.59]
	-0.1	-0.39	[-0.84; 0.07]	[-1.09; 0.31]	0.09	[-0.33; 0.52]	[-0.40; 0.59]
	0.1	-0.39	[-0.84; 0.07]	[-1.09; 0.32]	0.09	[-0.33; 0.52]	[-0.40; 0.59]
	0.3	-0.39	[-0.85; 0.07]	[-1.10; 0.32]	0.09	[-0.35; 0.52]	[-0.42; 0.59]
	**0.5**	**-0.39**	**[-0.86; 0.08]**	**[-1.11; 0.33]**	**0.07**	**[-0.36; 0.51]**	**[-0.44; 0.59]**
	0.7	-0.39	[-0.87; 0.08]	[-1.13; 0.35]	0.06	[-0.37; 0.49]	[-0.45; 0.57]
	0.9	-0.39	[-0.88; 0.09]	[-1.15; 0.36]	0.05	[-0.37; 0.47]	[-0.45; 0.55]
guided self-help	-0.9	-0.57	[-0.82; -0.32]	[-1.16; 0.02]	-0.24	[-0.48; -0.002]	[-0.59; 0.10]
	-0.7	-0.57	[-0.82; -0.32]	[-1.16; 0.02]	-0.24	[-0.48; -0.002]	[-0.59; 0.10]
	-0.5	-0.57	[-0.82; -0.32]	[-1.16; 0.02]	-0.24	[-0.48; -0.002]	[-0.59; 0.10]
	-0.3	-0.57	[-0.82; -0.32]	[-1.16; 0.02]	-0.24	[-0.48; -0.002]	[-0.59; 0.10]

		eating disorder pathology			binge-eating frequency		
	-0.1	-0.57	[-0.82; -0.32]	[-1.16; 0.02]	-0.24	[-0.48; -0.002]	[-0.59; 0.10]
	0.1	-0.57	[-0.82; -0.32]	[-1.16; 0.02]	-0.24	[-0.49; -0.002]	[-0.59; 0.11]
	0.3	-0.58	[-0.83; -0.32]	[-1.17; 0.02]	-0.25	[-0.50; -0.002]	[-0.61; 0.11]
	**0.5**	**-0.58**	**[-0.83; -0.32]**	**[-1.19; 0.03]**	**-0.25**	**[-0.50; -0.01]**	**[-0.62; 0.12]**
	0.7	-0.58	[-0.84; -0.32]	[-1.20; 0.04]	-0.26	[-0.50; -0.02]	[-0.62; 0.11]
	0.9	-0.59	[-0.85; -0.32]	[-1.22; 0.05]	-0.26	[-0.49; -0.03]	[-0.62; 0.10]

baseline severity	ρ	*b*	*z*	*p*	*b*	*z*	*p*
	-0.9	0.01	0.37	.713	-0.15	-1.52	.129
	-0.7	0.01	0.37	.713	-0.15	-1.52	.129
	-0.5	0.01	0.37	.713	-0.15	-1.52	.129
	-0.3	0.01	0.37	.713	-0.15	-1.52	.129
	-0.1	0.01	0.37	.713	-0.15	-1.52	.129
	0.1	0.01	0.37	.713	-0.15	-1.51	.130
	0.3	0.01	0.37	.713	-0.15	-1.48	.138
	**0.5**	**0.01**	**0.37**	**.713**	**-0.14**	**-1.43**	**.153**

	eating disorder pathology			binge-eating frequency		
0.7	0.01	0.37	.713	-0.13	-1.36	.175
0.9	0.01	0.37	.714	-0.12	-1.28	.201

Note. Results in bold indicate results reported in the main results section.

Funnel Plots

Figure S37. *Funnel Plot for Studies Investigating Anorexia Nervosa: Eating Disorder Pathology*

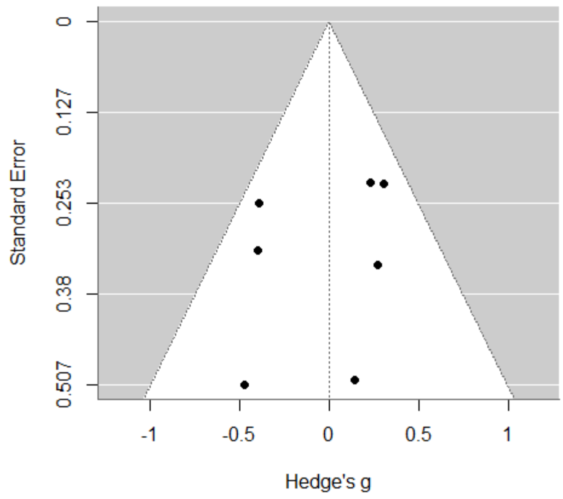

Note. -

Figure S38. *Funnel Plot for Studies Investigating Anorexia Nervosa: Body Mass Index*

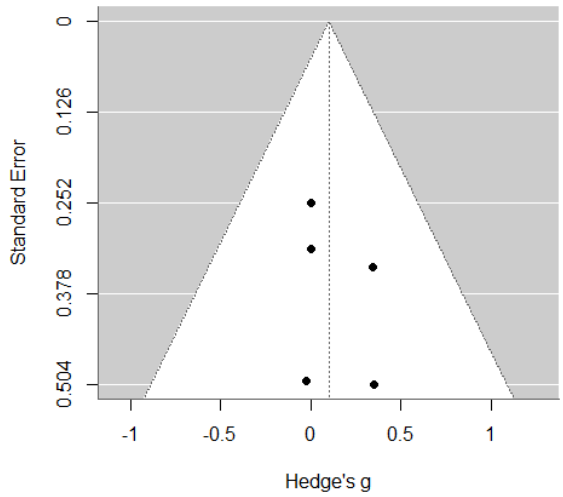

Note. -

Figure S39. *Funnel Plot for Studies Investigating Bulimia Nervosa: Eating Disorder Pathology*

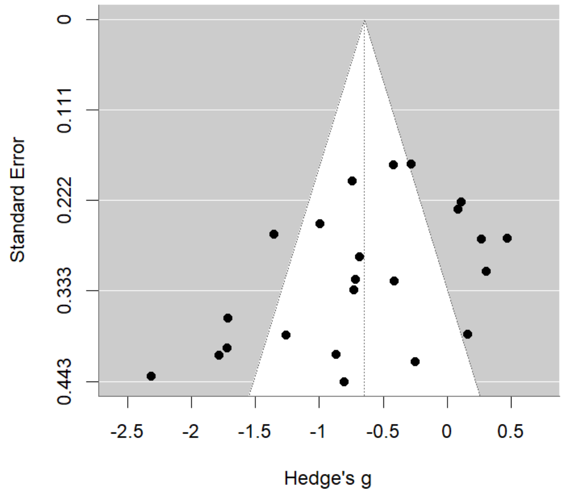

Note. -

Figure S40. *Funnel Plot for Studies Investigating Bulimia Nervosa: Binge-Eating Frequency*

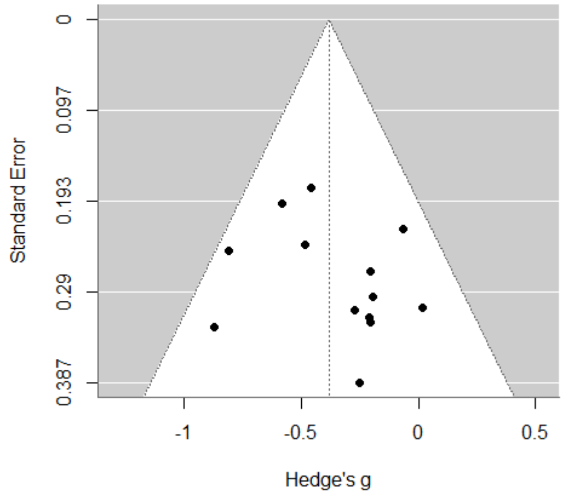

Note. -

Figure S41. *Funnel Plot for Studies Investigating Bulimia Nervosa: Frequency of Compensatory Behavior*

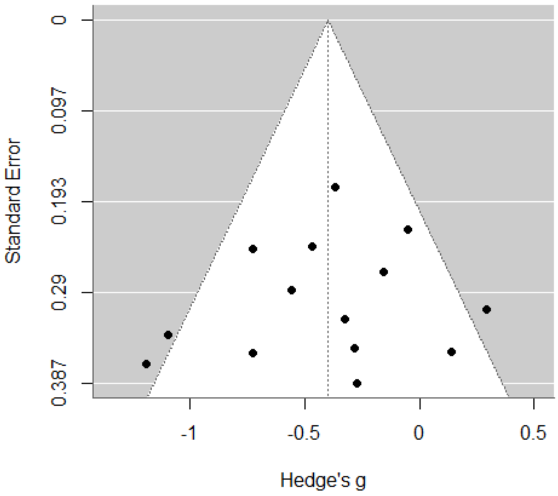

Note. -

Figure S42. *Funnel Plot for Studies Investigating Binge-Eating Disorder: Eating Disorder Pathology*

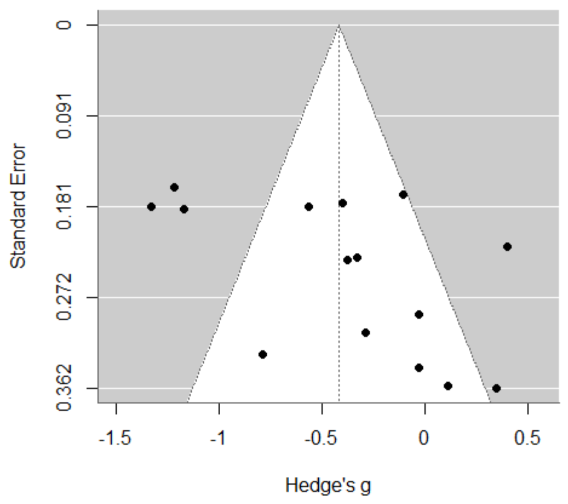

Note. -

Figure S43. *Funnel Plot for Studies Investigating Binge-Eating Disorder: Binge-Eating Frequency*

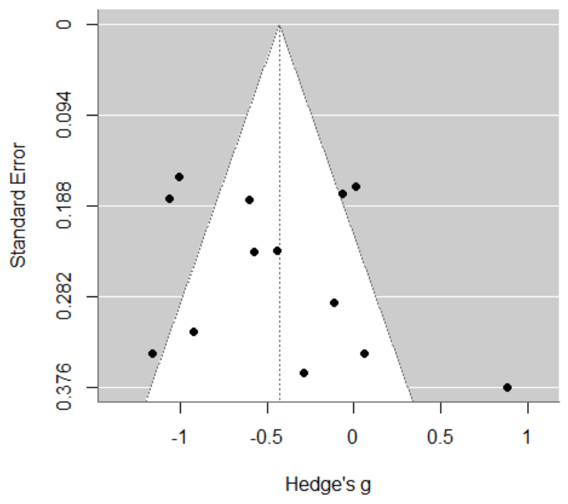

Note. -

Figure S44. *Funnel Plot for Studies Investigating Mixed Eating Disorders: Eating Disorder Pathology*

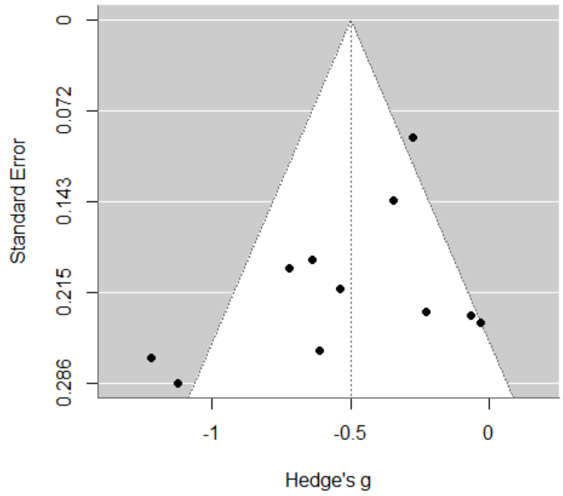

Note. -

Figure S45. *Funnel Plot for Studies Investigating Mixed Eating Disorders: Binge-Eating Frequency*

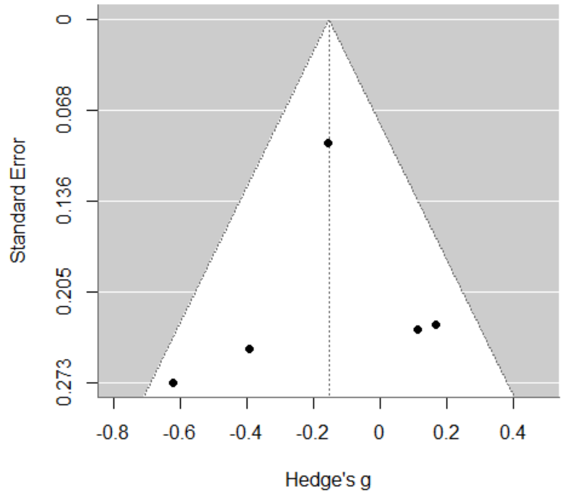

Note. –

Non-English Citations

Bravo Ferrón, S. (2006). Eficacia de un manual de autoayuda guiada por la enfermera especialista en salud mental como complemento a la Terapia Cognitivo Conductual en el tratamiento de pacientes diagnosticados de Bulimia Nerviosa. *NURE investigación; Revista Científica de enfermería*, *23*.

English Abstract

Bulimia nervosa is currently an important public health problem in the Comunidad de Madrid, with a prevalence of 3,7% of the teenage female population. During the last 10 years, the BN incidence has multiplied by three, reaching a rate of 50/1000000 women between 10 and 30 years old.

It has been associated with several pathologies, such as depression, anxiety and personality disorders (between 71 and 75% of patients present an anxiety disorders).

Including self-help manuals in Cognitive Behavioural Therapy (CBT) has been effective in other studies in a different context. Including the manual here could be beneficial for patients diagnosed as suffering BN.

Objective: Analyze the short- and midterm BN treatment effectiveness of including a self-help manual in the CBT, guided by a specialist mental health nurse.

Method: Experimental study of a randomized control group.

Population: Patients diagnosed as having BN in the last year (in CBT), with or without pharmacological treatment.

Variables: Age, sex, evolution time, family support, IMC.

Intervention: inclusion of a self-help manual, together with CBT treatment in the experimental group, plus 1820 minute interview sessions.

Data analysis: comparison of averages at the beginning of the treatment and one and six months after finishing the treatment in both groups (control and experimental), using BITE (Bulimia Investigatory Test Edinburgh) and STAI (anxiety) questionnaires.
